# The peach volatilome modularity is reflected at the genetic and environmental response levels in a QTL mapping population

**DOI:** 10.1186/1471-2229-14-137

**Published:** 2014-05-19

**Authors:** Gerardo Sánchez, José Martínez, José Romeu, Jesús García, Antonio J Monforte, María L Badenes, Antonio Granell

**Affiliations:** 1 Instituto de Biología Molecular y Celular de Plantas (IBMCP), Universidad Politécnica de Valencia (UPV)-Consejo Superior de Investigaciones Científicas (CSIC), Ingeniero Fausto Elio s/n, 46022 Valencia, Spain; 2 Instituto Nacional de Tecnología Agropecuaria (INTA), Ruta N°9 Km 170, 2930 San Pedro, Buenos Aires, Argentina; 3 Instituto Valenciano de Investigaciones Agrarias (IVIA), Carretera Moncada-Náquera Km 4,5, 46113 Náquera, Valencia, Spain; 4 Instituto Murciano de Investigación y Desarrollo Agrario (IMIDA), C/ Mayor s/n, 30150 La Alberca, Murcia, Spain

## Abstract

**Background:**

The improvement of fruit aroma is currently one of the most sought-after objectives in peach breeding programs. To better characterize and assess the genetic potential for increasing aroma quality by breeding, a quantity trait locus (QTL) analysis approach was carried out in an F_1_ population segregating largely for fruit traits.

**Results:**

Linkage maps were constructed using the IPSC peach 9 K Infinium ® II array, rendering dense genetic maps, except in the case of certain chromosomes, probably due to identity-by-descent of those chromosomes in the parental genotypes. The variability in compounds associated with aroma was analyzed by a metabolomic approach based on GC-MS to profile 81 volatiles across the population from two locations. Quality-related traits were also studied to assess possible pleiotropic effects. Correlation-based analysis of the volatile dataset revealed that the peach volatilome is organized into modules formed by compounds from the same biosynthetic origin or which share similar chemical structures. QTL mapping showed clustering of volatile QTL included in the same volatile modules, indicating that some are subjected to joint genetic control. The monoterpene module is controlled by a unique locus at the top of LG4, a locus previously shown to affect the levels of two terpenoid compounds. At the bottom of LG4, a locus controlling several volatiles but also melting/non-melting and maturity-related traits was found, suggesting putative pleiotropic effects. In addition, two novel loci controlling lactones and esters in linkage groups 5 and 6 were discovered.

**Conclusions:**

The results presented here give light on the mode of inheritance of the peach volatilome confirming previously loci controlling the aroma of peach but also identifying novel ones.

## Background

Traditionally, peach [*Prunus persica* (L.) Batsch] breeding programs have been focused on obtaining elite genotypes that are highly productive, resistant to pathogen and plagues, and which produce large fruit with an overall good appearance throughout most of the season (early and late cultivars). As a result, many cultivars with excellent agronomic performance have been developed. Nevertheless, breeding for agronomic traits often occurs in detriment of the organoleptic quality of the fruit, as was demonstrated in the cases of “greek basil”, strawberry, and tomato, where most of the typical aromas were lost during recent breeding processes [1-3]. In peach, the decrease in organoleptic fruit quality is perceived by consumers as the principal cause of dissatisfaction [4]. A likely consequence of this is the low consumption of peaches when compared with other fruits like apple and banana [5]. Early studies established that fruit aroma, along with flesh firmness and color, is the main attribute that consumers use to judge peach quality [6] and one of the main factors affecting peach prices in the market [7]. Therefore, genetic improvement of organoleptic fruit quality could lead not only to an increased consumption but would also add value to this food commodity.

Peach breeding is hindered by the reduced genetic variability in the available germplasm and by certain aspects of the physiology of the peach tree, such as its short blossoming time and juvenile phase of 2 to 3 years [8]. Thus, peach breeding not only requires an investment of time but also results in high operating costs associated with the maintenance of the trees in the field until the fruit can be evaluated. Consequently, the implementation of marker-assisted selection (MAS) becomes, almost exclusively, the only feasible option for reducing costs while at the same time improving breeding efficiency. However, the improvement of fruit flavor is not an easy task since the aroma is formed by the qualitative and quantitative combination of a large number of volatile organic compounds (VOCs) released by the fruit. To add complexity, VOCs also contribute to the taste of the fruit acting in combination with sugars and organic acids. In the case of peach, around 100 compounds have been described thus far ([9] and references within), but few seem to contribute to the aroma of the fruit [10]. Among these volatiles, lactones appear to be the main contributors to peach aroma [10,11], and in particular γ-decalactone, an intramolecular ester with an aroma described as “peach-like” [12]. Esters such as (Z)-3-hexenyl acetate, (E)-2-hexen-1-ol acetate, and ethyl acetate may contribute “fruity” notes to the overall fruit aroma [10,12,13], while terpenoid compounds like linalool and β-ionone may provide “floral” notes [10,13,14]. On the other hand, the aroma of the lipid-derived compounds, such as (Z)-3-hexenal and (E)-2-hexenal, have been described as “green” notes [12], and are usually associated with unripe fruit. Several studies have demonstrated that aroma formation in peach is a dynamic process, as volatiles change dramatically during maturity and ripening [15-18], cold storage [19], postharvest treatments [17,20], culture techniques, and management of the trees in the field [21].

The large impact that fruit VOCs have on peach acceptability and marketability has encouraged several groups to find genes and loci that control aroma production. Recently, Eduardo et al. [22] performed a QTL analysis for 23 volatile compounds, most of which contribute to peach fruit aroma. Among the QTL identified, a locus with major effects on the production of two monoterpene compounds was described in LG4 and, moreover, the co-localization with terpene synthase genes was shown [22]. Earlier the same group performed a microarray-based RNA profiling analysis to describe the changes in aroma-related gene expression during ripening [23]. In addition, an EST library was analyzed to find a set of candidate genes expressed in peach fruit related to the synthesis of different volatile compounds [24]. Additional studies targeted literature-derived candidate genes to analyze their involvement in the production of lactones, esters [17,25,26], and carotenoid-derived volatiles [27]. More recently, novel candidate genes for the control of diverse groups of volatiles were proposed by using a non-targeted genomic approach which analyzed the correlation between transcript and compound levels [28]. A high-quality genome of peach is currently available [29], and it is envisaged that next-generation sequencing technologies such as RNA-seq will soon be applied to discovering more genes related to the aroma of peach. In this context, additional studies delimiting the chromosome regions linked to aroma formation will help to interconnect this emerging wealth of information and thereby elucidate aroma-associated gene function in peach.

The recent development of a 9K Single-Nucleotide Polymorphism (SNP) Infinium II array by The International Peach SNP Consortium (IPSC) anchored in the genome [30] has facilitated the rapid development of linkage maps which had been hampered to a certain extent by the low genetic variability of intraspecific populations [8]. Complementarily, the recent advances in high-throughput technologies based on gas chromatography–mass spectrometry (GC-MS) for volatile profiling [31] have enabled researchers to describe the peach volatilome at a more exhaustive level [9]. Similar profiling platforms combined with natural variability and mapping information have been applied recently to large-scale analyses of volatile QTL in strawberry [32] and tomato [33].

In this study we have taken advantage of a high-throughput SNP genotyping array coupled to a GC-MS-based metabolomic approach to discover QTL for volatile compounds in peach fruit. The data presented here confirms a locus controlling linalool and p-mentha-1-en-9-al as described previously [22], but also shows that this locus controls the content of additional monoterpene compounds. Moreover, novel sources of variability in LG5 and LG6 were identified for the most important aroma-related compounds in peach (i.e., lactones and esters), which could be used for the improvement of peach flavor. The results presented here strengthen the current knowledge regarding the genetic control of aroma and confirm the genetic potential for improving peach flavor by marker-assisted breeding.

## Methods

### Plant material

The peach progeny studied herein was an F_1_ population obtained from a cross between the genotypes ‘MxR_01’ and ‘Granada’. ‘MxR_01’ is a freestone, melting-flesh peach which was obtained through the IVIA (*Instituto Valenciano de Investigaciones Agrarias*) breeding program and selected from the cross between the melting peach ‘RedCandem’ (obtained by a U.S breeding program) and the non-melting peach ‘Maruja’ (a traditional Spanish variety). ‘Granada’ is a clingstone, non-melting peach with a low chilling requirement obtained from a Brazilian breeding program [34]. The female parent of ‘Granada’ is Conserva 471, while the male parent is unknown. Replicate clones derived from each seedling in the collection were cultivated in three experimental orchards: two situated in Spain's Murcia region, “El Jimeneo” (EJ) and “Aguas Amargas” (AA), and another in Valencia, Spain at the IVIA. EJ is located at an altitude of 80m at latitude: 37° 45' 31,5 N; longitude: 1° 01' 35,1 O. AA is located at an altitude of 344m at latitude: 38° 31' N; longitude: 1° 31' O. IVIA is located at an altitude of 55m at latitude: 39° 34' N, longitude 0° 24' W. A total of 86 genotypes were grown at EJ, 74 at AA and 71 at the IVIA. The peach trees were implanted in 2009 in the three locations. Following the horticultural practices indicated in [35], the first harvest was obtained in 2011. Usually fruits from the first harvest are not representative of the full potential of the genotype and therefore was discarded. Fruits from the following season were used for the analyses.

Peach fruits from the F_1_ hybrids and parental genotypes were harvested from June to August, 2012. The harvest date (HD) for each genotype analyzed was expressed as the difference in days from the date of the earliest genotype. Fruits harvested at IVIA were analyzed only for fruit traits while fruits from EJ and AA were used for both fruit traits and volatile analyses as is described in a later section.

### Population genotyping and map construction

DNA was extracted from 50 mg of young leaves following the method of Doyle & Doyle [36]. The concentration of DNA was checked by comparison with standard DNA labels in agarose gels and with Quant-iT™ PicoGreen H Assay (Life Technologies, Grand Island, NY, USA). Samples were genotyped using the IPSC peach 9 K Infinium® II array, which includes around 9000 peach SNP markers [30], at the Genotyping and Genetic Diagnosis Unit (Health Research Institute, INCLIVA, Valencia, Spain).

Polymorphic markers were codified as cross-pollinator (CP) for linkage map construction using JoinMap® V4 (Kyazma B.V, Netherlands) [37].

Monomorphic SNPs and SNPs with more than 5% missing data were removed. For genetic map construction, we followed the two-way pseudo-test cross approach [38]. SNPs that were homozygous in one parent and heterozygous in the other (and therefore segregating 1:1 through the progeny) were selected to generate a genetic map for each parent, discarding SNPs that were heterozygous for both parents. Linkage groups with an LOD of 6.0 to 8.0 were selected. Map construction was performed using the regression mapping algorithm [39] and the default JoinMap® parameters (Rec = 0.40, LOD = 1, Jump = 5.0, and ripple = 1). The order of the markers in each linkage map was double-checked with MAPMAKER/EXP version 3.0b [40]. The Kosambi mapping function was used to convert recombination frequencies into map distances. Maps were drawn with MapChart 2.2 [41].

### Fruit and volatile analyses

A total of 15 fruits were harvested at nearly “harvest ripe” (also know as “ready to buy”) stage, according to visual and firmness inspections by expert operators, from trees at each of the EJ, AA, and IVIA locations. Fruits were transported at room temperature (RT, 20–28°C) to the IBMCP laboratories in Valencia, Spain where they were also maintained at RT to complete a period of 24 h in total. This period would allow the fruits to ripen to “consumption ripe” (or “ready to eat”) stage, as was later determined by maturity analyses. The most homogeneous fruits with no evident defects (disease, damage, etc.) were picked for maturity analysis. The maturity parameters (peel ground color, flesh firmness, weight, and total soluble solids (SSC)) were analyzed as described previously [9] for fruit from EJ, AA, and IVIA. Fruit were weighed and peel ground color parameters (L, lightness; C, chroma; and H, color measured in hue degree) were recorded using a HunterLab ColorFlex colorimeter (Hunter Associates Laboratory, Inc., Reston, VA., U.S.A.). The flesh firmness was analyzed and in the case of fruits from EJ and AA, immediately after measurement, half of the fruit mesocarp was frozen in liquid nitrogen for subsequent volatile analysis. Finally, the SSC was analyzed in the remaining fruit mesocarp. To standardize the ripening stage, fruits with SSC > 11 and a peel ground color between 70° to 90° H degrees were selected for each genotype/location (4 to 10 fruits) for QTL analysis. For EJ, AA, and IVIA, only the maturity data from selected fruits were used for QTL analysis, as described later. For fruits from EJ and AA, frozen mesocarp samples of selected fruits were pooled and ground to powder in liquid nitrogen to obtain a composite sample (biological replicate) that was assessed three times for volatile analyses (technical replicates). Volatile compounds were analyzed from 500 mg of frozen tissue powder, following the method described previously [9]. The volatile analysis was performed on an Agilent 6890N gas chromatograph coupled to a 5975B Inert XL MSD mass spectrometer (Agilent Technologies), with GC-MS conditions as per Sánchez et al. [9]. A total of 43 commercial standards were used to confirm compound annotation. Volatiles were quantified relatively by means of the Multivariate Mass Spectra Reconstruction (MMSR) approach developed by Tikunov et al. [42]. A detailed description of the quantification procedure is provided in Sánchez et al. [9]. The data was expressed as log2 of a ratio (sample/common reference) and the mean of the three replicates (per genotype, per location) was used for all the analyses performed. The common reference consists of a mix of samples with non stoichiometry composition representing all genotypes analyzed (i.e. the samples were not weighted).

### Data and QTL analysis

The Acuity 4.0 software (Axon Instruments) was used for: hierarchical cluster analysis (HCA), heatmap visualization, principal component analysis (PCA), and ANOVA analyses.

Correlation network analysis was conducted with the Expression Correlation (http://www.baderlab.org/Software/ExpressionCorrelation) plug-in for the Cytoscape software [43]. Networks were visualized with the Cytoscape software, v2.8.2 (http://www.cytoscape.org).

Genetic linkage maps were simplified, eliminating co-segregating markers in order to reduce the processing requirements for the QTL analysis without losing map resolution. Maps for each parental were analyzed independently and coded as two independent backcross populations. For each trait (volatile or maturity related trait) and location, the QTL analysis was performed by single marker analysis and composite interval mapping (CIM) methods with Windows QTL Cartographer v2.5 [44]. A QTL was considered statistically significant if its LOD was higher than the threshold value score after 1000 permutation tests (at α = 0.05). Maps and QTL were plotted using Mapchart 2.2 software [41], taking one and two LOD intervals for QTL localization. The epistatic effect was assayed with QTLNetwork v2.1 [45] using the default parameters.

### Availability of supporting data

The data sets supporting the results of this article are included within the article (and its additional files).

## Results

### SNP genotyping and map construction

The IPSC 9 K Infinium ® II array [30], which interrogates 8144 marker positions, was used to genotype our mapping population at deep coverage. The raw genotyping data is provided in supplementary information (Additional file 1: Table S1). To analyze only high-quality SNP data, markers with four or more missing data (around 300 SNPs in all) were eliminated from the data set. Non-informative SNPs, i.e., those that are monomorphic and are therefore not segregating, were also eliminated, resulting finally in 3630 polymorphic markers. The marker segregation was tested against a normal Mendelian expectation ratio (1:1) in order to analyze segregation distortion, and those markers showing segregation distortion (stated at α < 0.05) were eliminated to avoid map artifacts. Thus, a total of 2865 polymorphic SNPs (40% of the total) were identified (Table 1) and selected for their respective map construction, from which 1970 segregated (1:1) for the ‘MxR_01’ parent and 895 for ‘Granada’.

**Table 1:** Summary of the SNPs analyzed for scaffolds 1–8

		**Polymorphic SNPs**			**SNPs selected**		**Map distance (cM)**		**Marker density (cM/marker)**	
**Scaffold**	**Total SNPs**	**SNPs (% of total)**	**MxR_01'**	**Granada'**	**MxR_01'**	**Granada'**	**MxR_01'**	**Granada'**	**MxR_01'**	**Granada'**
Sc1	959	319 (33%)	282	37	26	0	75.01	0	2.89	X
Sc2	1226	461 (38%)	273	188	0	13	0	59.08	X	4.54
Sc3	700	336 (48%)	325	11	40	0	87.28	0	2.18	X
Sc4	1439	496 (34%)	269	227	29	10	69.95	22.46	2.41	2.25
Sc5	476	243 (51%)	196	47	14	8	50.8	39.61	3.63	4.95
Sc6	827	364 (44%)	188	176	15	20	61.18	75.75	4.08	3.79
Sc7	686	318 (46%)	168	150	21	16	70.45	50.87	3.35	3.18
Sc8	804	328 (41%)	269	59	33	7	65.37	16.70	1.98	2.39
**TOTAL**	**7117**	**2865 (40%)**	**1970**	**895**	**178**	**74**	**480**	**264**		

For each scaffold, the total number of SNPs present in the array (Total SNPs) and the number of polymorphic markers with the percentage of the total (in parentheses) are indicated. Also, for each parental map (‘MxR_01’ and ‘Granada’), the total number of polymorphic SNPs found at each scaffold and the number of SNPs selected for map construction are indicated. Map distance (in cM) indicates the length of the linkage group corresponding to each chromosome and the total map distance covered for both parental maps. Marker density indicates the distance between contiguous markers (on average) in each map. X indicates those cases where there were not enough markers to build a genetic map and for which marker density could therefore not be calculated.

An example of the way we proceeded is shown in Additional file 2: Figure S1. A total of 282 polymorphic SNPs were located in scaffold (Sc) 1 of the peach genome assembly v1.0 segregating for the ‘MxR_01’ parental. Of these, 265 markers could be grouped and ordered in a single linkage group with several markers co-segregating in the same position (Additional file 2: Figure S1). One SNP for each position was selected (26 in all) to obtain a simplified map. Similarly, maps corresponding to the other scaffolds (3, 4, 5, 6, 7, and 8) were obtained with the exception of Sc2, for which the map was not consistent with the expected genome position and had large gaps (greater than 30 cM), and was discarded for being not suitable for QTL analysis. A total of 178 SNPs were located in the ‘MxR_01’ simplified map, representing a total distance of 480 cM (Table 1). The marker density varies between 1.98 cM/marker (for LG8) to 4.08 cM/marker (for LG6). On average, one marker per 2.94 cM was found in the ‘MxR_01’ map.

For ‘Granada’, a lower number of polymorphic markers was obtained as compared to ‘MxR_01’ (Table 1). Following the same strategy as described for ‘MxR_01’, the maps for Scs 2, 4, 5, 6, 7, and 8 were obtained for ‘Granada’. No map was obtained for Sc1 and Sc3. Only the linkage groups of Sc6 and Sc7 showed evenly distributed markers with good coverage (as shown below). The map obtained covered less distance compared to ‘MxR_01’ (264 vs 480 cM) with a lower marker density (3.52 vs 2.94 cM/marker on average).

### Evaluation of volatile variability in the mapping population

Volatile compounds were analyzed from the populations grown in the different agro-ecological zones: EJ and AA. As an example of the variability among fruits within the mapping population, pictures of several representative fruits grown at EJ are shown in Additional file 3: Figure S2. Genotypes growing at EJ ripened on average 7.9 days earlier as compared to AA (stated by ANOVA at α < 0.01), probably due to the warmer weather in AA compared with EJ, confirming that the two locations represent different environments.

**Figure 1:**
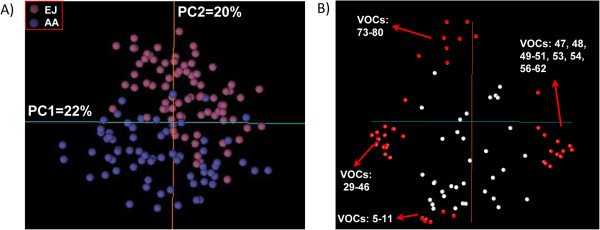
**Principal component analysis of the volatile data set. A)** Principal component analysis of the mapping population. Hybrids harvested at locations EJ and AA are indicated with different colors. **B)** Loading plots of PC1 and PC2. In red are pointed the volatiles that most accounted for the variability in the aroma profiles across PC1 and PC2 (numbered according to Additional file 4: Table S2).

A total of 81 volatiles were profiled (Additional file 4: Table S2). To assess the environmental effect, the Pearson correlation of volatile levels between the EJ and AA locations was analyzed. Around half of the metabolites (41) showed significant correlation, but only 17 showed a correlation higher than 0.40 (Additional file 4: Table S2), indicating that a large proportion of the volatiles are influenced by the environment. To get a deeper understanding of the structure of the volatile data set, a PCA was conducted. Genotypes were distributed in the first two components (PC1 and PC2 explaining 22% and 20% of the variance, respectively) without forming clear groups (Figure 1A). Genotypes located in EJ and AA were not clearly separated by PC1, although at extreme PC2 values, the samples tend to separate according to location, which points to an environmental effect. Loading score plots (Figure 1B) indicated that lipid-derived compounds (73–80, numbered according to Additional file 4: Table S2), long-chain esters (6, 9, and 11), and ketones (5, 7, and 8) along with 2-Ethyl-1-hexanol acetate (10) would be the VOCs most influenced by location (Figure 1B). According to this analysis, fruits harvested at EJ are expected to have higher levels of lipid-derived compounds, whereas long-chain esters, ketones and acetic acid 2-ethylhexyl ester should accumulate in higher levels in fruits harvested in AA. This result indicates that these compounds are likely the most influenced by the local environment conditions. On the other hand, PC1 separated the lines mainly on the basis of the concentration of lactones (49 and 56–62), linear esters (47, 50, 51, 53, and 54) and monoterpenes as well as other related compounds of unknown origin (29–46), so those VOCs are expected to have a stronger genetic control.

**Figure 2:**
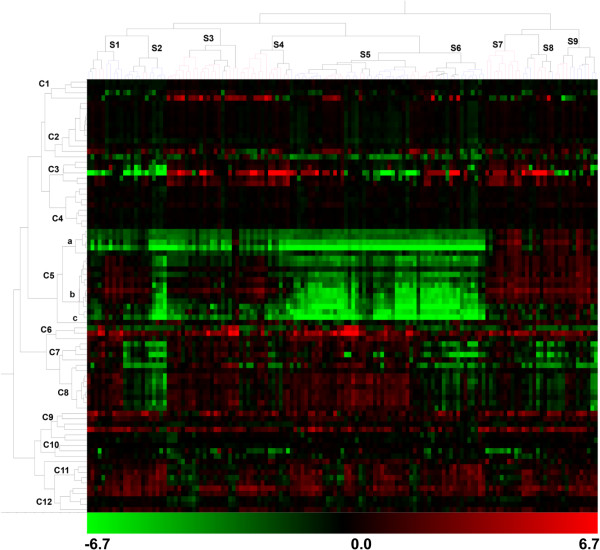
**Hierarchical cluster analysis and heatmap of volatiles and breeding lines.** On the volatile dendrogram (at left) are indicated the clusters obtained: C1-C12. The order of the volatile in the dendrogram corresponds to the one indicated in Additional file 1: Table S1. The upper dendogram corresponds to genotypes where the sample clusters are indicated by Additional file 1: Table S1, Additional file 4: Table S2, Additional file 5: Table S3, Additional file 6: Table S4, Additional file 7: Table S5, Additional file 10: Table S6, Additional file 11: Table S7, Additional file 12: Table S8, Additional file 13: Table S9. Data are expressed as a log2 of a ratio (sample/common reference). The scale used is indicated below the heatmap.

To analyze the relationship between metabolites, an HCA was conducted for volatile data recorded in both locations. This analysis revealed that volatile compounds grouped in 12 main clusters; most clusters had members of known metabolic pathways or a similar chemical nature (Figure 2, Additional file 4: Table S2). Cluster 2 is enriched with methyl esters of long carboxylic acids, i.e., 8–12 carbons (6, 9, 11, and 12), other esters (10 and 13), and ketones of 10 carbons (5, 7, and 8). Similarly, carboxylic acids of 6–10 carbons are grouped in cluster 3 (16–20). Cluster 4 mainly consists of volatiles with aromatic rings. In turn, monoterpenes (29–34, 37, 40, 41, 43, and 46) are grouped in cluster 5 with other ten-carbon compounds of as yet unknown origin. Ethanol and its acetate ester (47) clustered together in C6. Esters derived from acetyl-CoA and six-carbon alcohols (50–53) grouped in cluster 7. All detected lactones, with the exception of number 49, were grouped in cluster C8. Four carotenoid-derived volatiles (63–66) are found in C9, while lipid-derived compounds are grouped in C11 and C12. These results suggest that volatiles are co-regulated according to specific modules within the F_1_ population. The heat map revealed that the genotypes contain different combinations of these volatile modules. For example, the clusters of genotypes S7-S9 have high levels of volatiles belonging to C5 (which is rich in monoterpenes), whereas clusters S5 and S6 have low levels of these compounds (Figure 2). There are even genotypes, those of S1-S4, with different concentrations of volatiles in the C5 sub-clusters.

**Figure 3:**
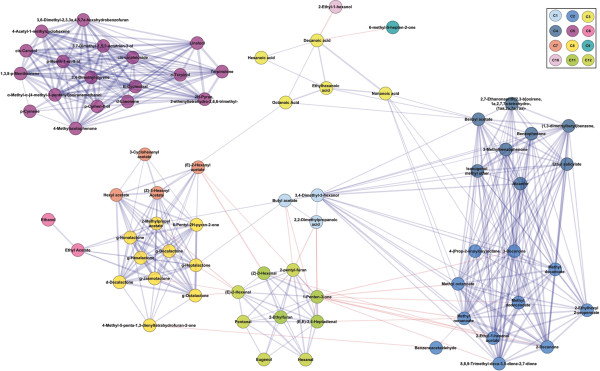
**Correlation network analysis of the data set.** The nodes representing volatiles are colored according to the cluster in which they were found (C1-C12) according to Figure 2, as indicated in the top-right corner. Positive and negative correlations are indicated with blue and red edges, respectively. Line thickness indicates correlation strength: the wider the line, the stronger the correlation.

A correlation network analysis (CNA) was conducted to further study the association between metabolites as well as the interrelationship between volatile modules. As expected, the volatiles that clustered together on the HCA were interconnected by positive interaction represented with blue lines in CNA (Figure 3). As previously reported [9], lactones and lipid-derived compounds showed negative interactions mainly through (E)-2-hexenal. Lactones showed high correlation with linear esters in C7 (50–53), ethyl acetate, and acetic acid butyl ester, the only ester in C1. Volatiles in C2 and C4 are interconnected with highly positive correlations. These two modules also showed positive correlation with C1 volatiles through the interaction with 3,4-dimethyl-3-hexanol. In turn, volatiles from C2 interact negatively with lipid-derived compounds in C11. On the other side, compounds in C5 are highly correlated to each other, but remain quite isolated from the rest of the compounds.

Taken together, these results suggest that, within our population, volatiles are co-regulated according to specific groups and that the genotypes have different combinations of volatile modules that may condition their aroma profiles.

### Genetic control of volatile compound synthesis and fruit quality traits

Peach volatile biosynthesis is highly dependent on fruit ripening stage [9,15-18,28]. For this reason, we also analyzed QTL for the main characteristics that have been traditionally used to asses the maturity stage of the peach fruit (and therefore quality): flesh firmness, weight, SSC, and peel color-related variables, thereby permitting the study of possible pleotropic effects of maturity on volatile production as well as the identification of loci involved in volatile production independent of maturity. Similarly, the Harvest Date (HD) was also included in our analysis, since it has been proposed that a major HD QTL at the south end of LG4 has a pleiotropic effect on volatile production in peach [22]. Additionally, as our mapping population segregated for melting/non-melting flesh (MnM) this trait was also included to analyze if there is a possible pleiotropic effect of the locus that controls flesh type on volatile production.

**Figure 4:**
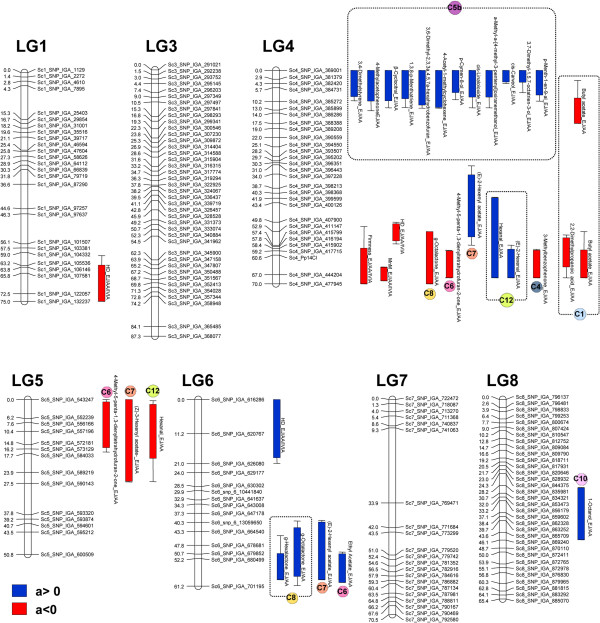
**Location of volatile QTL that are stable across location for the ‘MxR_01’ map.** Consistent QTL found at the locations EJ and AA (for volatiles) and EJ, AA, and IVIA (for HD, Firmness, and MnM traits) are shown. The QTL are colored according to the additive effect (a) that is exerted, red for negative a and blue for positive a. For volatile QTL, the circles with different colors (according to Figure 3) indicate the cluster that the controlled volatile belongs to. QTL for volatiles of the same cluster in the same linkage group are indicated with dashed-line rectangles. Bars and lines represent the 1-LOD and 2-LOD support intervals for each.

**Figure 5:**
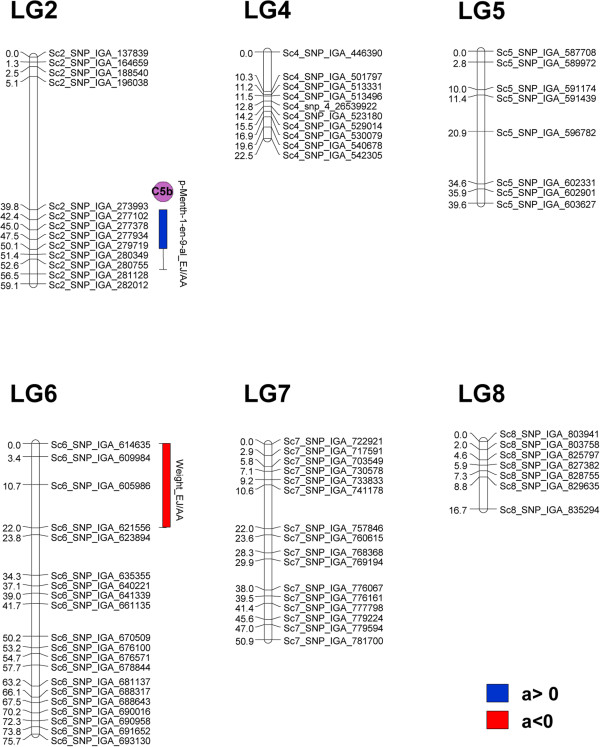
**Location of volatile QTL that are stable across location for the ‘Granada’ map.** The two consistent QTL found at the locations EJ and AA (for 3-cyclohexene-1-acetaldehyde,_a,4-dimethyl) and EJ, AA, and IVIA (for weight) are shown. The QTL are colored according to the additive effect (a) that is exerted, red for negative a and blue for positive a. For the volatile QTL, the colored circle (according to Figure 3) indicates the cluster that the controlled volatile belongs to. Bars and lines represent 1-LOD and 2-LOD support intervals.

A large number of QTL were detected for both fruit quality traits and volatile production (Additional file 5: Tables S3, Additional file 6: Table S4 and Additional file 7: Table S5). Most of them were detected in the ‘MxR_01’ map, probably due to the higher genetic diversity among the progenitors of ‘MxR_01’ compared to the progenitors of ‘Granada’. To graphically summarize the genetic control of volatiles, the likelihood of association between markers and compounds are presented as heatmaps in the supplementary data (Additional file 8: Figure S3 and Additional file 9: Figure S4). A proportion of the QTL identified (in general, between 20-40% depending on the trait) were consistently detected in at least two locations. These consistent QTL are presented in Figures 4 and 5.

In general, volatile compounds included in the same module showed similar LOD profiles in defined regions of chromosomes, suggesting the presence of loci that increase the production of whole volatile modules. For example in ‘MxR_01’, volatiles bellowing to the monoterpene-enriched cluster C5 showed similar LOD profiles on LG1, LG4, and LG5 in both locations (Additional file 8: Figure S3). Additionally, this analysis showed that LG8 of ‘MxR_01’ map exerted a very little control of the peach volatilome. On the contrary, the variability of compounds belonging to the C3 and C10 clusters (all formed by carboxylic acids and alcohols) were not associated with any genomic region, indicating an absence of allelic variability in the control of those compounds in the variability sources analyzed (Additional file 8: Figure S3).In the ‘MxR_01’ map, most of the consistent QTL were found forming two clusters in LG4 (Figure 4). At the upper end of LG4, QTL for 12 (out of 13) volatiles of cluster C5b were identified. At the southern end of LG4, QTL for lactones, esters, lipid-derived compounds, and other volatiles co-localizing with the loci controlling HD, MnM, and firmness were found. In the later QTL cluster, QTL controlling the production of the lactones 4-methyl-5-penta-1,3-dienyltetrahydrofuran-2-one and γ-octalactone showed negative additive effects, whereas those affecting two lipid-derived compounds (hexanal and (E)-2-hexenal), and a linear ester ((E)-2-hexen-1-ol acetate) showed a positive additive effect. Another cluster of QTL controlling the production of a lactone, an ester, and a lipid-derived compound was also found at the top of LG5. In addition, a cluster of QTL was found at the southern end of LG6, thus defining a locus controlling the content of two lactones (γ-hexalactone and γ-octalactone) and two esters (ethyl acetate and (E)-2-hexen-1-ol acetate) with the same direction of the additive effects.To further analyze the potential of these materials and information for volatile improvement, the epistatic effects between QTL were analyzed for all traits, but no significant effects were detected for the stable QTL indicated in Figure 4 (data not shown).

For the ‘Granada’ map, fewer QTL were found compared to ‘MxR_01’ (Additional file 6: Table S4), and only for the compound p-Menth-1-en-9-al a QTL stable locations was found (Figure 5). Also, a stable QTL for fruit weight explaining between 14-16% of the variance was identified in LG6 (Figure 5).

The raw phenotyping data set is provided as supplementary information (Additional file 10: Table S6).

### Assessment of the breeding population's potential for improvement

Since QTL analysis showed that the MnM locus co-localized with a cluster of volatile QTL (Figure 4), we compared the volatile profile of melting and non-melting genotypes within our population. Melting and non-melting peaches showed different levels of volatiles with QTL co-localizing in that region (Additional file 11: Table S7). According to the direction of the additive effects observed, non-melting peaches showed higher levels of not only γ-octalactone and 4-methyl-5-penta-1,3-dienyltetrahydrofuran-2-one, but also of other six lactones (Additional file 11: Table S7). Similarly, Butyl acetate and 2,2-dimethyl-propanoic acid levels were higher in non-melting peaches compared to melting ones. On the contrary, non-melting genotypes showed lower levels of hexanal and (E)-2-hexenal along with other lipid-derived compound (pentanal).

The genotypes showed a similar trend of ripening in EJ, AA, and IVIA, with the HD proving to be highly correlated between locations (r = 0.94 to 0.97). According to the mean HD across the three locations, the genotypes were divided into early, medium, and late season. In our population, around half of the peaches were melting and the other half non-melting (54% and 46%, respectively). Since the QTL for HD with major effects was found near the MnM locus, the effect of this linkage was analyzed in our breeding population. As expected due to the direction of the additive effects, early genotypes tend to be melting type (83%), while among the late genotypes most of the peaches are non-melting (79%, Additional file 12: Table S8). The potential for predicting fruit type was assessed. The genotypes were divided according to the ideotype of the two markers closest to the MnM locus (Sc4_SNP_IGA_444204 and Sc4_SNP_IGA_477945). In the group with ideotypes corresponding to melting peaches, 96% of the genotypes were actually phenotyped as melting type. In the group predicted to be non-melting according the ideotype, 83% were actually phenotyped as such.

To evaluate the potential for volatile improvement, the breeding population was divided according to ideotype at the different loci controlling aroma production. For the locus controlling most of the monoterpenes of C5b (Figure 4), the population was divided according to the ideotype of the region expanding the QTL in LG4 (Sc4_SNP_IGA_369001 to Sc4_SNP_IGA_386286). The levels of all volatiles were compared between the group expected to have high levels of these compounds and the other group formed by the rest of the genotypes (i.e., having the contrary ideotype or recombinants in that region). The expected rich-monoterpene ideotype group showed high levels for all the compounds in C5b as well as for the rest of the monoterpenes in C5 (Additional file 13: Table S9). As a side effect, the monoterpene-rich group showed lower levels of butyl acetate, as a QTL with the opposite effect was located near the tagged locus (Figure 4).

Similarly, the genotypes were divided according to the ideotype at the three loci that showed QTL for lactones in LG4 (Sc4_SNP_IGA_411147 to Sc4_SNP_IGA_477945), LG5 (Sc5_SNP_IGA_543247 to Sc5_SNP_IGA_584033), and LG6 (Sc6_snp_6_13059650 to Sc6_SNP_IGA_701195). Only four genotypes have a rich-lactone ideotype, all are non-melting, medium- (three genotypes) or late- (one 1 genotype) season peaches. This group has higher mean levels of five lactones compared to the rest of the genotypes (Additional file 14: Table S10).

## Discussion

As part of our ongoing efforts dedicated to the identification of genes and loci controlling important fruit-quality traits in peach, we studied the genetic control of aroma production and its relationship to other fruit quality characteristics. In this work, we took advantage of high-throughput genotyping and metabolite-profiling technologies in order to perform a large-scale QTL analysis in a F1 breeding population. One of our breeding goals is to improve the peach quality by enhancing the fruit aroma. To achieve this we included ‘Maruja’ genotype as ancestor in our breeding program, since it is a traditional Spanish variety known for its intense aroma. Our previous work [28], revealed that the parentals of the F1 population exhibit contrasting volatile profiles (more than 50% of the volatiles showed significant differences between parental), suggesting that this population was suitable for QTL analysis.

### Map construction using high-throughput SNP genotyping

Despite the wide genome coverage represented in the IPSC peach 9 K SNP array [30], chromosome 2 in the ‘MxR_01’ map and chromosomes 1 and 3 in the ‘Granada’ map did not have enough polymorphic SNP markers to obtain a minimum genetic map (Table 1, Figure 4 and Figure 5). In the case of ‘Granada’, linkage maps covering entire chromosomes were only obtained for chromosomes 6 and 7, whereas only partial coverage linkage groups were obtained for the rest of the chromosomes. The most likely explanation for the extensive homozygosity detected for chromosome 2 in ‘MxR_01’ is identity-by-descent, i.e., ‘Maruja’ and ‘RedCandem’ share at least a same copy of chromosome 2, and that pair was inherited by ‘MxR_01’. Since ‘Maruja’ is a traditional variety whose pedigree is unknown, it is therefore not possible to verify this hypothesis. The male parental of ‘Granada’ is also unknown [34], so it is possible that this genotype is self-pollinated, which might explain the extensive homozygosity found. The putative high homozygosity of chromosome 2 of ‘MxR_01’ and in several chromosomes of ‘Granada’ avoids the detection of QTL in those chromosomes. Indeed, as in any QTL analysis, the results obtained here are limited to the source of variability analyzed. Therefore, our results must be interpreted taking into account these facts.

The quality of the linkage map depends on the characteristics of the population used (population type, number of individuals genotyped, the genetic origin of the parentals, etc.) but also is related to the power of the genotyping platform utilized. The F_1_ population analyzed by Eduardo et al. [22] was also genotyped with the IPSC 9K SNP array and also showed a low number of polymorphic SNPs (1748 in total vs. the 2864 SNPs found here, Table 1), but the total genetic distances are comparable (405 cM and 228 cM in the two parental maps vs. 480 cM and 276 cM in the maps obtained here, Table 1). An alternative high-density SNP genotyping approach based on parent sequencing for SNP discovery was used for the detection of peach quality trait QTL [46]. In that case, the number of polymorphic markers (1775 SNPs) and the map coverage (422 cM and 369 cM) reported were comparable to our results, although the map was denser (0.81 cM/markers on average vs. 3.87 and 2.94 cM/marker for each map in this study). SNP genotyping chips are an inflexible assay that could be subject to assortment bias, i.e., they may be suitable for a certain sample of germplasm but not appropriate for other samples. In our case, we cannot discard whether the lack of polymorphic SNPs in certain chromosomes is caused by actual homozygosis or by a design bias of the chip. Currently, genotype-by-sequence technologies [47] could allow assortment bias to be overcome.

### The monoterpene module is controlled by a main locus while lactones and other linear esters showed several QTL

To get a first insight into the structure of the data set, a series of correlation-based analyses (HCA and CNA) and a data reduction method (PCA) were conducted (Figures 1, 2 and 3). Previously, we analyzed the correlation patterns of volatiles in a complex sample set (formed by four genotypes analyzed in different locations, at different maturity stages, and after a post-harvest treatment) to define groups of co-regulated compounds [9]. Here, the correlation-based analyses also showed that the volatile complement in ripe fruits from genetically diverse siblings is highly organized into modules (Figures 2 and 3) and the co-regulation patterns found are markedly similar to those previously described. However, the novel results presented here reveal that several of the co-regulated groups are not necessarily genetically controlled or, at the very least, are strongly affected by the environment. As regards environmental control, the PCA suggests a group of compounds that account for a separation among locations (Figure 1) and therefore reflect the influence of environment on volatile production in our population. To further support the importance of the environment, only 50% of the volatiles analyzed showed significant correlation between locations (Additional file 4: Table S2). Conversely, PCA showed that lactones, esters, and monoterpenes accounted for the separation among genotypes independent of location, which suggests that these volatiles are under significant genetic control (Figure 1). Nevertheless, the possibility that a ripening effect also contributes to the separation observed could not be dismissed. According to the first hypothesis, most of the stable QTL found were for these compounds: lactones, esters, and monoterpenes (Figure 4). Eduardo et al. [22] also found a strong environmental effect with less than 9% of the volatiles analyzed in that case showing significant correlation between the years of evaluation.

We previously proposed that lipid-derived compounds and lactones are inversely regulated during ripening, and speculated that this could be due to a shift in fatty-acid metabolism [9]. In the present study, we identified a locus that controls the levels of some of the members of these two groups of volatiles antagonistically (i.e., with opposite additive effects). Accordingly, this locus, located at the end of LG4, co-localized with a major QTL that controls the harvest date (Figure 4). Recently, a cluster of QTL for certain esters, lactones, and other volatiles was identified in the lower half of linkage group LG4 [22], and the authors interpreted this to mean that a locus with a pleiotropic effect is responsible, since at the southern end of that chromosome a locus controlling maturity-related traits (including HD) had been identified earlier by the same research group [48]. QTL for HD had been detected in different peach mapping populations in LG1, LG2, LG3, LG4, and LG6, with those located in LG4 and LG6 having the most important effect [48-51]. Here we detected three QTL controlling HD in LG1, LG4, and LG6 of the ‘MxR_01’ map that coincide with the positions reported previously (Figure 4). Among these, the one in LG4 explained the largest percentage of the variance (50% on average across locations: EJ, AA, and IVIA) and has the largest additive effect (-23.4 days on average). Early-ripening cultivars are often a desirable objective of breeding programs, since their fruits achieve better market prices because of the “novelty” phenomenon. Since the QTL located in LG4 partially overlaps a locus controlling the production of the important fruit aromas (γ-octalactone, (E)-2-hexen-1-ol acetate and hexanal), the use of this QTL to reduce the harvest time would affect the aroma profile and vice versa. On the other hand, the QTL for HD in LG1 and LG6 (with lower effects than the previous one, 18% and 9%, respectively) did not co-localize with aroma QTL, making it more suitable for breeding for earliness without affecting quality.

Our analysis found a locus controlling the MnM trait that coincided with the localization previously reported [52]. The melting locus co-localized with flesh firmness and several volatile QTL (Figure 4). The co-localization between MnM and firmness is likely due to pleiotropic effects of the endopolygalacturonase locus [53] localized in that genomic region. Whereas the putative pleiotropic effect of this gene on volatile control is hard to explain, it is also possible that an additional linkage locus is responsible for the genetic control of the volatiles. The additive effect of these QTL suggests that selecting for non-melting flesh type in our current program would increase the levels of two lactones (γ-octalactone, 4-methyl-5-penta-1,3-dienyltetrahydrofuran-2-one) and an ester (Butyl acetate), while decreasing the levels of (E)-2-hexenal. Accordingly, non-melting and melting genotypes showed differences in these volatiles as well as in other important aroma-related compounds (Additional file 11: Table S7), resulting in all four genotypes with a lactone-rich ideotype being non-melting peaches.

The co-localization of QTL that control HD and MnM (and also firmness) with those affecting volatile production could be due to two loci with pleiotropic effects or independent linked loci. In the case of the latter scenario, increasing the number of individuals in the population mapping could improve the resolution of the QTL localization and probably unlink some of the QTL in this region and clarify if these fruit traits and volatile levels could be improved independently. Most of the market peaches for fresh consumption are melting type with the exception of those from countries such as Spain, Italy, and Mexico, where non-melting peaches are preferred [54]. The data presented in Additional file 12: Table S8 indicates that, if the ideotype pursued is an early, non-melting peach, a high number of hybrids should be developed in our breeding program in order to generate enough variability for cultivar selection.

However, the most likely explanation for the cluster of QTL identified at the bottom of LG4 is two loci with a pleiotropic effect. It is also interesting to note that a delta 9 fatty acid desaturase (ppa009359m) which we identified as a putative candidate gene for being inversely correlated to hexanal [28] co-localized with its QTL (Additional file 15: Figure S5A). Similarly, the QTL controlling (E)-2-hexen-1-ol acetate is found within the same region of the cytochrome P450 homologs (ppa006310m) which we identified as being highly correlated to this compound [28].

We identified three genomic regions that control the production of several volatiles but which do not affect the other analyzed fruit traits. A locus controlling the synthesis of 12 volatiles from C5, formed mostly by monoterpenes, was identified at the top of LG4 (Figure 4). Previously, Eduardo et al. [22] mapped in the same region a major QTL for the monoterpenes: linalool and p-menth-1-en-9-al. By analyzing the allelic variation, they also showed that two terpene synthases co-segregate with the QTL. In the current study, we analyzed both compounds, but only a stable QTL for p-menth-1-en-9-al was detected (Figure 4). Regarding the accumulation of linalool, the correlation between locations was significant, but not high (r = 0.39, Additional file 4: Table S2), indicating that environmental factors also affect the variability of this volatile and probably cause a significant QTL to only be detected at the EJ location (Additional file 5: Table S3). In fact, all the compounds of cluster C5 showed a high likelihood (LOD > 3) of association with markers at the top of LG4 in both locations (Additional file 8: Figure S3), but after permutation tests, only members of C5b (with the exception of 33) were significant in both locations (Figure 4, Additional file 5: Table S3). In addition to environment effect, the analytical variation (including e.g. matrix effect) could also contribute to lowering the QTL detection below the threshold. Concomitantly, compounds of C5a showed weak correlations between locations (r = 0.31 to r = 0.39, Additional file 4: Table S2), whereas QTL for C5b were detected in both locations. These traits also showed a higher correlation among locations (r = 0.66 to r = 0.86, Additional file 4: Table S2). In addition, the group of monoterpene-rich ideotypes showed high levels of all the compounds in C5 compared to the rest of the genotypes (Additional file 13: Table S9). Therefore, while it is possible that this locus controls the whole monoterpene module, our experiment only detected stable QTL for some of them, probably due to a sampling effect associated with the limited experiment size. In summary, our data confirms the presence of QTL for p-menth-1-en-9-al at the upper end of LG4, but also shows that this locus controls other members of the monoterpene family in peach. This locus explains between 10-40% of the volatile variance and the volatile content could be increased from 2- to 11-Fold (a = 1.0-3.5) by selecting for this locus (Additional file 5: Table S3). By analyzing the homology to 90 biochemically characterized monoterpene synthase genes described previously [55] we found a monoterpene synthase-like gene (ppa003423m), in addition to the two terpenoid synthase genes reported by Eduardo et al. [22] in the LG4 QTL genome region (data not shown). Further research is necessary to assess whether these three structural genes could account for the variation in the 12 compounds controlled by this locus (and likely all the monoterpenes), or if there are other regulatory genes (e.g., a transcription factor) that control the whole biochemical pathway. In any case, our data support the exploitation of this locus to modify the concentration of monoterpenes in fruit and also encourage further functional studies of the candidate genes located in this locus.

The volatiles γ-hexalactone and γ-octalactone have a coconut-like odor while the esters (E)-2-hexenyl acetate and ethyl acetate confer a “fruity” note to the fruit aroma [12,13]. QTL controlling these four aroma-related volatiles were discovered at the same locus at the bottom of LG6 (Figure 4). The QTL explain between 14% and 31% of the volatile variance and have additive effects of the same sign (Additional file 5: Table S3), indicating that the levels of these compounds could be improved (between 1.7- and 3.5-fold according to the additive effect) in conjunction. This source variability was not indentified previously and could be useful for volatile content manipulation. Several genes previously associated with different volatiles by a combined genomic approach [28] are localized in this region (Additional file 15: Figure S5). Among them, one protein kinase (ppa008251m) with two genes with unknown function (ppa004582m and ppa003086m) highly correlated to lactones (Additional file 15: Figure S5B). A pyruvate decarboxylase (ppa003086m) associated with ester (E)-2-hexen-1-ol acetate that we proposed as being regulated at the expression level to ensure the supply of acetyl-CoA for ester biosynthesis [28] co-localized with a stable QTL for this ester, which explains 14% of the variance in mean and has an additive effect that suggests a potential for increasing this volatile by around 3-fold (Additional file 5: Table S3, Additional file 15: Figure S5). In addition, a gene with no homolog in *Arabidopsis* (ppa002860m) that was associated with the levels of ethyl acetate [28] is also co-localized in this locus (Additional file 15: Figure S5).

Similarly, QTL with additive effects of the same sign for a lactone (4-methyl-5-penta-1,3-dienyltetrahydrofuran-2-one), an ester ((Z)-3-hexenyl acetate), and a lipid-derived compound (hexanal) were identified at the top of LG5 (Figure 4). In the case of the ester and hexanal, the QTL detected at the EJ and AA locations partially overlap and span a region of nearly 25 cM, so it remains unclear if these three QTL are controlled by the same locus or by linked loci. Since the levels of volatiles in the group of lipid-derived compounds are inversely correlated with lactones and linear esters (Figure 3), we would expect the opposite effect if the same locus controlled their production. Therefore, it is likely that these two QTL are controlled by independent linked loci. According to this scenario, the genome position of a protein kinase (ppa006108m) associated with lactones and ester [28] overlaps with the position of those QTL. The co-localization of QTL with the position of the candidate genes previously identified by a genomic approach does not prove in any way a cause-effect relationship. QTL positions estimated by a low-resolution map span over several hundreds and even thousands of genes in addition to those that are candidates (not to mention other regulatory elements like microRNAs that could explain the phenotypic variance). Moreover, several of the candidate genes indentified previously for being associated with a given volatile, here failed to co-localize with the QTL controlling these compounds. In addition, evidence for allelic variation within the genes involved must first be presented in order for them to become true candidates. In any case, our results provide additional genetic evidence for linking genes to traits that could be used as a starting point for these studies.

Probably as a result of the high level of homozygosity revealed by the SNP genotyping, the genetic map of ‘Granada’ had low coverage (e.g., for chromosomes 1, 2, 3, 4, 5, and 8), and, consequently, a small number of QTL were detected (Figure 5, Additional file 6: Table S4, Additional file 7: Table S5). Only two QTL that were stable among locations, one for a monoterpene (43) and the other for fruit weight, were identified in LG2 and LG6, respectively (Figure 5). A minor QTL for peach weight had previously been identified in another locus in LG6 [48], indicating that the one found here represents a novel source of variability. The QTL for fruit weight identified here also has a minor effect (r^2^ = 0.15 in mean), and the additive effect is 22 g, but since its localization does not overlap with QTL for volatiles, it should be possible to use it to increase fruit size to some extent without modifying the aroma profile of the fruit.

## Conclusion

The results presented here confirmed previously identified loci and also discovered novel loci for important aroma-related volatiles in peach. Furthermore, our results are in agreement with the modularity of the genetic control of volatile production in peach, suggesting that groups of related volatiles rather than single volatiles could be the target of aroma improvement. The source of variability described here could be used in the quality improvement of peach and could also aid in the discovery of genes controlling the aroma of peach fruit.

## Competing interests

The authors declare that they have no competing interests.

## Authors’ contributions

GS conceived and designed the work, performed the metabolomics and fruit quality analyses, analyzed the data, and wrote the manuscript. JM harvested and performed the fruit quality analysis. JR and JG harvested the fruit. AM contributed with the QTL analysis and the overall discussion of the results. MLB developed the population mapping and conceived the work. AG conceived, designed, and supervised the work. All authors read and approved the final manuscript.

## Supplementary Material

Additional file 1: Table S1 Genotyping data set. For each SNP, the name and the position (in bp) at the chromosome (Chr) are shown. Missing values are indicated with “-- “. Click here for file

Additional file 2: Figure S1 SNPs selected for Sc1 of ‘MxR_01’. A) Linkage group obtained with all the polymorphic SNPs mapped to scaffold 1 for ‘MxR_01’ (265 markers). B) The map obtained after selecting unique, informative SNPs for each map position (26 markers). For each map, the SNP positions in cM are given at the left of each. SNP names are indicated using the first 3 characters of the scaffold that the marker was mapped to (e.g., Sc1 indicates Scaffold 1). The relative position in the genome of each SNP is indicated with the last number (e.g., 1129 for Sc1_SNP_IGA_1129). The exact genome position can be found at the genome browser (http://www.rosaceae.org/gb/gbrowse/prunus_persica/). Click here for file

Additional file 3: Figure S2 Fruit variability within the population mapping from the “El Jimeno” trial. Four representative fruits for each breeding line and parental genotypes are shown. In each photo the number (for breeding line) or name (for parental) of the genotype is indicated. The bar at the left bottom corner indicates a 1-cm scale. Click here for file

Additional file 4: Table S2 Volatiles analyzed in this study. For each volatile, the cluster (C1-C12) where the compound was found in the HCA (Figure 2) is shown. Cluster 5 is divided into three sub-clusters indicated with the letters a, b, and c. The volatile number (Nº) indicates the compound position in the HCA. For each compound, the cas number and an identification code (id) is given that is formed by the ion used for quantification and the retention time (given in scan number) where the peak was found. Compounds identified by comparing their retention time to authentic standards are highlighted in bold letters. n.a. = not assigned. Family indicates the biosynthetic origin or chemical nature of the volatile. un. = unknown. The Pearson correlation coefficients of volatile levels between the EJ and AA locations are indicated (corr_EJ-AA). * and ** indicate that the correlation is significant at α = 0.05 and α = 0.01, respectively. Shaded correlation coefficients indicate that stable QTL for those volatiles were found. Click here for file

Additional file 5: Table S3 Volatile QTL detected for the ‘MxR_01’ map. For each QTL, the annotation of the volatile it controls (Nº, Name, Cluster according to Figure 2 and Family), the location (EJ or AA), the linkage group (LG), the position in cM (Position), the likelihood of odds (LOD), the additive effect (Additive), the proportion of the phenotypic variance explained (R2) and the 2-LOD confidence interval are shown. All the QTL shown are significant as assessed by a 1000-permutation test at α = 0.05. Click here for file

Additional file 6: Table S4 Volatile QTL detected for ‘Granada’ map. For each QTL, the annotation of the volatile it controls (Nº, Name, Cluster according to Figure 2 and Family), the location (EJ or AA), the linkage group (LG), the position in cM (Position), the likelihood of odds (LOD), the additive effect (Additive), the proportion of the phenotypic variance explained (R2) and the 2-LOD confidence interval are shown. All the QTL shown are significant as assessed by a 1000-permutation test at α = 0.05. Click here for file

Additional file 7: Table S5 QTL for fruit type and maturity-related traits. For each QTL, the location (EJ, AA or IVIA), the linkage group (LG), the position in cM (Position), the likelihood of odds (LOD), the additive effect (Additive), the proportion of the phenotypic variance explained (R2), and 2-LOD confidence interval are shown. All the QTL shown are significant as assessed by a 1000-permutation test at α = 0.05. The traits analyzed are: melting/non-melting fruit type (MnM), flesh firmness (Firmness), fruit weight (Weight), solid soluble content (SSC), peel ground color parameters (L, lightness; C, chroma; and H, color measured in Hue degrees), and harvest date (HD). The QTL detected in the ‘MxR_01’ and ‘Granada’ maps are listed at the top and the bottom of the table, respectively. Click here for file

Additional file 10: Table S6 Phenotyping data set. The data for all the traits analyzed are shown. For each trait, the location “El Jimeneo” (EJ), “Aguas Amargas” (AA), and IVIA is indicated. The volatile compounds are codified with the id given in Additional file 4: Table S2. Missing values are indicated with “-- “. Click here for file

Additional file 11: Table S7 Difference in volatile levels between non-melting and melting peaches. The differences in volatile levels were stated by ANOVA analysis; the p- value (p) obtained for each volatile is shown. nM/M indicates the fold change of volatile levels between non-melting and melting genotypes. Click here for file

Additional file 12: Table S8 Percentage of melting/non-melting peaches in early, medium and late genotypes. Click here for file

Additional file 13: Table S9 Difference in volatile levels between monoterpene-rich ideotype and the rest of the genotype. The differences were stated by ANOVA analysis, the p- value (p) obtained for each volatile is shown. Monoterpene-rich indicates the fold change of volatile levels between the genotypes with monoterpene-rich ideotypes and the rest of the genotypes. Click here for file

Additional file 8: Figure S3 Heatmap of LOD scores from volatile QTL analysis for ‘MxR_01’ at the EJ (top) and AA (bottom) locations. The LOD score (computed by single correlation analysis) for each marker/volatile pair is presented in a different color according to their additive effects (a), red for negative a and blue for positive a. The color intensity is according to the LOD value, the higher the intensity the higher the LOD score. For each linkage group (LG1, LG3-LG8) the markers are ordered from left to right according to the position in the peach genome. The volatiles are located on the right, ordered according to the position on the HCA of Figure 2. C1-C12 indicates the volatile clusters. Vertical and horizontal lines divide the linkage groups and the volatile clusters, respectively. EJ and AA indicate the locations of “El Jimeneo” and “Aguas Amargas”, respectively. Click here for file

Additional file 9: Figure S4 Heatmap of LOD scores from volatile QTL analysis for ‘Granada’ at the EJ (top) and AA (bottom) locations. The LOD score (computed by single correlation analysis) for each marker/volatile pair is presented in a different color according to their additive effects (a), red for negatives a and blue for positive a. The color intensity is according to the LOD value, the higher the intensity the higher the LOD score. For each linkage group (LG1-LG2, LG4-LG8) the markers are ordered from left to right according to the position in the peach genome. The volatiles are ordered according to the position on the HCA of Figure 2. C1-C12 indicates the volatile clusters. Vertical and horizontal lines divide the linkage groups and the volatile clusters, respectively. EJ and AA indicate the locations of “El Jimeneo” and “Aguas Amargas”, respectively. Click here for file

Additional file 14: Table S10 Difference in volatile levels between lactone-rich ideotype and the rest of the genotypes. The differences were stated by ANOVA analysis, the p- value (p) obtained for each volatile is shown. Lactone-rich indicates the fold change of volatile levels between the genotypes with lactone-rich ideotypes and the rest of the genotypes. Click here for file

Additional file 15: Figure S5 Co-localization of volatile QTL with candidate genes identified previously. Physical (left) and linkage (right) maps of chromosomes where volatile QTL were indentified are shown. The QTL are colored according to the direction of the additive (a) effect (blue for positive and red for negative). Bars and lines represent 1-LOD and 2-LOD support intervals. The candidate genes previously associated with different volatile groups [28] are indicated with a different color. The position of SNPs and candidate genes in the scaffolds of the peach genome v1 is indicated at the left of the map in arbitrary units (map position in base pair/ 5×10^5^). SNP positions in the linkage map are indicated at the right of the map in cM. A) QTL for LG4 of ‘MxR’ and the corresponding scaffold are shown. B) QTL for LG5 and LG6 of ‘MxR’ and the corresponding scaffolds are shown. C) QTL for LG2 of ‘Granada’ and the corresponding scaffold are shown. Click here for file
