# Supplementary figures and images for: The peach volatilome modularity is reflected at the genetic and environmental response levels in a QTL mapping population
Source: BMC Plant Biol. 2014 May 19;14:137. doi: 10.1186/1471-2229-14-137 (PMC4067740; doi:10.1186/1471-2229-14-137)

## Slide 1
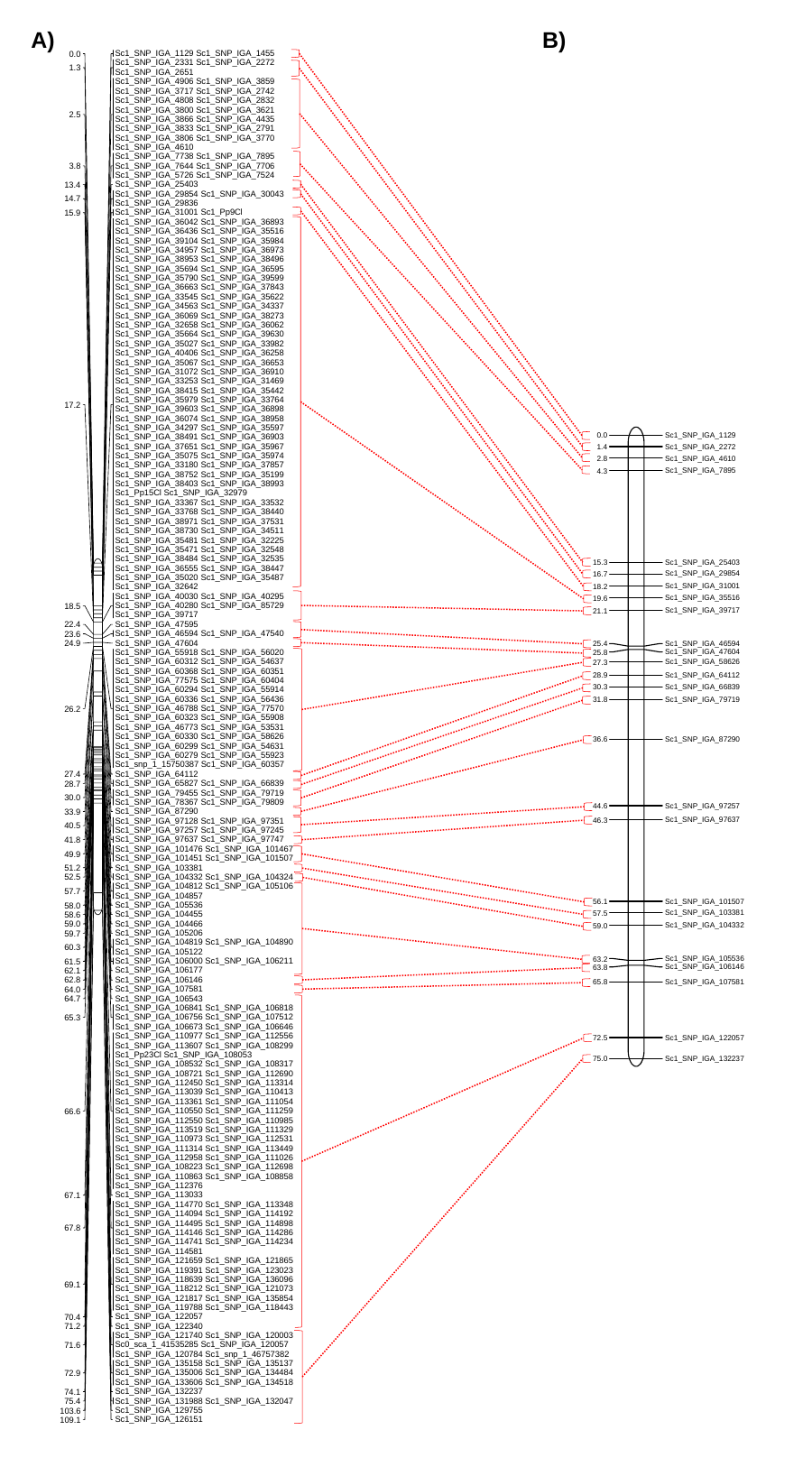

A)
B)

Supplement: Additional file 1: Table S1 — Genotyping data set. For each SNP, the name and the position (in bp) at the chromosome (Chr) are shown. Missing values are indicated with “-- “. [file 1471-2229-14-137-S1.pptx]

01

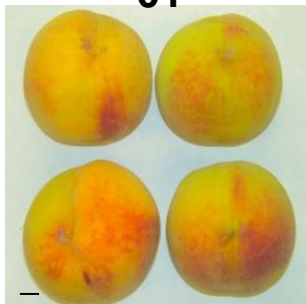

02

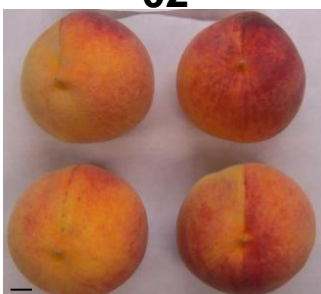

03

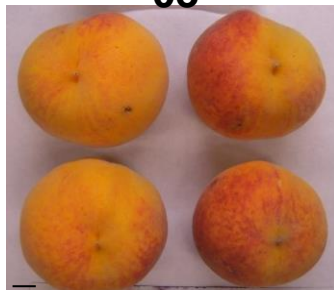

07

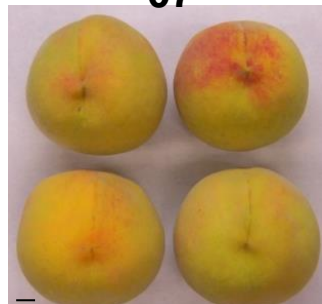

09

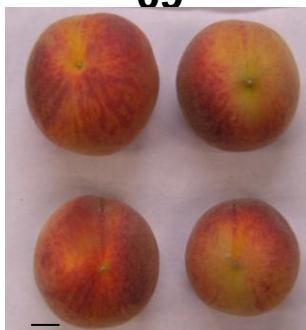

11

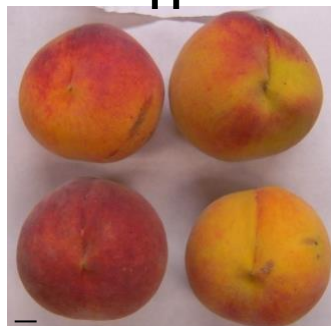

12

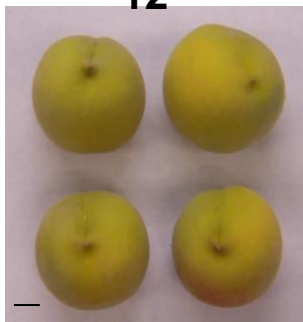

16

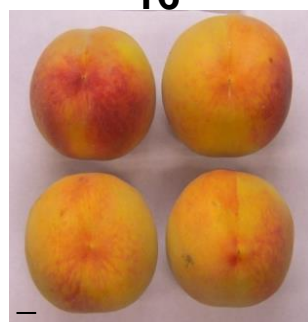

17

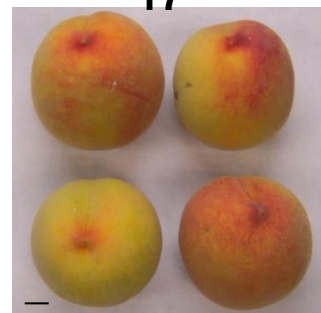

18

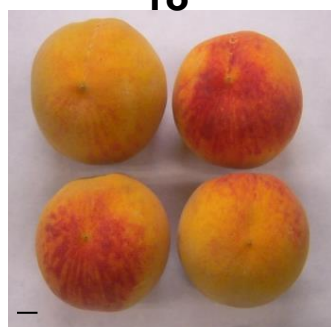

19

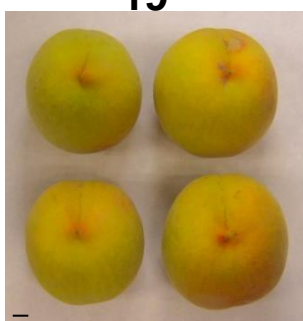

20

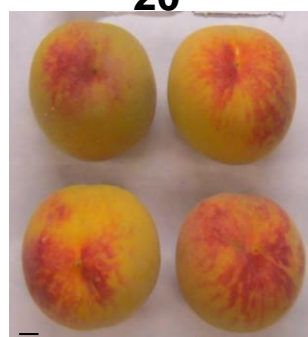

21

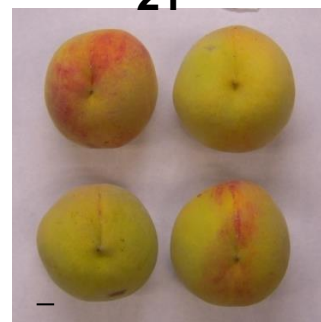

22

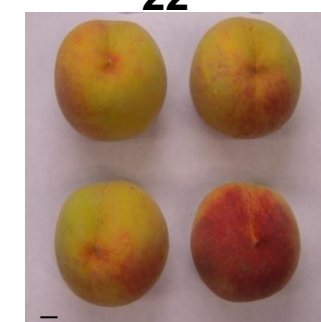

24

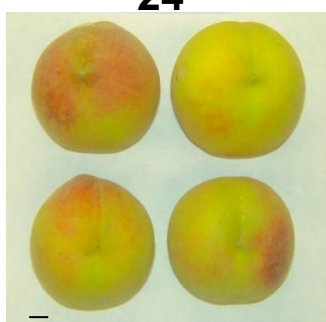

28

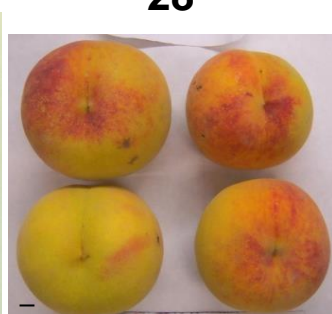

30

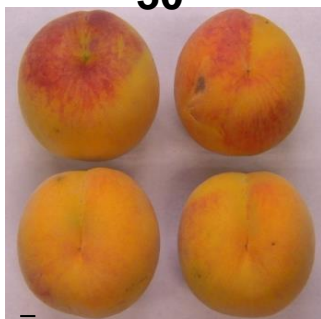

31

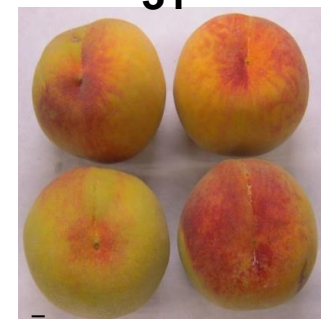

33

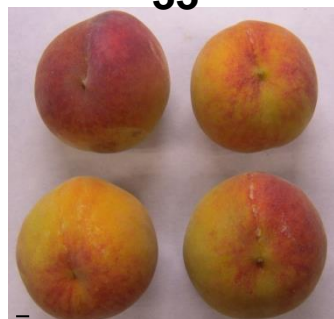

35

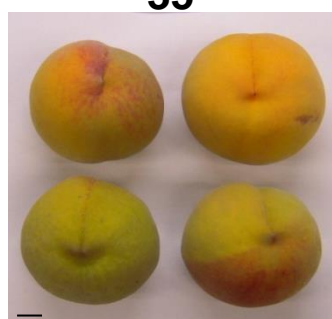

36

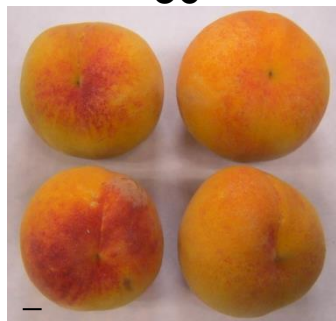

37

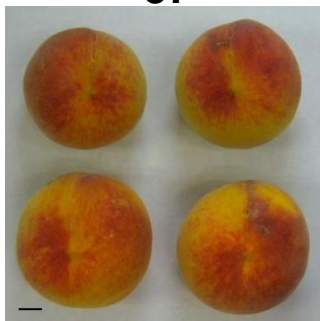

38

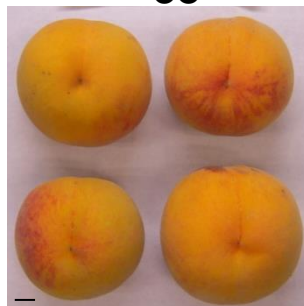

39

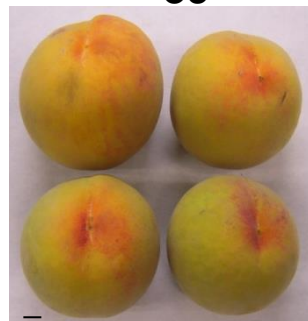

41

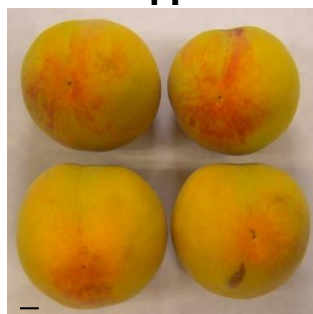

42

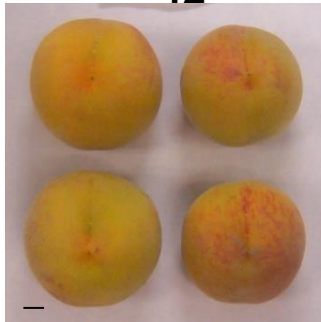

43

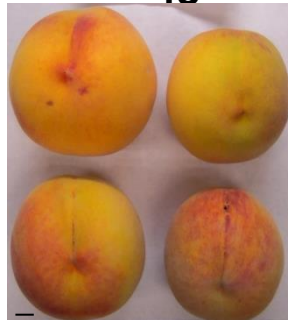

44

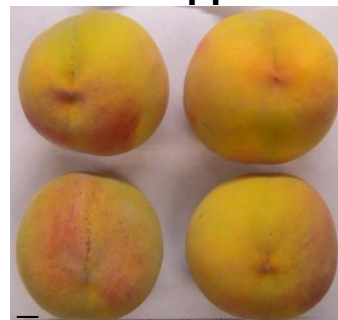

45

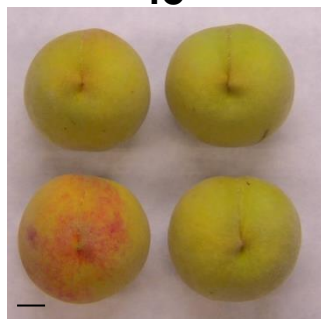

46

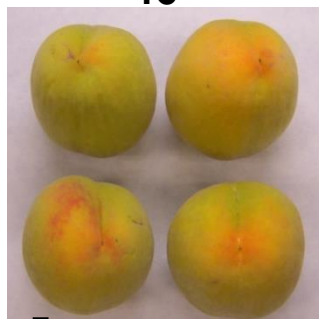

48

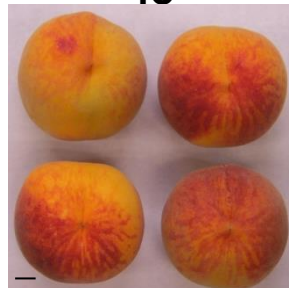

49

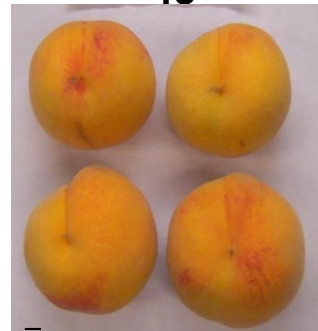

50

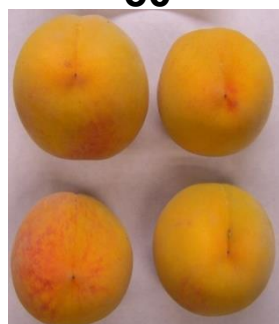

52

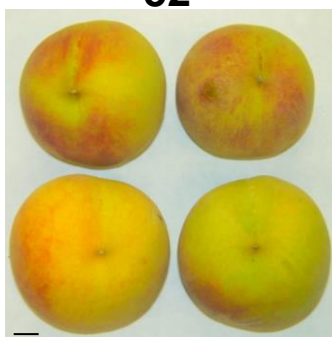

53

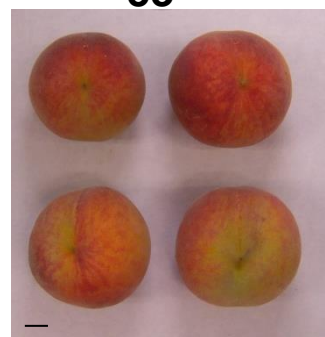

54

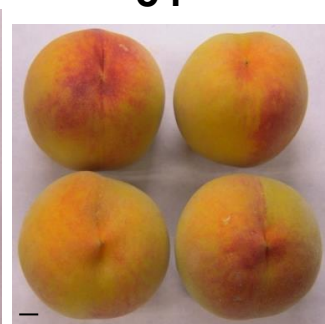

55

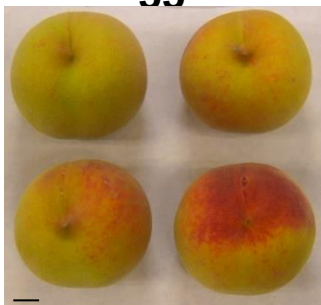

57

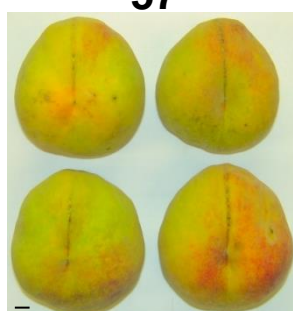

58

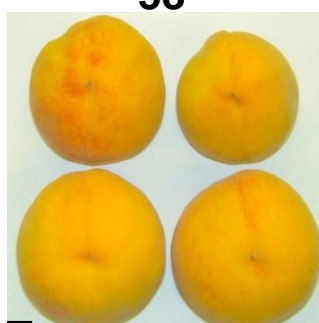

60

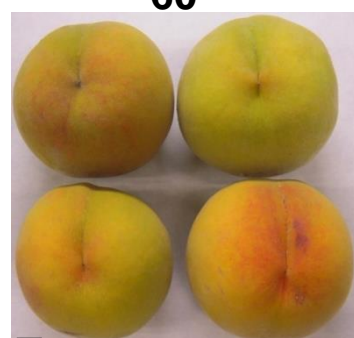

61

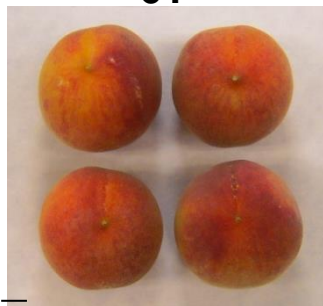

62

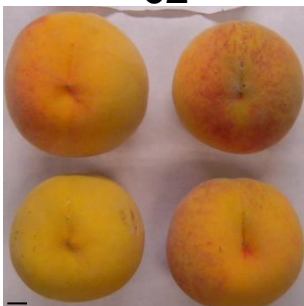

63

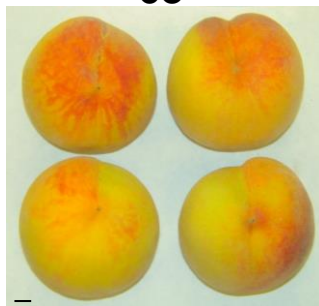

64

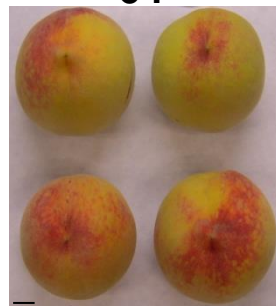

66

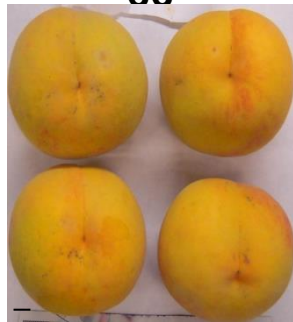

67

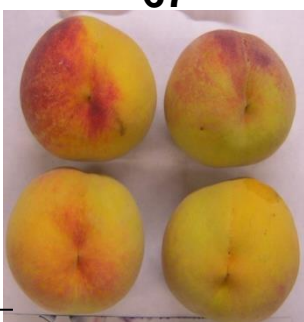

68

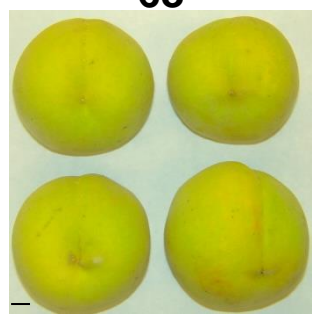

69

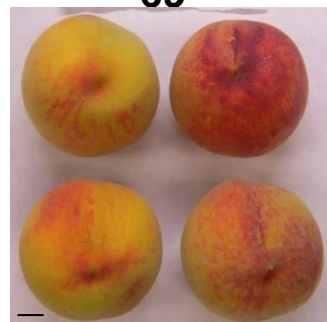

71

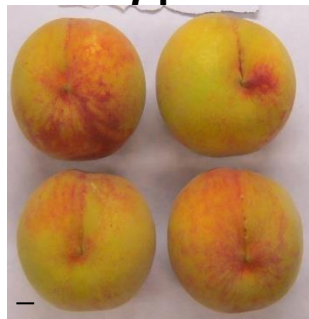

74

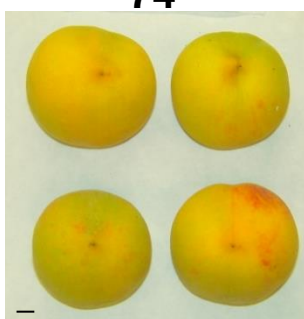

75

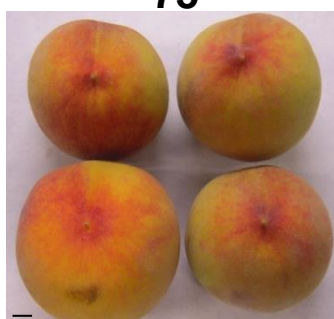

77

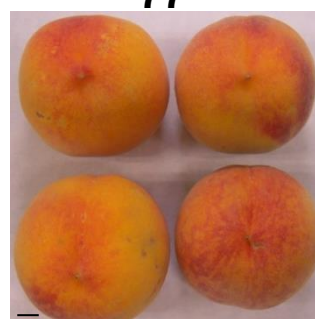

78

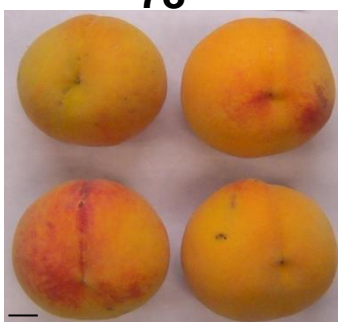

79

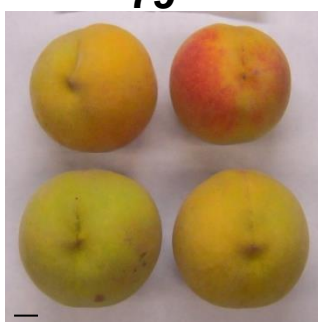

80

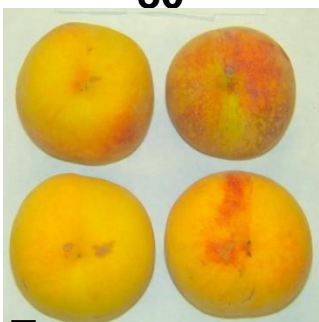

82

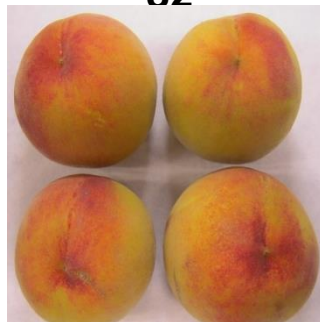

83

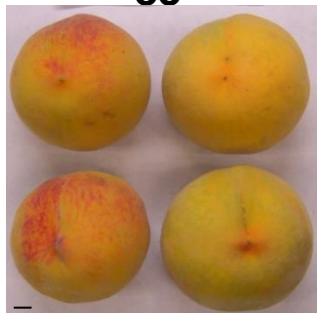

85

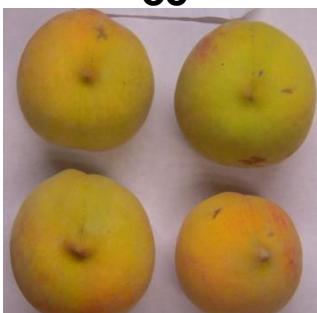

86

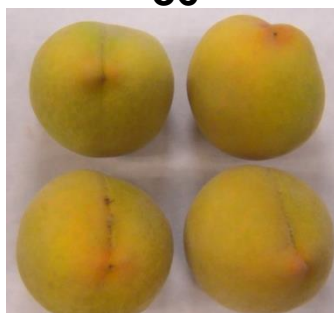

88

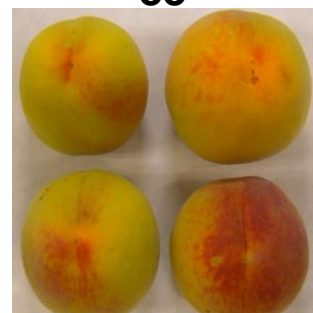

90

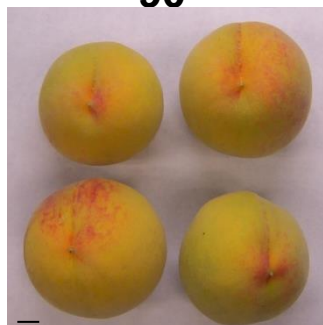

91

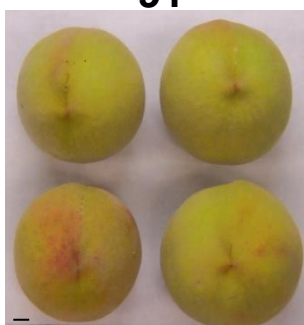

92

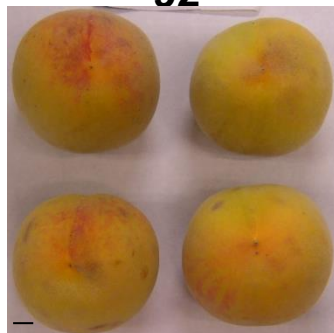

93

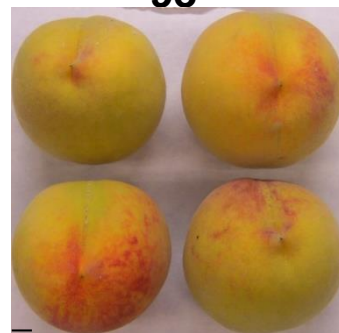

94

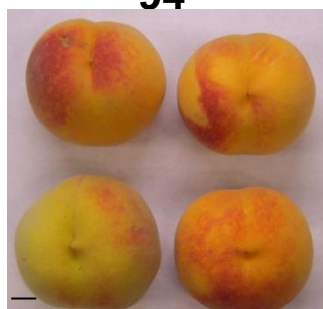

96

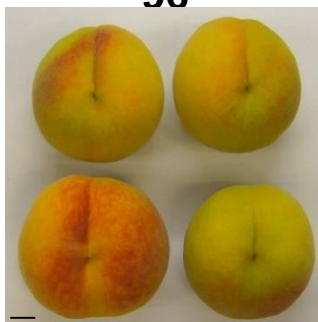

97

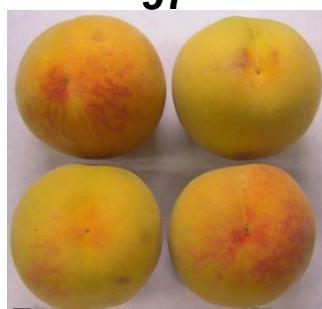

100

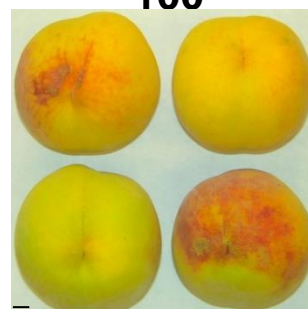

102

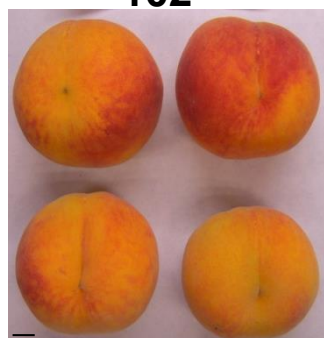

'MxR 01'

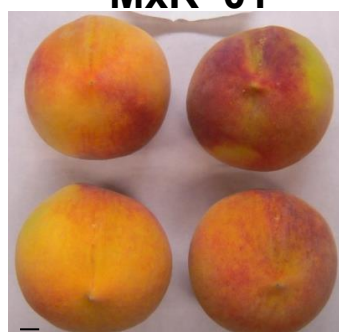

'Granada'

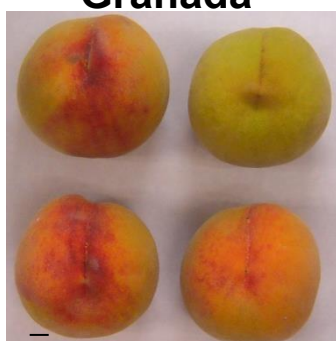

Supplement: Additional file 2: Figure S1 — SNPs selected for Sc1 of ‘MxR_01’. A) Linkage group obtained with all the polymorphic SNPs mapped to scaffold 1 for ‘MxR_01’ (265 markers). B) The map obtained after selecting unique, informative SNPs for each map position (26 markers). For each map, the SNP positions in cM are given at the left of each. SNP names are indicated using the first 3 characters of the scaffold that the marker was mapped to (e.g., Sc1 indicates Scaffold 1). The relative position in the genome of each SNP is indicated with the last number (e.g., 1129 for Sc1_SNP_IGA_1129). The exact genome position can be found at the genome browser (http://www.rosaceae.org/gb/gbrowse/prunus_persica/). [file 1471-2229-14-137-S2.pdf]

## Slide 1
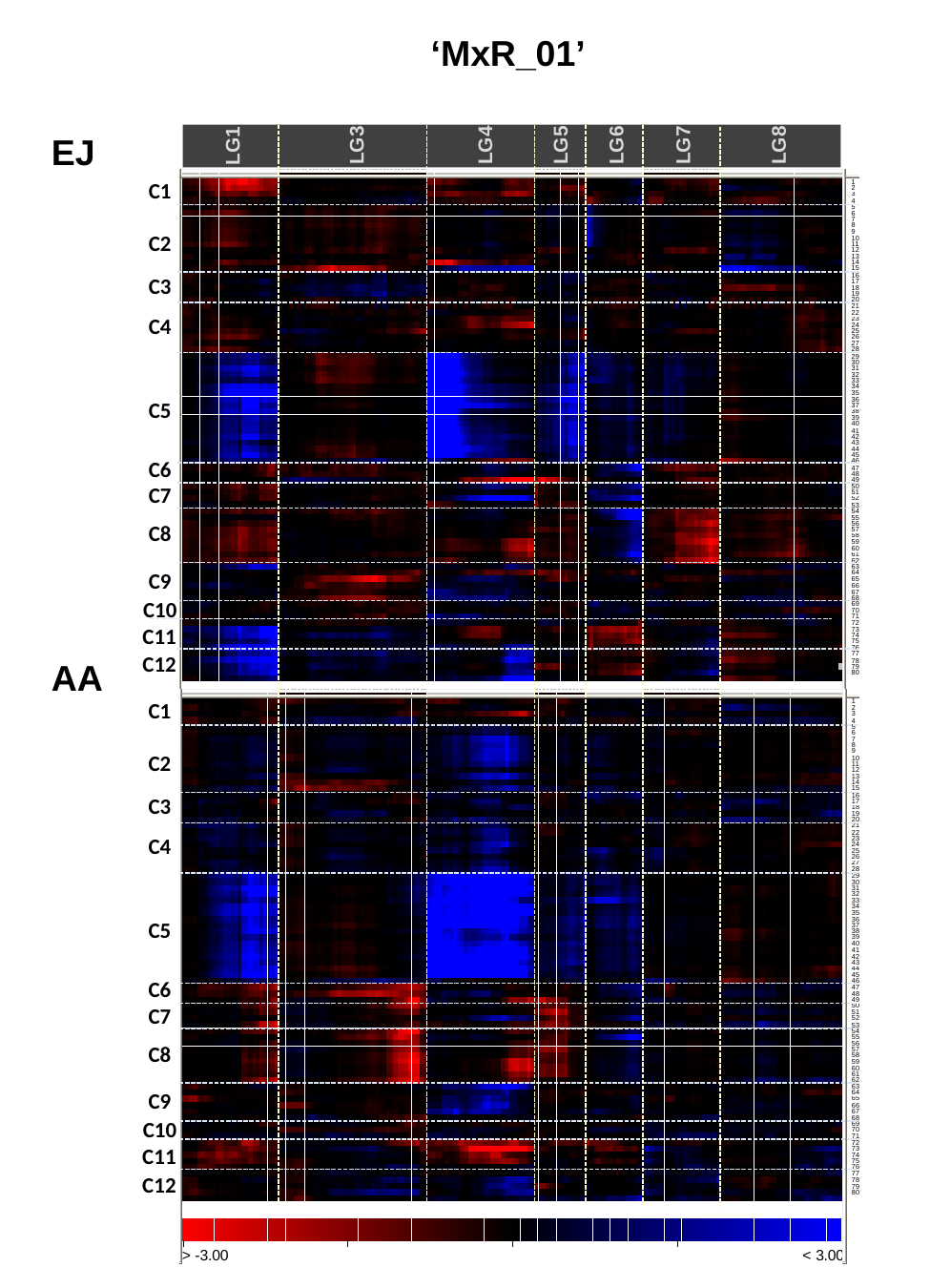

‘MxR_01’
EJ
LG3
LG4
LG5
LG6
LG7
LG8
LG1
C1
C2
C3
C4
C5
C6
C7
C8
C9
C10
C11
C12
AA
C1
C2
C3
C4
C5
C6
C7
C8
C9
C10
C11
C12

Supplement: Additional file 7: Table S5 — QTL for fruit type and maturity-related traits. For each QTL, the location (EJ, AA or IVIA), the linkage group (LG), the position in cM (Position), the likelihood of odds (LOD), the additive effect (Additive), the proportion of the phenotypic variance explained (R2), and 2-LOD confidence interval are shown. All the QTL shown are significant as assessed by a 1000-permutation test at α = 0.05. The traits analyzed are: melting/non-melting fruit type (MnM), flesh firmness (Firmness), fruit weight (Weight), solid soluble content (SSC), peel ground color parameters (L, lightness; C, chroma; and H, color measured in Hue degrees), and harvest date (HD). The QTL detected in the ‘MxR_01’ and ‘Granada’ maps are listed at the top and the bottom of the table, respectively. [file 1471-2229-14-137-S7.pptx]

‘Granada’

EJ

AA

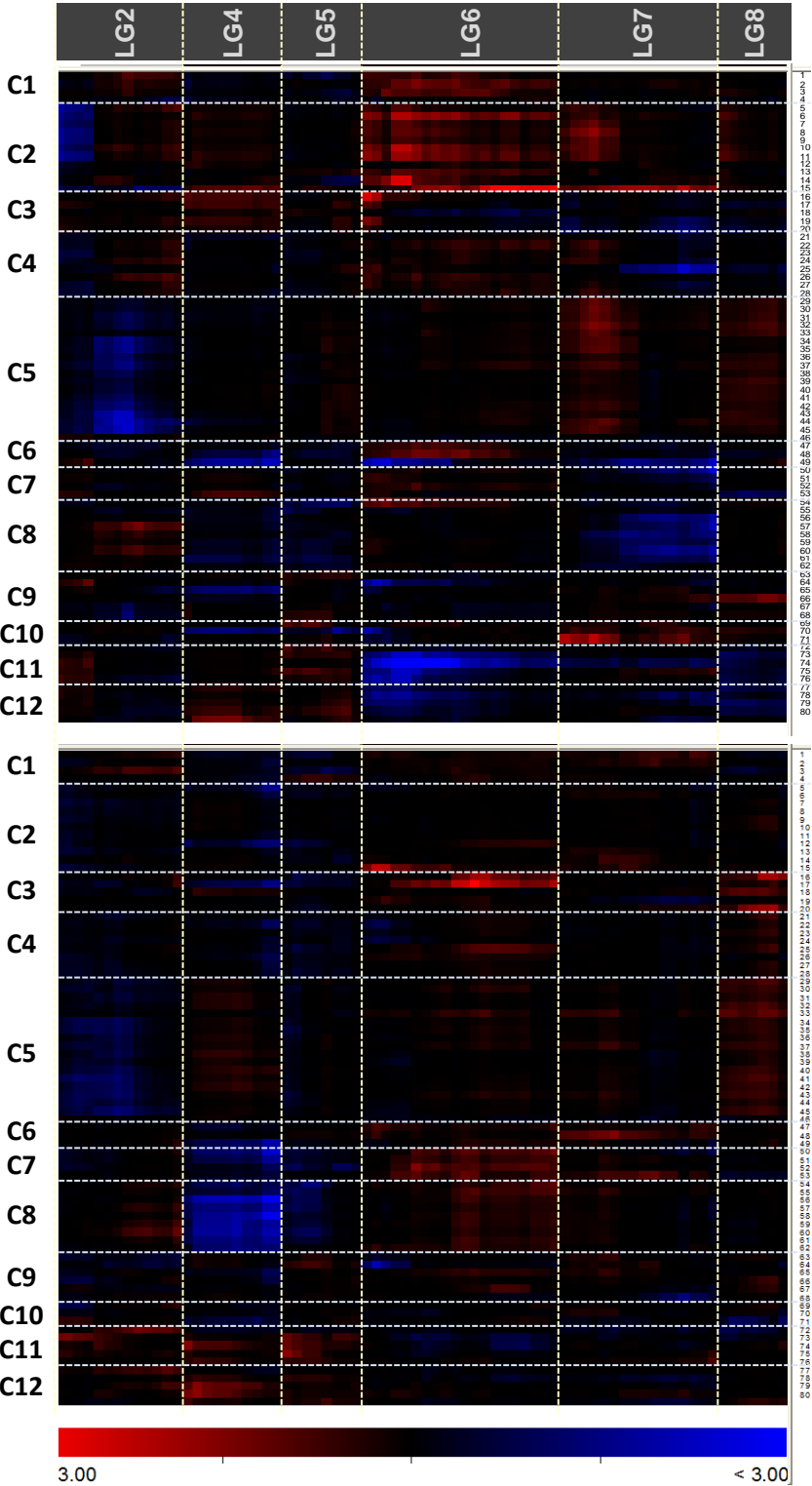

Supplement: Additional file 8: Figure S3 — Heatmap of LOD scores from volatile QTL analysis for ‘MxR_01’ at the EJ (top) and AA (bottom) locations. The LOD score (computed by single correlation analysis) for each marker/volatile pair is presented in a different color according to their additive effects (a), red for negative a and blue for positive a. The color intensity is according to the LOD value, the higher the intensity the higher the LOD score. For each linkage group (LG1, LG3-LG8) the markers are ordered from left to right according to the position in the peach genome. The volatiles are located on the right, ordered according to the position on the HCA of Figure 2. C1-C12 indicates the volatile clusters. Vertical and horizontal lines divide the linkage groups and the volatile clusters, respectively. EJ and AA indicate the locations of “El Jimeneo” and “Aguas Amargas”, respectively. [file 1471-2229-14-137-S8.pdf]
