# Supplementary material for: The peach volatilome modularity is reflected at the genetic and environmental response levels in a QTL mapping population
Source: BMC Plant Biol. 2014 May 19;14:137. doi: 10.1186/1471-2229-14-137 (PMC4067740; doi:10.1186/1471-2229-14-137)
Supplement: Additional file 3: Figure S2 — Fruit variability within the population mapping from the “El Jimeno” trial. Four representative fruits for each breeding line and parental genotypes are shown. In each photo the number (for breeding line) or name (for parental) of the genotype is indicated. The bar at the left bottom corner indicates a 1-cm scale. [file 1471-2229-14-137-S3.pdf]

Nº	CAS N°	id
1	123-86-4	43_7136
2	19550-08-4	73_9306
3	75-98-9	41_6638
4	111-14-8	73_11030
5	n.a.	165_16433
6	110-42-9	74_14613
7	693-54-9	58_12978
8	1534-27-6	72_12911
9	111-11-5	74_11980
10	103-09-3	70_12284
11	1731-84-6	74_13339
12	111-82-0	74_16928
13	103-11-7	70_13492
14	42928-87-0	55_13398
15	122-78-1	91_11117
16	149-57-5	73_11681
17	124-07-2	73_12448
18	142-62-1	60_9627
19	112-05-0	73_13753
20	334-48-5	73_14964
21	54515-76-3	172_17688
22	93-16-3	178_15640
23	119-61-9	105_18590
24	643-65-2	196_20012
25	140-11-4	108_12704
26	464-49-3	95_12787
27	19219-84-2	105_14972
28	118-61-6	120_14256
29	586-62-9	121_11693
30	98-55-5	121_13280
31	7392-19-0	139_9925
32	78-70-6	93_11733
33	5989-27-5	93_10867
34	121974	59_11450
35	132130	59_11676
36	122-00-9	119_13188
37	70286-20-3	138_12371
38	27831-13-6	117_11758
39	1197-01-9	43_13124
40	432-25-7	123_10728
41	21195-59-5	134_12449
42	70786-44-6	137_13215
43	29548-14-9	94_13611
44	1197-06-4	69_13073
45	29957-43-5	71_11820
46	99-87-6	119_10786
47	141-78-6	43_3992
48	64-17-5	45_2418
49	n.a.	68_15955

**50**	142-92-7	43_10318
**51**	3681-71-8	67_10239
**52**	2497-18-9	43_10351
**53**	10437-78-2	80_10583
**54**	110-19-0	43_6475
**55**	695-06-7	85_11181
**56**	27593-23-3	95_16515
**57**	705-86-2	99_16910
**58**	706-14-9	85_16556
**59**	104-50-7	85_14019
**60**	104-61-0	85_15326
**61**	93787-95-2	68_16405
**62**	105-21-5	85_12613
**63**	79-77-6	177_16736
**64**	23726-93-4	69_15595
**65**	110-93-0	108_9983
**66**	689-67-8	43_16192
**67**	143-08-8	56_12707
**68**	53535-33-4	56_9747
**69**	64-19-7	45_3430
**70**	104-76-7	57_10633
**71**	111-87-5	56_11249
**72**	616-25-1	57_5029
**73**	6789-80-6	69_6953
**74**	3208-16-0	81_5323
**75**	1629-58-9	55_5080
**76**	4313-03-5	81_10494
**77**	110-62-3	44_5291
**78**	3777-69-3	81_10119
**79**	66-25-1	56_6998
**80**	6728-26-3	83_7941
**81**	97-53-0	164_15243

Name	Cluster
Butyl acetate	C1
3,4-Dimethyl-3-hexanol	C1
2,2-Dimethylpropanoic acid	C1
**Heptanoic acid**	C1
8,8,9-Trimethyl-deca-3,5-diene-2,7-dione	C2
Methyl decanoate	C2
2-Decanone	C2
3-Decanone	C2
Methyl octanoate	C2
2-Ethyl-1-hexanol acetate	C2
Methyl nonanoate	C2
Methyl dodecanoate	C2
2-Ethylhexyl 2-propenoate	C2
4-(Prop-2-enoyloxy)octane	C2
**Benzeneacetaldehyde**	C2
**Ethylhexanoic acid**	C3
**Octanoic Acid**	C3
**Hexanoic acid**	C3
**Nonanoic acid**	C3
Decanoic acid	C3
2,7-Ethanonaphth[2,3-b]oxirene, 1a,2,7,7a-tetrahydro-, (1aa,2a,7a,7aa)-	C4
Isoeugenol methyl ether	C4
**Benzophenone**	C4
3-Methylbenzophenone	C4
Benzyl acetate	C4
Alcanfor	C4
(1,3-dimethylbutyl)-benzene	C4
Ethyl salicylate	C4
**Terpinolene**	C5a
**α-Terpinol**	C5a
2H-Pyran, 2-ethenyltetrahydro-2,6,6-trimethyl-	C5a
**Linalool**	C5a
**δ-Limonene**	C5b
cis **-Linaloloxide**	C5b
α-Methyl-α-[4-methyl-3-pentenyl]oxiranemethanol	C5b
4-Methylacetophenone	C5b
4-Acetyl-1-methylcyclohexene	C5b
3,4-Dimethylstyrene	C5b
p-Cymen-8-ol	C5b
β-Cyclocitral	C5b
1,3,8-p-Menthatriene	C5b
3,6-Dimethyl-2,3,3a,4,5,7a-hexahydrobenzofuran	C5b
p-Menth-1-en-9-al	C5b
cis-Carveol	C5b
3,7-Dimethyl-1,5,7-octatrien-3-ol	C5b
p **-Cymene**	C5c
**Ethyl Acetate**	C6
**Ethanol**	C6
4-Methyl-5-penta-1,3-dienyltetrahydrofuran-2-one	C6

**Hexyl acetate**	C7
**(Z)-3-Hexenyl acetate**	C7
**(E)-2-Hexenyl acetate**	C7
3-Cyclohexenyl acetate	C7
**2-Methylpropyl acetate**	C8
**γ-Hexalactone**	C8
**6-Pentyl-2H-pyran-2-one**	C8
**δ-Decalactone**	C8
**γ-Decalactone**	C8
**γ-Octalactone**	C8
γ-Nonalactone	C8
**γ-Jasmolactone**	C8
**γ-Heptalactone**	C8
**β-Ionone**	C9
**β-Damascenone**	C9
**6-methyl-5-hepten-2-one**	C9
**Geranyl acetone**	C9
**Nonalol**	C9
**Heptanol**	C9
Acetic acid	C10
2-Ethyl-1-hexanol	C10
**1-Octanol**	C10
**1-Penten-3-ol**	C11
**(Z)-3-Hexenal**	C11
**2-Ethylfuran**	C11
**1-Penten-3-one**	C11
**(E,E)-2,4-Heptadienal**	C11
**Pentanal**	C12
**2-pentyl-furan**	C12
**Hexanal**	C12
(E)-2-Hexenal	C12
**Eugenol**	C12

Family	corr_EJ-AA
Ester	0.34**
Alcohol	0.25*
Carboxilic acid	0.10
Carboxilic acid	0.21
Long Ketone/Ester	0.02
Long Ketone/Ester	0.17
Long Ketone/Ester	0.11
Long Ketone/Ester	0.11
Long Ketone/Ester	0.18
Long Ketone/Ester	0.08
Long Ketone/Ester	0.09
Long Ketone/Ester	0.07
Long Ketone/Ester	-0.03
Carboxilic acid	-0.03
Aromatic	0.25*
Carboxilic acid	-0.04
Carboxilic acid	-0.05
Carboxilic acid	0.07
Carboxilic acid	0.14
Carboxilic acid	-0.02
un.	0.06
Aromatic Ether	0.17
Aromatic Ketone	0.10
Aromatic Ketone	-0.06
Aromatic Ester	0.13
Ketone	0.20
Aromatic	0.21
Aromatic Ester	0.23
Monoterpene	0.31*
Monoterpene	0.31*
Monoterpene	0.39**
Monoterpene	0.39**
Monoterpene	0.33*
Monoterpene	0.73*
un.	0.67*
un.	0.81*
Monoterpene	0.87*
un	0.77*
un.	0.75*
Monoterpene	0.86*
Monoterpene	0.72*
un.	0.84*
Monoterpene	0.77*
un.	0.66*
un.	0.68*
Monoterpene	0.76*
Ester	0.35*
Alcohol	0.22*
Lactone	0.48*

Ester	0.27**
Ester	0.18
Ester	0.40**
Ester	0.06
Ester	0.36**
Lactone	0.50**
Lactone	0.27*
Lactone	0.36**
Lactone	0.14*
Lactone	0.33*
Lactone	0.33*
Lactone	0.39**
Lactone	0.28*
Carotenoid-derived	0.14
Carotenoid-derived	0.30*
Carotenoid-derived	0.27*
Carotenoid-derived	0.20
Alcohol	0.12
Alcohol	-0.01
Carboxilic acid	0.33*
Alcohol	0.09
Alcohol	0.01
Lipid-derived	-0.14
Lipid-derived	0.22
Lipid-derived	0.18
Lipid-derived	0.19
Lipid-derived	0.22
Lipid-derived	0.06
Lipid-derived	0.57**
Lipid-derived	0.25*
Lipid-derived	0.24
Aromatic alcohol	0.46**
