# Supplementary material for: The peach volatilome modularity is reflected at the genetic and environmental response levels in a QTL mapping population
Source: BMC Plant Biol. 2014 May 19;14:137. doi: 10.1186/1471-2229-14-137 (PMC4067740; doi:10.1186/1471-2229-14-137)
Supplement: Additional file 13: Table S9 — Difference in volatile levels between monoterpene-rich ideotype and the rest of the genotype. The differences were stated by ANOVA analysis, the p- value (p) obtained for each volatile is shown. Monoterpene-rich indicates the fold change of volatile levels between the genotypes with monoterpene-rich ideotypes and the rest of the genotypes. [file 1471-2229-14-137-S13.pptx]

## Slide 1
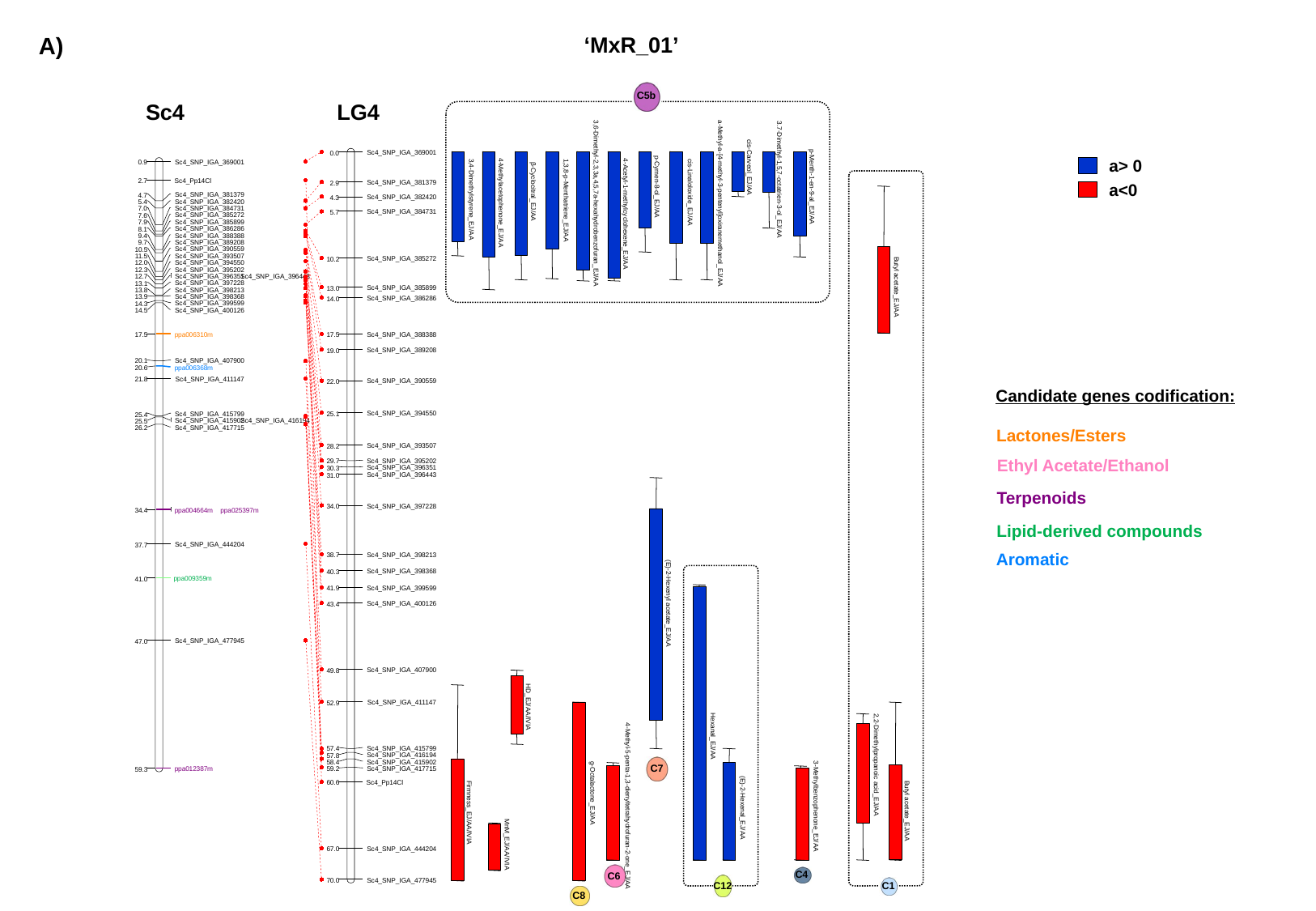

A)
‘MxR_01’
C5b
3.7-Dimethyl-1,5,7-octatrien-3-ol_EJ/AA
p-Menth-1-en-9-al_EJ/AA
p-Cymen-8-ol_EJ/AA
β-Cyclocitral_EJ/AA
cis-Linaloloxide_EJ/AA
1,3,8-p-Menthatriene_EJ/AA
3,4-Dimethylstyrene_EJ/AA
3,6-Dimethyl-2,3,3a,4,5,7a-hexahydrobenzofuran_EJ/AA
4-Acetyl-1-methylcyclohexene_EJ/AA
4-Methylacetophenone_EJ/AA
a-Methyl-a-[4-methyl-3-pentenyl]oxiranemethanol_EJ/AA
cis-Carveol_EJ/AA
Sc4
LG4
Sc4_SNP_IGA_369001
0.0
Sc4_SNP_IGA_381379
2.9
Sc4_SNP_IGA_382420
4.3
Sc4_SNP_IGA_384731
5.7
Sc4_SNP_IGA_385272
10.2
Sc4_SNP_IGA_385899
13.0
Sc4_SNP_IGA_386286
14.0
Sc4_SNP_IGA_388388
17.5
Sc4_SNP_IGA_389208
19.0
Sc4_SNP_IGA_390559
22.0
Sc4_SNP_IGA_394550
25.1
Sc4_SNP_IGA_393507
28.2
Sc4_SNP_IGA_395202
29.7
Sc4_SNP_IGA_396351
30.3
Sc4_SNP_IGA_396443
31.0
Sc4_SNP_IGA_397228
34.0
Sc4_SNP_IGA_398213
38.7
Sc4_SNP_IGA_398368
40.3
Sc4_SNP_IGA_399599
41.9
Sc4_SNP_IGA_400126
43.4
Sc4_SNP_IGA_407900
49.8
Sc4_SNP_IGA_411147
52.9
Sc4_SNP_IGA_415799
57.4
Sc4_SNP_IGA_416194
57.8
Sc4_SNP_IGA_415902
58.4
Sc4_SNP_IGA_417715
59.2
Sc4_Pp14Cl
60.6
Sc4_SNP_IGA_444204
67.0
Sc4_SNP_IGA_477945
70.0
a> 0
a<0
Sc4_SNP_IGA_369001
0.9
Sc4_Pp14Cl
2.7
Sc4_SNP_IGA_381379
4.7
Sc4_SNP_IGA_382420
5.4
Sc4_SNP_IGA_384731
7.0
Sc4_SNP_IGA_385272
7.6
Sc4_SNP_IGA_385899
7.9
Sc4_SNP_IGA_386286
8.1
Sc4_SNP_IGA_388388
9.4
Sc4_SNP_IGA_389208
9.7
Sc4_SNP_IGA_390559
10.5
Sc4_SNP_IGA_393507
11.5
Sc4_SNP_IGA_394550
12.0
Sc4_SNP_IGA_395202
12.3
Sc4_SNP_IGA_396351
Sc4_SNP_IGA_396443
12.7
Sc4_SNP_IGA_397228
13.1
Sc4_SNP_IGA_398213
13.8
Sc4_SNP_IGA_398368
13.9
Sc4_SNP_IGA_399599
14.3
Sc4_SNP_IGA_400126
14.5
ppa006310m
17.5
Sc4_SNP_IGA_407900
20.1
ppa006368m
20.6
Sc4_SNP_IGA_411147
21.8
Sc4_SNP_IGA_415799
25.4
Sc4_SNP_IGA_415902
Sc4_SNP_IGA_416194
25.5
Sc4_SNP_IGA_417715
26.2
ppa004664m
ppa025397m
34.4
Sc4_SNP_IGA_444204
37.7
ppa009359m
41.0
Sc4_SNP_IGA_477945
47.0
ppa012387m
59.3
Butyl acetate_EJ/AA
Butyl acetate_EJ/AA
2,2-Dimethylpropanoic acid_EJ/AA
C1
Candidate genes codification:
Lactones/Esters
Ethyl Acetate/Ethanol
Terpenoids
Lipid-derived compounds
Aromatic
(E)-2-Hexenyl acetate_EJ/AA
Hexanal_EJ/AA
HD_EJ/AA/IVIA
Firmness_EJ/AA/IVIA
g-Octalactone_EJ/AA
(E)-2-Hexenal_EJ/AA
C7
4-Methyl-5-penta-1,3-dienyltetrahydrofuran-2-one_EJ/AA
MnM_EJ/AA/IVIA
3-Methylbenzophenone_EJ/AA
C4
C6
C12
C8

## Slide 2
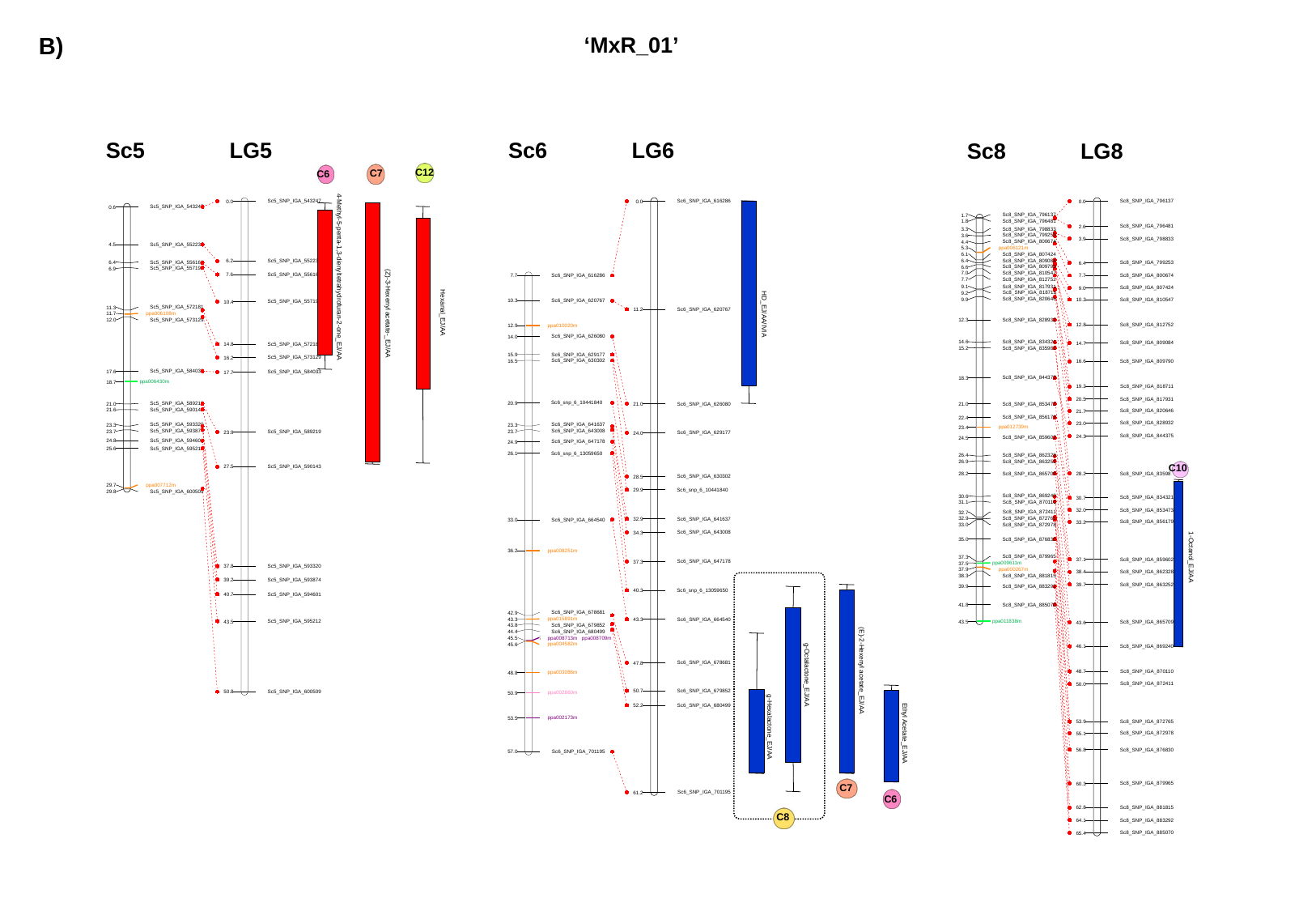

B)
‘MxR_01’
Sc6
LG6
Sc5
LG5
Sc6_SNP_IGA_616286
7.7
Sc6_SNP_IGA_620767
10.3
ppa010020m
12.9
Sc6_SNP_IGA_626080
14.0
Sc6_SNP_IGA_629177
15.9
Sc6_SNP_IGA_630302
16.5
Sc6_snp_6_10441840
20.9
Sc6_SNP_IGA_641637
23.3
Sc6_SNP_IGA_643008
23.7
Sc6_SNP_IGA_647178
24.9
Sc6_snp_6_13059650
26.1
Sc6_SNP_IGA_664540
33.0
ppa008251m
36.2
Sc6_SNP_IGA_678681
42.9
ppa015891m
43.3
Sc6_SNP_IGA_679852
43.8
Sc6_SNP_IGA_680499
44.4
ppa008713m
ppa008709m
45.5
ppa004582m
45.6
ppa003086m
48.8
ppa002860m
50.9
ppa002173m
53.5
Sc6_SNP_IGA_701195
57.0
Sc8_SNP_IGA_796137
1.7
Sc8_SNP_IGA_796481
1.8
Sc8_SNP_IGA_798833
3.3
Sc8_SNP_IGA_799253
3.6
Sc8_SNP_IGA_800674
4.4
ppa006121m
5.3
Sc8_SNP_IGA_807424
6.1
Sc8_SNP_IGA_809084
6.4
Sc8_SNP_IGA_809790
6.6
Sc8_SNP_IGA_810547
7.0
Sc8_SNP_IGA_812752
7.7
Sc8_SNP_IGA_817931
9.1
Sc8_SNP_IGA_818711
9.2
Sc8_SNP_IGA_820646
9.9
Sc8_SNP_IGA_828932
12.3
Sc8_SNP_IGA_834321
14.6
Sc8_SNP_IGA_835981
15.2
Sc8_SNP_IGA_844375
18.3
Sc8_SNP_IGA_853473
21.0
Sc8_SNP_IGA_856179
22.4
ppa012739m
23.4
Sc8_SNP_IGA_859602
24.5
Sc8_SNP_IGA_862328
26.4
Sc8_SNP_IGA_863252
26.9
Sc8_SNP_IGA_865709
28.2
Sc8_SNP_IGA_869240
30.6
Sc8_SNP_IGA_870110
31.1
Sc8_SNP_IGA_872411
32.7
Sc8_SNP_IGA_872765
32.9
Sc8_SNP_IGA_872978
33.0
Sc8_SNP_IGA_876830
35.0
Sc8_SNP_IGA_879965
37.3
ppa009611m
37.5
ppa000267m
37.9
Sc8_SNP_IGA_881815
38.3
Sc8_SNP_IGA_883292
39.9
Sc8_SNP_IGA_885070
41.8
ppa011838m
43.5
Sc8_SNP_IGA_796137
0.0
Sc8_SNP_IGA_796481
2.6
Sc8_SNP_IGA_798833
3.9
Sc8_SNP_IGA_799253
6.4
Sc8_SNP_IGA_800674
7.7
Sc8_SNP_IGA_807424
9.0
Sc8_SNP_IGA_810547
10.2
Sc8_SNP_IGA_812752
12.8
Sc8_SNP_IGA_809084
14.7
Sc8_SNP_IGA_809790
16.6
Sc8_SNP_IGA_818711
19.2
Sc8_SNP_IGA_817931
20.5
Sc8_SNP_IGA_820646
21.7
Sc8_SNP_IGA_828932
23.0
Sc8_SNP_IGA_844375
24.3
Sc8_SNP_IGA_835981
28.2
Sc8_SNP_IGA_834321
30.7
Sc8_SNP_IGA_853473
32.0
Sc8_SNP_IGA_856179
33.2
Sc8_SNP_IGA_859602
37.1
Sc8_SNP_IGA_862328
38.4
Sc8_SNP_IGA_863252
39.7
Sc8_SNP_IGA_865709
43.6
Sc8_SNP_IGA_869240
46.1
Sc8_SNP_IGA_870110
48.7
Sc8_SNP_IGA_872411
50.0
Sc8_SNP_IGA_872765
53.9
Sc8_SNP_IGA_872978
55.1
Sc8_SNP_IGA_876830
56.8
Sc8_SNP_IGA_879965
60.3
Sc8_SNP_IGA_881815
62.8
Sc8_SNP_IGA_883292
64.1
Sc8_SNP_IGA_885070
65.4
Sc8
LG8
C12
C7
C6
4-Methyl-5-penta-1,3-dienyltetrahydrofuran-2-one_EJ/AA
(Z)-3-Hexenyl acetate-_EJ/AA
Hexanal_EJ/AA
Sc5_SNP_IGA_543247
0.0
Sc5_SNP_IGA_543247
0.6
Sc5_SNP_IGA_552239
4.5
Sc5_SNP_IGA_552239
6.2
Sc5_SNP_IGA_556166
6.4
Sc5_SNP_IGA_557196
6.9
Sc5_SNP_IGA_556166
7.6
Sc5_SNP_IGA_557196
10.4
Sc5_SNP_IGA_572181
11.3
ppa006108m
11.7
Sc5_SNP_IGA_573129
12.0
Sc5_SNP_IGA_572181
14.8
Sc5_SNP_IGA_573129
16.2
Sc5_SNP_IGA_584033
17.6
Sc5_SNP_IGA_584033
17.7
ppa006430m
18.7
Sc5_SNP_IGA_589219
21.0
Sc5_SNP_IGA_590143
21.6
Sc5_SNP_IGA_593320
23.3
Sc5_SNP_IGA_593874
23.7
Sc5_SNP_IGA_589219
23.9
Sc5_SNP_IGA_594601
24.8
Sc5_SNP_IGA_595212
25.6
Sc5_SNP_IGA_590143
27.5
ppa007712m
29.7
Sc5_SNP_IGA_600509
29.8
Sc5_SNP_IGA_593320
37.8
Sc5_SNP_IGA_593874
39.2
Sc5_SNP_IGA_594601
40.7
Sc5_SNP_IGA_595212
43.5
Sc5_SNP_IGA_600509
50.8
0.0
11.2
21.0
24.0
28.5
29.9
32.9
34.3
37.3
40.3
43.3
47.8
50.7
52.2
61.2
Sc6_SNP_IGA_616286
HD_EJ/AA/IVIA
g-Octalactone_EJ/AA
g-Hexalactone_EJ/AA
Sc6_SNP_IGA_620767
Sc6_SNP_IGA_626080
Sc6_SNP_IGA_629177
C10
1-Octanol_EJ/AA
Sc6_SNP_IGA_630302
Sc6_snp_6_10441840
Sc6_SNP_IGA_641637
Sc6_SNP_IGA_643008
Sc6_SNP_IGA_647178
(E)-2-Hexenyl acetate_EJ/AA
Ethyl Acetate_EJ/AA
C7
C6
Sc6_snp_6_13059650
Sc6_SNP_IGA_664540
Sc6_SNP_IGA_678681
Sc6_SNP_IGA_679852
Sc6_SNP_IGA_680499
Sc6_SNP_IGA_701195
C8

## Slide 3
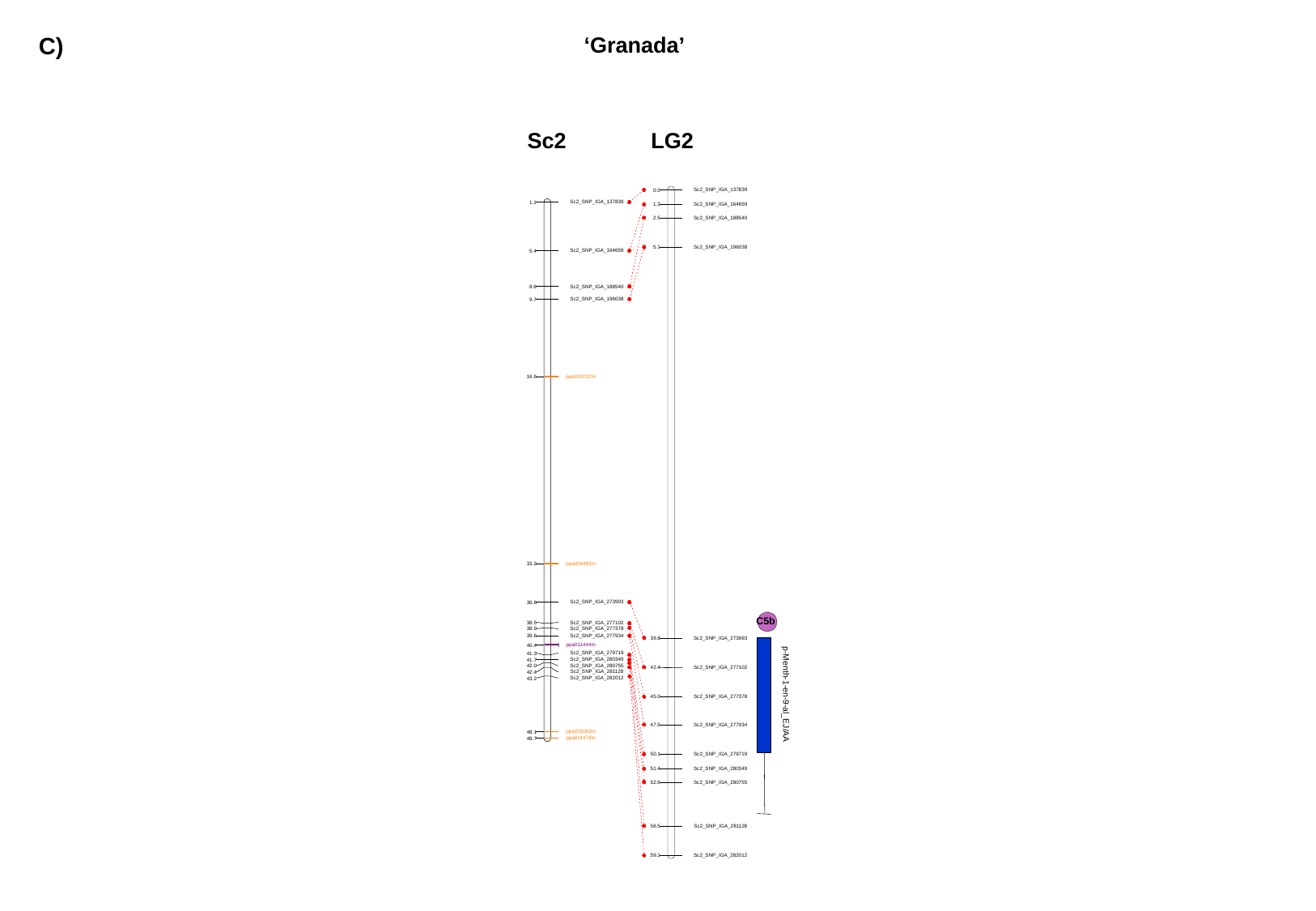

C)
‘Granada’
Sc2
LG2
Sc2_SNP_IGA_137839
0.0
Sc2_SNP_IGA_164659
1.3
Sc2_SNP_IGA_188540
2.5
Sc2_SNP_IGA_196038
5.1
Sc2_SNP_IGA_273993
39.8
Sc2_SNP_IGA_277102
42.4
Sc2_SNP_IGA_277378
45.0
Sc2_SNP_IGA_277934
47.5
Sc2_SNP_IGA_279719
50.1
Sc2_SNP_IGA_280349
51.4
Sc2_SNP_IGA_280755
52.6
Sc2_SNP_IGA_281128
56.5
Sc2_SNP_IGA_282012
59.1
Sc2_SNP_IGA_137839
1.1
Sc2_SNP_IGA_164659
5.4
Sc2_SNP_IGA_188540
8.6
Sc2_SNP_IGA_196038
9.7
ppa010737m
16.6
ppa004482m
33.2
Sc2_SNP_IGA_273993
36.6
Sc2_SNP_IGA_277102
38.5
Sc2_SNP_IGA_277378
38.9
Sc2_SNP_IGA_277934
39.6
ppa011444m
40.4
Sc2_SNP_IGA_279719
41.3
Sc2_SNP_IGA_280349
41.7
Sc2_SNP_IGA_280755
42.0
Sc2_SNP_IGA_281128
42.4
Sc2_SNP_IGA_282012
43.2
ppa010063m
48.1
ppa011478m
48.7
C5b
p-Menth-1-en-9-al_EJ/AA
