# Supplementary material for: The peach volatilome modularity is reflected at the genetic and environmental response levels in a QTL mapping population
Source: BMC Plant Biol. 2014 May 19;14:137. doi: 10.1186/1471-2229-14-137 (PMC4067740; doi:10.1186/1471-2229-14-137)
Supplement: Additional file 9: Figure S4 — Heatmap of LOD scores from volatile QTL analysis for ‘Granada’ at the EJ (top) and AA (bottom) locations. The LOD score (computed by single correlation analysis) for each marker/volatile pair is presented in a different color according to their additive effects (a), red for negatives a and blue for positive a. The color intensity is according to the LOD value, the higher the intensity the higher the LOD score. For each linkage group (LG1-LG2, LG4-LG8) the markers are ordered from left to right according to the position in the peach genome. The volatiles are ordered according to the position on the HCA of Figure 2. C1-C12 indicates the volatile clusters. Vertical and horizontal lines divide the linkage groups and the volatile clusters, respectively. EJ and AA indicate the locations of “El Jimeneo” and “Aguas Amargas”, respectively. [file 1471-2229-14-137-S9.pdf]

| N | id | Name | Cluster |
|---|---|---|---|
| 1 | 43_7136 | Butyl acetate | C1 |
| 3 | 41_6638 | 2,2-Dimethylpropanoic acid | C1 |
| 15 | 91_11117 | Benzeneacetaldehyde | C2b |
| 29 | 121_11693 | Terpinolene | C5 |
| 30 | 121_13280 | α-Terpinol | C5 |
| 31 | 139_9925 | 2H-Pyran, 2-ethenyltetrahydro-2,6,6-trimethyl- | C5 |
| 32 | 93_11733 | Linalool | C5 |
| 34 | 59_11450 | cis-Linaloloxide | C5 |
| 35 | 59_11676 | α-Methyl-a-[4-methyl-3-pentenyl]oxiranemethanol | C5 |
| 36 | 119_13188 | 4-Methylacetophenone | C5 |
| 37 | 138_12371 | 4-Acetyl-1-methylcyclohexene | C5 |
| 38 | 117_11758 | 3,4-Dimethylstyrene | C5 |
| 39 | 43_13124 | p-Cymen-8-ol | C5 |
| 40 | 123_10728 | β-Cyclocitral | C5 |
| 41 | 134_12449 | 1,3,8-p-Menthatriene | C5 |
| 42 | 137_13215 | 3,6-Dimethyl-2,3,3a,4,5,7a-hexahydrobenzofuran | C5 |
| 43 | 94_13611 | p-Menth-1-en-9-al | C5 |
| 44 | 69_13073 | cis-Carveol | C5 |
| 45 | 71_11820 | 3,7-Dimethyl-1,5,7-octatrien-3-ol | C5 |
| 49 | 68_15955 | 4-Methyl-5-penta-1,3-dienyltetrahydrofuran-2-one | C6b |
| 52 | 43_10351 | (E)-2-Hexenyl acetate | C7 |
| 53 | 80_10583 | 3-Cyclohexenyl acetate | C7 |
| 54 | 43_6475 | 2-Methylpropyl acetate | C8 |
| 56 | 95_16515 | 6-Pentyl-2H-pyran-2-one | C8 |
| 57 | 99_16910 | δ-Decalactone | C8 |
| 58 | 85_16556 | γ-Decalactone | C8 |
| 59 | 85_14019 | γ-Octalactone | C8 |
| 60 | 85_15326 | γ-Nonalactone | C8 |
| 61 | 68_16405 | γ-Jasmolactone | C8 |
| 62 | 85_12613 | γ-Heptalactone | C8 |
| 63 | 177_16736 | β-Ionone | C9 |
| 77 | 44_5291 | Pentanal | C12 |
| 79 | 56_6998 | Hexanal | C12 |
| 80 | 83_7941 | (E)-2-Hexenal | C12 |
| 81 | 164_15243 | Eugenol | C12b |

| Family | p | nM/M |
|---|---|---|
| Ester | 0.006 | 1.13 |
| Carboxilic acid | 0.024 | 1.33 |
| Aromatic | 0.000 | 0.46 |
| Terpenoid | 0.004 | 0.59 |
| Terpenoid | 0.009 | 0.63 |
| Terpenoid | 0.002 | 0.35 |
| Terpenoid | 0.001 | 0.25 |
| Terpenoid | 0.000 | 0.34 |
| un. | 0.000 | 0.41 |
| un. | 0.002 | 0.68 |
| Terpenoid | 0.000 | 0.37 |
| un | 0.007 | 0.55 |
| un. | 0.028 | 0.51 |
| Terpenoid | 0.006 | 0.32 |
| Terpenoid | 0.002 | 0.31 |
| un. | 0.000 | 0.26 |
| Terpenoid | 0.011 | 0.39 |
| un. | 0.001 | 0.30 |
| un. | 0.001 | 0.27 |
| Lactone | 0.000 | 1.76 |
| Ester | 0.012 | 0.48 |
| Ester | 0.041 | 1.36 |
| Ester | 0.022 | 1.90 |
| Lactone | 0.002 | 1.69 |
| Lactone | 0.001 | 1.95 |
| Lactone | 0.010 | 1.49 |
| Lactone | 0.000 | 1.93 |
| Lactone | 0.000 | 1.88 |
| Lactone | 0.000 | 2.15 |
| Lactone | 0.001 | 1.52 |
| Carotenoid-derived | 0.012 | 0.65 |
| Lipid-derived | 0.019 | 0.71 |
| Lipid-derived | 0.034 | 0.86 |
| Lipid-derived | 0.000 | 0.86 |
| Aromatic alcohol | 0.011 | 0.80 |
