# Supplementary material for: The peach volatilome modularity is reflected at the genetic and environmental response levels in a QTL mapping population
Source: BMC Plant Biol. 2014 May 19;14:137. doi: 10.1186/1471-2229-14-137 (PMC4067740; doi:10.1186/1471-2229-14-137)
Supplement: Additional file 15: Figure S5 — Co-localization of volatile QTL with candidate genes identified previously. Physical (left) and linkage (right) maps of chromosomes where volatile QTL were indentified are shown. The QTL are colored according to the direction of the additive (a) effect (blue for positive and red for negative). Bars and lines represent 1-LOD and 2-LOD support intervals. The candidate genes previously associated with different volatile groups [28] are indicated with a different color. The position of SNPs and candidate genes in the scaffolds of the peach genome v1 is indicated at the left of the map in arbitrary units (map position in base pair/ 5×10^5^). SNP positions in the linkage map are indicated at the right of the map in cM. A) QTL for LG4 of ‘MxR’ and the corresponding scaffold are shown. B) QTL for LG5 and LG6 of ‘MxR’ and the corresponding scaffolds are shown. C) QTL for LG2 of ‘Granada’ and the corresponding scaffold are shown. [file 1471-2229-14-137-S15.pdf]

Trait	1	3	5	6	7	9	10	11
Weight_AA	124.20	90.40	101.20	--	--	111.60	--	95.40
Weight_EJ	126.00	146.80	125.20	--	187.80	60.00	--	127.20
SSC_AA	18.70	18.50	15.40	--	--	14.60	--	17.60
SSC_EJ	15.20	14.90	14.90	--	14.40	11.60	--	13.60
SSC_IVIA	9.58	10.48	11.60	--	10.30	15.30	14.84	12.52
L_AA	18.78	22.88	26.63	--	--	19.98	--	28.02
L_EJ	21.90	31.90	24.66	--	20.56	26.04	--	27.23
C_AA	23.92	27.91	44.44	--	--	25.81	--	32.31
C_EJ	30.37	37.91	32.54	--	22.91	33.97	--	29.55
H_AA	85.44	81.81	67.50	--	--	79.49	--	77.59
H_EJ	77.21	66.61	78.56	--	89.39	65.72	--	77.72
MD_AA	48.00	42.00	0.00	--	--	21.00	--	48.00
MD_EJ	41.00	34.00	2.00	--	37.00	23.00	--	34.00
MD_IVIA	164.00	158.00	120.00	--	164.00	144.00	134.00	158.00
Firmness_AA	53.67	33.00	7.09	--	--	4.53	--	31.50
Firmness_EJ	34.25	20.00	20.18	--	51.50	1.97	--	39.58
Firmness_IVIA	27.65	23.83	15.10	--	29.62	2.84	20.30	35.70
MnM_AA	2	2	1	--	1	1	--	--
MnM_EJ	2	2	1	--	1	1	--	2
MnM-IVIA	2	2	--	--	2	0	0	2
43_7136_AA	-0.186	-0.035	-0.499	--	--	0.084	--	-1.478
43_7136_EJ	-0.123	0.221	-0.167	--	-0.213	0.314	--	-0.140
73_9306_AA	-0.276	-0.058	-0.319	--	--	0.071	--	0.212
73_9306_EJ	0.100	0.103	-0.072	--	-0.079	0.201	--	-0.054
41_6638_AA	-0.294	-1.295	-3.188	--	--	-1.391	--	-1.327
41_6638_EJ	0.108	0.548	0.247	--	0.301	0.051	--	-0.371
73_11030_AA	0.047	0.176	-0.086	--	--	0.459	--	0.429
73_11030_EJ	0.345	-0.362	0.420	--	1.635	0.065	--	0.092
165_16433_AA	-0.735	0.025	-0.438	--	--	0.179	--	0.270
165_16433_EJ	0.336	0.175	0.215	--	0.168	0.509	--	0.163
74_14613_AA	-0.588	-0.046	-0.420	--	--	0.380	--	-0.295
74_14613_EJ	0.322	0.135	0.157	--	0.195	0.762	--	-0.125
58_12978_AA	-0.555	-0.305	-0.387	--	--	0.076	--	0.134
58_12978_EJ	0.196	0.062	0.113	--	0.252	0.339	--	0.100
72_12911_AA	-0.594	-0.405	-0.439	--	--	0.102	--	0.048
72_12911_EJ	0.161	0.066	0.085	--	0.204	0.320	--	0.124
74_11980_AA	-0.562	-0.345	-0.575	--	--	0.073	--	0.040
74_11980_EJ	0.076	0.047	0.051	--	0.181	0.289	--	0.108
70_12284_AA	-0.524	-0.246	-0.447	--	--	0.306	--	0.044
70_12284_EJ	0.320	0.175	0.166	--	0.279	0.475	--	0.151
74_13339_AA	-0.527	-0.332	-0.477	--	--	0.083	--	-0.154
74_13339_EJ	0.118	0.067	0.027	--	0.17	0.519	--	0.019
74_16928_AA	-1.266	-0.323	-1.059	--	--	-0.251	--	-0.319
74_16928_EJ	0.324	-0.190	-0.343	--	-0.446	0.820	--	-0.229
70_13492_AA	-0.613	-0.227	-0.383	--	--	0.435	--	-1.372

70_13492_EJ	0.499	0.257	0.324	--	0.276	0.619	--	0.027
55_13398_AA	-0.529	-0.465	-0.971	--	--	-0.023	--	0.075
55_13398_EJ	1.927	0.136	-1.516	--	-0.58	3.973	--	0.006
91_11117_AA	-0.494	-2.836	-1.882	--	--	-2.623	--	-3.209
91_11117_EJ	0.010	-0.732	0.121	--	1	-1.211	--	-0.864
73_11681_AA	-0.129	-0.599	-0.106	--	--	0.571	--	0.202
73_11681_EJ	0.494	-0.283	0.548	--	1.351	0.232	--	0.308
73_12448_AA	-3.023	-0.823	-0.102	--	--	2.856	--	0.821
73_12448_EJ	0.868	-0.328	0.945	--	2.188	0.231	--	0.575
60_9627_AA	-0.403	-3.526	-3.627	--	--	3.368	--	0.103
60_9627_EJ	-5.266	3.582	0.000	--	7.281	-7.354	--	-3.823
73_13753_AA	-0.178	-2.436	-0.004	--	--	0.244	--	0.901
73_13753_EJ	1.418	-0.149	1.559	--	2.863	0.720	--	1.325
73_14964_AA	0.142	1.366	0.279	--	--	0.640	--	1.908
73_14964_EJ	1.278	0.196	2.604	--	3.285	0.734	--	1.480
172_17688_AA	-0.081	0.143	-0.174	--	--	0.384	--	0.317
172_17688_EJ	0.088	0.009	0.139	--	0.177	0.361	--	0.420
178_15640_AA	-0.217	0.045	0.223	--	--	0.546	--	0.345
178_15640_EJ	0.011	-0.028	0.258	--	0.272	0.077	--	0.132
105_18590_AA	-0.306	-0.068	-0.374	--	--	0.339	--	0.018
105_18590_EJ	-0.239	0.029	0.204	--	0.325	0.203	--	0.296
196_20012_AA	-0.259	0.155	-0.168	--	--	0.735	--	0.353
196_20012_EJ	0.329	0.216	0.446	--	0.472	0.453	--	0.471
108_12704_AA	-0.251	-0.163	-0.178	--	--	0.188	--	0.167
108_12704_EJ	-0.196	-0.010	-0.041	--	0.04	0.018	--	0.039
95_12787_AA	-0.195	-0.089	-0.150	--	--	0.147	--	0.247
95_12787_EJ	-0.044	0.058	0.027	--	0.112	0.096	--	0.048
105_14972_AA	-0.302	0.042	0.009	--	--	0.279	--	0.209
105_14972_EJ	-0.013	-0.017	0.171	--	0.246	0.083	--	0.197
120_14256_AA	-0.335	-0.185	-0.056	--	--	0.299	--	-0.052
120_14256_EJ	-0.522	-0.156	0.037	--	0.124	-0.086	--	0.026
121_11693_AA	-3.005	-2.673	-1.522	--	--	-2.327	--	-2.240
121_11693_EJ	-1.268	-2.237	0.939	--	-0.186	-1.982	--	0.687
121_13280_AA	-3.054	-2.439	-1.397	--	--	-2.028	--	-2.583
121_13280_EJ	-1.666	-2.304	0.566	--	-0.844	-2.719	--	0.494
139_9925_AA	-4.955	-4.872	-0.386	--	--	-4.514	--	-4.505
139_9925_EJ	-0.762	-2.722	1.495	--	0.445	-4.999	--	1.537
93_11733_AA	-8.309	-7.766	-4.221	--	--	-6.953	--	-7.729
93_11733_EJ	-2.427	-3.923	0.604	--	-1.033	-7.243	--	0.316
93_10867_AA	-0.847	-0.815	-0.560	--	--	-0.536	--	-1.203
93_10867_EJ	-0.800	-1.725	0.821	--	-0.08	-1.832	--	0.534
59_11450_AA	-3.046	-3.030	1.575	--	--	-3.478	--	-1.873
59_11450_EJ	-0.646	-1.726	1.817	--	1.284	-4.396	--	0.372
59_11676_AA	-2.763	-2.659	1.530	--	--	-2.609	--	-1.699
59_11676_EJ	-0.559	-1.779	1.721	--	1.272	-2.652	--	0.424
119_13188_AA	-0.445	-0.524	1.750	--	--	-0.881	--	0.275
119_13188_EJ	-0.105	0.102	1.948	--	1.416	-0.799	--	0.609

138_12371_AA	-2.547	-2.714	1.051	--	--	-3.092	--	-0.996
138_12371_EJ	-0.972	-0.836	2.041	--	0.548	-3.037	--	0.014
117_11758_AA	-0.459	-1.604	1.968	--	--	-2.261	--	-0.012
117_11758_EJ	0.251	-0.032	2.592	--	1.976	-2.602	--	1.354
43_13124_AA	-2.420	-2.733	1.742	--	--	-4.793	--	-0.254
43_13124_EJ	-0.482	-0.310	1.700	--	0.869	-2.684	--	0.568
123_10728_AA	-1.421	-0.896	3.162	--	--	-5.399	--	0.570
123_10728_EJ	1.001	0.556	3.541	--	2.959	-5.315	--	2.142
134_12449_AA	-4.450	-3.228	1.764	--	--	-5.079	--	-0.934
134_12449_EJ	-0.078	-0.331	2.397	--	1.308	-5.629	--	0.920
137_13215_AA	-3.767	-2.817	1.048	--	--	-4.461	--	-1.769
137_13215_EJ	-1.593	-1.153	2.102	--	0.581	-6.001	--	0.689
94_13611_AA	-5.483	-3.506	0.215	--	--	-5.591	--	-3.321
94_13611_EJ	-2.011	-1.638	0.498	--	-0.862	-7.110	--	-1.235
69_13073_AA	-6.565	-4.909	1.043	--	--	-5.836	--	-4.672
69_13073_EJ	-2.545	0.626	2.312	--	-1.569	-5.472	--	-0.249
71_11820_AA	-8.540	-6.812	1.164	--	--	-7.633	--	-5.361
71_11820_EJ	-3.234	-3.116	0.756	--	-2.045	-6.714	--	-1.070
119_10786_AA	1.147	-0.367	0.848	--	--	-0.895	--	1.123
119_10786_EJ	1.104	0.154	1.317	--	1.986	-0.679	--	1.752
43_3992_AA	-2.066	-1.048	-0.538	--	--	0.001	--	-0.487
43_3992_EJ	-1.478	-1.548	-1.721	--	-2.413	2.067	--	-2.255
45_2418_AA	1.438	1.342	0.417	--	--	3.402	--	2.479
45_2418_EJ	2.231	2.814	2.703	--	1.148	5.110	--	2.685
68_15955_AA	0.251	1.649	0.654	--	--	2.032	--	2.695
68_15955_EJ	0.886	0.572	-0.645	--	-0.152	1.520	--	1.669
43_10318_AA	-5.153	0.001	0.848	--	--	0.019	--	-0.211
43_10318_EJ	0.176	1.202	-0.116	--	-4.789	-0.156	--	-0.746
67_10239_AA	-0.851	0.365	-0.159	--	--	0.087	--	0.752
67_10239_EJ	0.967	0.174	-0.561	--	-1.608	-1.465	--	-0.656
43_10351_AA	-5.604	-3.940	0.387	--	--	-0.203	--	-2.723
43_10351_EJ	-0.628	0.232	0.116	--	-5.352	-1.427	--	-1.606
80_10583_AA	0.037	1.777	-0.422	--	--	0.216	--	1.620
80_10583_EJ	1.189	0.213	-0.156	--	-1.632	-2.978	--	-1.660
43_6475_AA	-3.917	-0.361	-0.364	--	--	-0.632	--	-0.841
43_6475_EJ	-0.195	0.072	-2.730	--	-3.477	0.174	--	-3.475
85_11181_AA	-0.878	-0.293	0.108	--	--	0.380	--	-0.594
85_11181_EJ	0.278	0.423	0.339	--	-0.637	-0.116	--	-0.674
95_16515_AA	-0.917	1.220	1.349	--	--	0.858	--	0.169
95_16515_EJ	1.057	0.978	0.236	--	-2.247	0.268	--	-1.017
99_16910_AA	-2.459	0.780	0.123	--	--	0.593	--	0.333
99_16910_EJ	0.484	0.388	-0.767	--	-3.219	0.484	--	-1.297
85_16556_AA	-1.817	0.886	0.379	--	--	0.762	--	0.463
85_16556_EJ	0.518	0.395	-0.748	--	-2.804	0.330	--	-1.048
85_14019_AA	-0.640	0.703	0.383	--	--	0.598	--	0.299
85_14019_EJ	0.836	1.266	-0.473	--	-1.512	0.685	--	-0.660
85_15326_AA	-0.265	1.035	0.671	--	--	0.721	--	0.943

85_15326_EJ	0.855	0.956	-0.368	--	-0.816	0.498	--	-0.208
68_16405_AA	-1.246	0.568	0.446	--	--	0.426	--	0.718
68_16405_EJ	0.880	0.782	-0.678	--	-2.519	0.185	--	-0.804
85_12613_AA	-0.574	0.133	-0.158	--	--	0.335	--	0.190
85_12613_EJ	0.836	0.975	0.001	--	-1.41	1.043	--	-1.014
177_16736_AA	-0.159	0.751	0.733	--	--	2.809	--	1.883
177_16736_EJ	4.740	-1.009	0.985	--	1.845	0.642	--	2.323
69_15595_AA	0.274	1.020	0.100	--	--	1.504	--	1.397
69_15595_EJ	1.383	0.506	-0.069	--	0.128	1.609	--	0.856
108_9983_AA	-0.619	-0.402	-0.091	--	--	0.535	--	0.305
108_9983_EJ	-0.236	-0.186	-0.531	--	-0.515	-0.025	--	-0.504
43_16192_AA	-0.221	0.923	1.258	--	--	3.837	--	3.864
43_16192_EJ	2.205	2.344	0.559	--	2.381	2.357	--	1.820
56_12707_AA	-0.281	-0.114	-0.086	--	--	0.247	--	1.161
56_12707_EJ	-0.017	-0.139	0.169	--	0.585	0.152	--	0.182
56_9747_AA	-1.575	-0.204	-0.388	--	--	-1.149	--	0.958
56_9747_EJ	-0.068	-0.011	-0.043	--	0.036	-0.067	--	-0.046
45_3430_AA	0.000	0.331	0.000	--	--	0.000	--	0.461
45_3430_EJ	0.318	-0.038	-0.169	--	-0.315	0.078	--	0.162
57_10633_AA	-0.053	0.137	-0.502	--	--	0.190	--	0.255
57_10633_EJ	-0.099	1.268	-1.582	--	-2.674	0.124	--	0.161
56_11249_AA	-0.238	-0.350	-0.467	--	--	0.577	--	0.049
56_11249_EJ	-0.124	-0.061	0.138	--	-1.497	0.085	--	-0.430
57_5029_AA	0.493	0.690	0.376	--	--	0.709	--	1.068
57_5029_EJ	0.096	0.283	1.244	--	1.08	-0.511	--	-0.132
69_6953_AA	2.787	1.432	-0.265	--	--	1.100	--	3.320
69_6953_EJ	0.576	-1.051	0.025	--	0.629	-0.846	--	0.583
81_5323_AA	3.120	1.679	0.478	--	--	1.612	--	3.336
81_5323_EJ	0.677	-0.255	0.859	--	0.989	-0.271	--	1.531
55_5080_AA	2.133	1.797	0.844	--	--	2.188	--	3.028
55_5080_EJ	0.340	-0.872	0.476	--	0.15	-2.009	--	0.712
81_10494_AA	2.452	1.670	1.476	--	--	2.041	--	2.707
81_10494_EJ	0.022	-0.537	1.107	--	1.085	-1.036	--	0.644
44_5291_AA	2.182	3.370	1.864	--	--	2.697	--	3.186
44_5291_EJ	-0.814	0.531	3.047	--	2.861	0.027	--	1.226
81_10119_AA	1.567	0.959	1.654	--	--	2.170	--	2.731
81_10119_EJ	0.386	-0.030	1.746	--	0.742	0.089	--	0.848
56_6998_AA	-0.415	-0.664	-0.142	--	--	0.547	--	0.311
56_6998_EJ	-0.310	-1.311	-0.091	--	-0.342	-0.749	--	0.266
83_7941_AA	0.188	-0.013	-0.150	--	--	0.430	--	0.267
83_7941_EJ	-0.153	-0.574	0.191	--	0.334	-0.489	--	0.311
164_15243_AA	1.772	0.420	1.130	--	--	1.312	--	1.990
164_15243_EJ	0.149	0.251	1.357	--	0.050	-0.097	--	0.592

12	14	15	16	17	18	19	20	21	22
155.20	96.80	142.60	151.00	--	114.60	158.60	98.80	92.60	--
69.80	76.40	69.40	162.60	150.80	104.60	172.40	85.00	107.00	126.40
15.80	14.80	13.10	14.70	--	12.30	15.10	16.10	18.80	--
15.20	14.30	15.20	12.30	14.60	14.50	14.30	13.60	12.80	12.30
13.42	11.72	11.28	--	12.88	13.14	19.58	13.10	13.56	12.38
17.46	19.70	24.09	26.20	--	21.35	19.29	18.09	22.12	--
22.03	26.33	30.59	21.89	21.99	28.78	19.75	28.86	27.26	22.48
24.00	22.77	34.03	24.62	--	28.03	22.25	24.07	28.77	--
25.03	29.78	42.43	27.59	26.00	35.25	25.81	33.63	32.29	26.97
83.08	93.78	74.09	87.02	--	87.76	88.82	83.10	83.02	--
85.30	83.50	71.44	83.27	84.27	74.69	80.38	79.02	81.66	76.76
42.00	6.00	2.00	48.00	--	9.00	28.00	28.00	42.00	--
27.00	6.00	2.00	37.00	27.00	9.00	20.00	16.00	23.00	27.00
158.00	124.00	124.00	164.00	--	124.00	152.00	144.00	152.00	152.00
8.00	--	9.32	33.42	--	3.10	21.50	4.30	40.33	--
31.00	5.52	2.37	29.25	8.25	1.78	11.23	2.07	40.67	30.00
6.57	38.54	8.63	22.65	--	42.56	8.73	3.73	11.77	15.49
1	1	1	1	1	--	1	1	2	2
1	1	1	1	1	--	1	1	2	2
0	--	--	2	--	--	0	0	1	1
0.046	-0.381	-0.376	0.009	--	-0.434	0.041	-0.416	-1.008	--
0.415	-0.067	0.447	0.210	0.272	0.151	-0.169	-0.237	-0.343	-0.022
0.211	-0.235	-0.227	0.089	--	-0.436	0.056	-0.212	-0.555	--
0.310	-0.090	0.276	0.368	0.183	0.013	-0.006	0.057	0.009	-0.118
-0.271	-1.813	-0.190	-1.013	--	-2.558	-0.109	-3.059	-1.882	--
0.477	0.341	0.321	0.467	0.243	0.592	-0.005	0.042	0.036	0.378
0.053	0.247	-0.425	0.184	--	0.340	0.257	-0.346	-0.043	--
0.881	5.581	3.464	0.468	-1.768	1.080	-0.071	-0.036	5.458	1.059
0.197	-0.104	0.210	-0.112	--	0.190	-0.106	-0.528	-0.236	--
0.195	0.134	0.319	0.353	0.341	0.641	0.440	0.402	0.675	0.375
-0.049	-0.011	-0.216	-0.265	--	-0.291	-0.150	-0.689	-0.709	--
0.374	-0.047	0.310	0.287	0.347	0.399	0.272	0.424	0.523	0.114
-0.191	-0.226	-0.267	-0.447	--	-0.050	-0.147	-0.236	-0.607	--
0.099	-0.084	0.287	0.295	0.353	0.404	0.169	0.336	0.452	0.212
-0.208	-0.319	-0.225	-0.500	--	-0.057	-0.280	-0.402	-0.698	--
0.086	-0.043	0.300	0.320	0.327	0.390	0.165	0.285	0.483	0.127
-0.320	-0.182	-0.311	-0.484	--	0.121	-0.253	-0.235	-0.649	--
0.096	-0.089	0.327	0.289	0.315	0.365	0.117	0.257	0.363	0.121
-0.254	-0.189	-0.253	-0.433	--	0.344	-0.133	-0.303	-0.654	--
0.270	-0.015	0.355	0.384	0.394	0.442	0.272	0.364	0.453	0.247
-0.212	-0.395	-0.442	-0.523	--	-0.225	-0.176	-0.387	-0.588	--
0.241	-0.133	0.330	0.253	0.323	0.384	0.140	0.243	0.357	0.04
-0.408	-0.880	-0.383	-0.510	--	-0.756	-0.550	-0.774	-0.576	--
0.300	-0.409	0.064	0.216	-0.55	0.488	-0.011	-0.080	0.345	0.123
0.052	0.199	-0.030	-0.335	--	0.201	-0.038	-0.438	-0.665	--

0.454	-1.218	0.389	0.380	0.543	0.461	0.385	0.509	0.631	0.251
0.152	1.224	0.100	-1.257	--	1.541	-1.622	-1.507	-3.429	--
1.497	-1.326	0.024	1.452	-0.918	0.601	1.304	0.559	0.403	0.709
-3.263	-0.035	-2.957	-3.461	--	-1.597	-0.076	-0.921	-2.003	--
1.911	-0.036	1.684	-0.413	-0.52	-0.569	0.170	0.572	0.781	-2.176
0.348	-0.032	-0.051	-0.269	--	0.931	-0.512	-0.026	-0.248	--
0.958	-0.198	0.326	0.519	1.333	1.041	0.112	0.094	-0.205	0.92
0.451	-4.863	-0.269	-0.402	--	2.604	-0.113	-0.006	-0.365	--
1.108	-0.213	0.844	0.661	1.7	1.626	0.210	0.091	-0.276	1.431
3.435	3.639	-0.265	-5.415	--	6.679	-0.159	-0.040	-0.007	--
7.069	7.128	3.555	7.121	3.456	7.176	-3.767	-0.001	3.412	7.074
0.159	0.038	-2.890	-2.457	--	0.281	-0.049	-0.234	-0.234	--
1.445	0.120	1.078	0.805	2.677	2.447	0.849	0.448	-0.060	2.036
0.240	-0.003	-0.365	-0.193	--	0.289	0.164	-0.324	-0.205	--
1.352	0.565	2.181	1.003	2.561	2.909	0.900	1.088	0.417	2.185
0.365	-0.238	0.144	0.117	--	0.394	0.416	-0.577	-0.323	--
0.205	-0.025	0.335	0.542	0.045	0.552	-0.025	-0.155	0.376	0.491
0.200	-0.103	0.108	0.051	--	0.212	0.116	-0.345	-0.363	--
0.054	-0.141	0.321	0.397	0.312	0.422	0.034	-0.055	0.382	0.194
-0.041	-0.268	-0.147	-0.132	--	0.094	0.164	-0.782	-0.321	--
0.203	-0.155	0.229	0.529	0.166	0.522	0.016	-0.263	0.418	0.4
0.186	0.155	0.197	-0.053	--	0.634	0.095	-0.679	-0.439	--
0.274	0.082	0.209	0.719	0.459	0.817	0.158	0.052	0.923	0.724
-0.107	-0.316	-0.301	-0.062	--	0.081	-0.130	-0.312	-0.507	--
0.049	-0.194	0.333	0.185	0.048	0.243	-0.020	-0.009	0.062	0.062
-0.049	-0.234	-0.328	-0.018	--	-0.170	0.239	-0.250	-0.449	--
0.157	-0.039	0.284	0.193	0.161	0.213	0.004	-0.050	-0.023	0.037
0.070	-0.216	-0.280	0.116	--	0.013	0.189	-0.490	-0.523	--
0.090	-0.085	0.294	0.300	0.265	0.445	0.069	-0.065	0.220	0.206
-0.055	-0.381	-0.446	-0.280	--	-0.136	-0.101	-0.531	-0.724	--
0.079	-0.195	0.188	0.301	0.426	0.274	-0.185	-0.330	0.030	-0.059
-2.533	-1.537	-1.267	-3.189	--	-0.481	-1.958	-2.505	-2.781	--
-2.750	-2.085	-1.544	-0.110	0.051	-2.293	-2.124	2.014	1.163	1.472
-1.945	-1.697	-1.227	-2.415	--	-0.819	-2.577	-2.083	-2.474	--
-2.932	-2.662	-2.168	-0.132	-0.486	-2.991	-2.850	1.682	0.979	1.05
-4.843	-1.174	-0.216	-4.784	--	0.269	-4.368	-4.891	-4.891	--
-4.877	-2.512	-0.795	0.256	0.324	-2.389	-4.374	2.635	1.696	2.386
-7.067	-2.603	-2.793	-8.100	--	-1.310	-7.900	-6.203	-6.990	--
-6.883	-4.128	-3.524	-0.083	0.013	-5.330	-5.597	1.836	1.221	0.998
-0.591	-0.466	-0.580	-0.831	--	0.236	-0.836	-0.799	-1.102	--
-2.861	-1.600	-0.944	-0.241	0.265	-2.068	-1.693	1.572	0.869	0.946
-2.977	0.344	1.448	-1.591	--	0.641	-1.215	-1.234	-2.795	--
-2.363	-0.642	0.967	0.381	0.379	-0.568	-0.509	0.765	1.230	0.511
-2.295	0.194	1.186	-1.625	--	0.618	-1.263	-1.231	-2.517	--
-2.308	-0.756	0.774	0.372	0.386	-0.535	-0.627	0.785	1.111	0.559
-0.619	0.296	1.070	-0.284	--	0.919	-0.339	-0.358	-1.173	--
-0.372	0.127	1.355	0.311	0.92	0.450	-0.067	0.600	0.840	1.171

-1.200	-0.056	1.093	-1.080	--	0.254	-1.412	-0.342	-1.909	--
-1.826	-0.801	0.678	-0.236	0.02	-0.599	-0.669	1.130	0.577	0.227
-2.307	1.014	1.498	-1.287	--	1.957	-0.481	-1.115	-2.156	--
-1.372	0.483	2.105	0.209	1.246	1.066	-0.012	1.483	1.512	1.974
-3.476	0.630	1.862	-1.693	--	1.359	-1.995	-2.415	-3.181	--
-3.383	0.084	0.977	0.296	0.589	0.421	-1.882	2.605	0.792	1.235
-1.857	2.090	2.469	-1.106	--	2.493	0.022	-0.550	-3.200	--
-0.808	1.331	3.214	0.449	2.118	2.168	0.622	2.167	2.025	2.863
-3.234	0.609	1.717	-1.707	--	1.304	-1.749	-0.826	-3.358	--
-1.809	-0.103	2.181	-0.002	1.093	0.843	-0.251	1.792	1.258	1.625
-2.088	0.301	0.564	-2.351	--	0.908	-1.615	-1.217	-3.334	--
-1.858	-0.163	0.499	-0.566	0.101	0.061	-0.302	1.264	1.046	0.885
-2.850	-1.134	0.253	-2.908	--	-0.721	-3.463	-1.716	-3.469	--
-2.576	-1.438	-1.614	-2.811	-0.957	-1.558	-1.708	0.307	-0.141	-0.967
-5.827	-0.544	1.256	-3.765	--	-1.948	-4.772	-0.763	-6.061	--
-1.247	-1.270	-0.456	-0.133	-0.388	-1.659	-2.741	1.232	0.489	-0.03
-6.234	-2.508	-0.015	-4.931	--	-1.121	-7.237	-3.179	-5.117	--
-3.991	-3.342	0.447	-0.848	-1.772	-2.152	-2.598	1.532	-0.072	-1.107
-2.027	0.347	0.206	-0.169	--	0.539	0.952	-1.617	-1.381	--
-1.585	-0.028	0.549	0.814	0.502	0.186	0.812	0.456	0.667	1.357
2.151	-2.470	1.794	1.031	--	-0.606	-1.208	3.819	-2.858	--
-2.403	-0.071	1.361	-2.087	-2.227	2.195	3.995	1.667	-2.234	-2.377
5.219	1.913	5.502	2.437	--	3.473	2.158	2.928	-0.758	--
2.063	1.631	0.655	1.288	2.711	2.205	5.728	4.046	0.918	1.798
2.291	-0.983	2.163	2.093	--	1.413	0.320	1.476	1.213	--
-1.069	-0.396	0.582	-0.069	-0.836	0.497	0.090	0.398	1.212	1.857
1.593	-2.137	1.370	1.253	--	-0.134	-1.063	1.488	-0.825	--
-0.804	0.136	0.665	-3.601	-0.11	1.331	1.243	0.932	-3.375	-0.138
1.614	-0.653	0.463	0.753	--	-0.772	-0.386	0.699	-1.057	--
0.411	0.351	-0.064	-1.335	0.57	0.643	0.619	0.300	-1.997	0.4
0.843	-0.201	0.697	0.479	--	-0.313	-3.088	1.862	-6.012	--
-0.340	0.731	1.225	-4.040	0.445	1.342	1.414	1.366	-5.481	-0.898
2.112	-0.883	-0.263	0.962	--	-0.601	0.881	0.137	-0.425	--
0.530	-0.037	0.080	-1.836	0.036	0.720	0.846	-0.859	-2.620	-0.833
0.389	-3.735	1.210	1.123	--	-3.701	-3.194	1.952	-3.948	--
-2.862	0.331	2.026	-2.835	-2.172	1.238	0.666	1.298	-3.460	-3.292
0.137	0.036	1.312	0.682	--	-0.120	-0.941	0.543	-0.969	--
-0.921	0.292	1.219	-0.082	-0.568	0.149	0.008	0.228	-0.322	-0.143
1.401	-1.157	0.965	1.165	--	0.248	-1.706	1.091	0.807	--
-0.908	0.863	1.316	-1.273	-0.448	1.181	0.467	1.244	-0.094	0.111
1.372	-1.269	2.518	2.084	--	-0.862	-1.850	0.855	-0.285	--
-1.014	0.475	0.559	-0.842	-0.345	0.522	0.285	0.614	-1.621	0.106
1.328	-1.341	1.249	1.244	--	-0.082	-1.548	0.779	0.156	--
-1.197	0.345	0.178	-0.703	-0.63	0.292	0.237	0.580	-1.585	0.284
1.246	-1.578	1.017	1.639	--	-0.515	-0.936	0.797	-0.285	--
-1.197	0.133	0.801	-0.225	-0.593	0.704	0.222	0.092	-1.150	0.109
1.097	-1.029	1.130	2.150	--	-0.004	-0.798	0.729	0.166	--

-0.786	0.482	0.877	-0.179	-0.211	0.941	0.355	0.177	-0.395	0.124
1.571	-1.497	2.864	2.565	--	-0.333	-1.554	0.821	-0.271	--
-1.342	0.197	1.782	-0.567	-1.132	0.709	-0.092	0.317	-1.433	-0.012
1.231	-0.637	0.494	1.107	--	-0.105	-0.253	0.624	-0.500	--
-0.809	0.071	0.999	-0.047	-0.299	0.852	0.214	-0.074	-1.285	-0.118
2.767	2.619	1.959	1.014	--	3.670	0.850	0.747	0.225	--
0.100	2.156	0.457	-0.023	1.402	2.590	1.236	3.650	3.411	0.572
1.081	0.407	1.260	0.528	--	1.005	0.971	-0.026	-0.001	--
0.537	-0.056	0.513	0.291	-0.147	0.725	0.184	0.927	1.500	1.381
0.577	0.153	0.305	0.529	--	0.190	0.154	0.461	-0.384	--
-0.423	-0.089	-0.431	-0.449	-0.704	-0.158	-0.482	0.071	-0.049	-0.25
2.904	1.042	3.241	0.904	--	4.017	2.970	1.430	-0.382	--
-0.087	1.666	1.291	-1.257	2.252	2.541	1.790	4.103	2.903	1.603
0.326	-0.282	0.023	-0.381	--	0.084	-0.552	-0.172	-0.344	--
-0.039	0.221	0.139	0.130	-0.063	0.411	-0.036	0.242	0.256	0.462
0.359	0.111	-0.250	-0.210	--	0.344	-0.034	-0.136	-0.249	--
-0.045	0.026	0.048	-0.045	0	0.084	0.000	0.000	-0.081	0.007
0.000	0.000	0.000	0.344	--	0.000	0.478	0.070	0.250	--
-0.103	-0.033	-0.262	0.072	0.094	-0.303	0.075	-0.142	-0.146	-0.082
0.588	0.266	-0.083	0.007	--	0.266	-0.128	-0.426	-0.605	--
-2.448	-0.321	-1.591	-0.097	-2.475	-0.723	-5.105	-4.457	0.565	-0.339
0.040	-1.848	-0.006	-0.431	--	-1.523	-0.142	-0.232	-0.546	--
-0.272	-0.177	0.207	0.241	0.072	-1.553	-1.658	0.259	0.352	-1.283
0.964	0.550	0.706	1.118	--	0.450	0.616	0.522	-0.158	--
0.284	0.113	0.204	-0.435	0.275	0.398	0.162	3.133	0.525	-0.003
1.109	0.570	0.276	0.726	--	0.328	0.545	-0.328	1.964	--
-0.564	-0.071	-0.611	0.506	-0.784	-0.522	-0.230	-0.675	0.632	-0.58
1.194	2.700	0.743	0.703	--	2.504	0.971	0.334	1.613	--
-1.210	0.651	0.181	0.017	-0.101	0.633	0.585	-0.159	0.981	0.943
2.106	1.921	0.206	2.590	--	2.308	1.994	0.658	1.926	--
-0.382	0.189	-0.423	0.711	-0.568	-0.642	-0.085	-1.134	0.790	-1.147
1.325	2.051	1.178	1.807	--	1.890	2.002	0.692	1.248	--
-0.417	0.772	0.431	-0.104	-0.384	-0.327	0.006	-0.353	0.737	-0.466
1.500	2.446	0.424	2.068	--	2.404	3.002	1.069	1.220	--
1.182	2.668	1.815	1.473	0.775	2.119	2.629	0.542	1.354	2.099
1.197	1.866	1.599	1.576	--	2.916	1.906	1.355	0.950	--
-0.976	1.152	0.818	-0.130	-0.035	1.278	0.968	0.329	0.721	0.579
0.377	0.020	0.067	0.256	--	0.299	0.378	0.038	-0.308	--
-0.891	-0.287	-0.693	0.035	-0.74	-0.032	0.018	-0.584	0.056	0.015
0.306	0.184	-0.359	0.098	--	0.109	0.419	-0.114	-0.416	--
-0.158	0.055	-0.290	0.068	-0.098	-0.166	0.059	-0.175	0.309	-0.078
0.408	1.824	-0.072	0.749	--	1.566	1.350	0.924	0.404	--
-0.260	1.371	1.094	-0.178	0.507	0.949	0.643	0.980	0.379	0.105

23	24	28	30	31	33	34	35	36	37
120.60	132.00	130.80	88.40	--	121.40	--	115.40	87.20	92.20
101.60	98.80	220.00	151.00	107.80	109.80	--	198.20	85.20	106.20
13.00	19.20	19.30	18.80	--	13.80	--	16.80	17.40	16.40
12.90	--	12.90	13.70	14.20	13.40	--	13.20	13.90	15.10
--	12.32	10.00	12.22	12.84	--	13.00	15.82	15.50	13.68
25.02	19.89	24.31	22.12	--	23.90	--	19.31	20.23	21.44
26.34	24.81	24.80	23.74	20.82	24.74	--	19.66	27.44	28.33
30.81	26.68	38.78	26.61	--	31.13	--	23.60	29.94	30.41
36.22	27.98	29.55	31.79	24.05	28.91	--	24.81	30.21	34.62
83.23	82.04	66.00	83.00	--	82.07	--	88.72	76.96	81.45
71.94	86.06	81.80	75.13	85.09	84.71	--	86.43	82.59	74.27
6.00	58.00	55.00	48.00	--	14.00	--	42.00	28.00	28.00
6.00	41.00	34.00	37.00	9.00	9.00	--	30.00	13.00	20.00
124.00	--	164.00	164.00	124.00	144.00	--	158.00	134.00	134.00
4.18	21.45	--	15.45	--	28.92	--	28.83	13.28	17.73
3.12	22.58	30.58	14.33	2.47	34.25	--	--	26.67	25.67
28.73	--	23.73	14.32	27.75	32.17	--	10.30	21.38	22.46
1	1	2	1	--	2	2	1	2	2
1	1	2	1	--	2	2	1	2	2
--	--	2	1	--	2	--	2	2	2
-0.454	-0.217	0.182	0.047	--	-0.323	--	-0.443	0.005	0.157
-0.079	--	0.118	0.310	0.22	0.116	--	0.172	0.052	-0.042
-0.030	0.062	-0.175	-0.271	--	-0.107	--	-0.013	-0.156	0.175
0.105	--	0.418	0.050	0.172	0.182	--	0.268	0.137	-0.007
-0.494	-0.454	0.373	0.000	--	-1.561	--	0.022	0.485	-0.019
0.237	--	1.062	0.740	-0.03	0.447	--	0.105	0.421	-0.125
0.259	0.521	-0.227	-0.310	--	-0.040	--	-0.264	-0.274	0.082
2.656	--	-0.018	2.862	2.942	1.083	--	2.792	4.085	-2.815
0.396	-0.095	-0.371	-0.530	--	0.476	--	0.524	-0.003	0.375
0.111	--	0.451	0.353	0.741	0.304	--	0.445	0.350	0.446
0.216	-0.338	-0.458	-0.541	--	-0.241	--	0.541	-0.203	-0.018
0.168	--	0.253	0.159	0.714	0.027	--	0.466	0.061	0.060
0.059	-0.33	-0.451	-0.710	--	0.014	--	0.175	-0.482	-0.226
0.098	--	0.351	0.107	0.663	0.255	--	0.305	0.270	0.290
0.038	-0.413	-0.468	-0.608	--	0.042	--	0.098	-0.474	-0.374
0.063	--	0.264	0.096	0.628	0.212	--	0.203	0.203	0.289
-0.031	-0.385	-0.603	-0.725	--	0.000	--	0.041	-0.657	-0.219
0.037	--	0.298	0.113	0.628	0.266	--	0.280	0.269	0.228
-0.080	-0.435	-0.572	-0.465	--	-0.045	--	0.229	-0.520	-0.146
0.106	--	0.418	0.231	0.613	0.226	--	0.455	0.252	0.350
-0.012	-0.411	-0.479	-0.643	--	-0.122	--	0.208	-0.383	-0.205
0.024	--	0.261	0.045	0.661	0.045	--	0.234	0.021	0.232
-0.203	-0.568	-0.882	-1.536	--	-0.415	--	0.123	-0.841	-0.094
-0.446	--	-0.223	0.015	0.007	0.066	--	0.242	0.102	0.129
0.236	-0.402	-0.245	-0.434	--	0.056	--	0.518	-0.372	-0.028

0.249	--	0.262	0.257	0.743	0.142	--	0.665	0.256	0.255
0.417	-1.619	0.146	1.093	--	-1.515	--	0.138	-0.152	0.593
0.431	--	1.007	2.670	0.193	-0.581	--	2.535	0.145	2.572
-0.138	-2.166	-3.229	-2.683	--	-2.396	--	-0.386	-3.512	-1.348
-0.030	--	-1.703	-0.396	-0.972	-3.511	--	-0.514	-2.097	-1.066
-0.245	0.45	-0.089	-0.090	--	0.013	--	-0.345	-0.512	-0.085
-0.138	--	-0.175	-0.120	0.077	-1.020	--	0.157	1.098	0.216
-0.224	0.576	0.080	0.200	--	0.047	--	-0.504	-0.665	-0.157
-0.076	--	0.029	0.058	0.202	1.594	--	0.071	1.721	0.182
-3.677	3.53	-0.009	-6.966	--	3.561	--	-3.795	-0.420	-2.722
3.492	--	-0.056	-0.005	-0.065	1.683	--	3.591	9.869	-7.299
0.258	0.7	1.106	-2.805	--	0.069	--	-0.066	-0.254	-2.088
0.186	--	0.076	0.145	0.869	2.263	--	0.429	2.475	0.454
0.262	-0.029	-0.010	-0.108	--	-0.075	--	-0.196	-0.295	-0.100
0.821	--	0.266	0.427	1.307	2.798	--	0.531	3.068	0.361
0.301	-0.52	-0.127	-0.687	--	0.347	--	-0.127	0.175	0.324
0.164	--	0.552	0.425	0.55	0.435	--	0.122	0.199	0.367
0.259	-0.176	-0.035	-0.333	--	0.398	--	0.053	0.106	0.340
0.217	--	0.419	0.188	0.686	0.271	--	0.096	0.262	0.260
0.188	-0.606	-0.394	-0.875	--	0.131	--	-0.185	-0.054	0.116
-0.061	--	0.331	0.247	0.617	0.289	--	0.112	0.268	0.279
0.402	-0.465	-0.262	-0.523	--	0.474	--	0.207	0.176	0.463
0.321	--	0.524	0.743	0.875	0.514	--	0.650	0.575	0.695
0.024	-0.413	-0.127	-0.397	--	0.087	--	0.048	-0.372	-0.028
0.109	--	0.195	0.099	0.315	0.124	--	-0.013	0.133	0.015
-0.028	-0.091	-0.085	-0.209	--	0.064	--	0.099	-0.254	0.088
0.110	--	0.308	0.097	0.212	0.269	--	0.135	0.209	-0.056
0.253	-0.112	-0.141	-0.383	--	0.153	--	-0.063	-0.017	0.177
0.133	--	0.254	0.062	0.465	0.288	--	0.076	0.237	0.092
0.010	-0.255	-0.222	-0.494	--	-0.083	--	-0.155	-0.317	0.018
-0.023	--	0.127	-0.073	0.942	0.302	--	-0.038	0.105	-0.004
-1.400	-2.406	-2.922	-2.814	--	-1.896	--	-2.458	-2.522	-2.813
-1.929	--	-2.605	-2.667	-1.552	-2.422	--	-2.446	-2.598	1.684
-1.306	-2.377	-1.999	-2.150	--	-2.553	--	-1.899	-2.266	-2.331
-2.130	--	-2.781	-2.634	-1.963	-3.029	--	-2.516	-3.100	1.444
-1.003	-4.57	-4.987	-4.720	--	-4.891	--	-5.010	-4.790	-4.722
-1.885	--	-5.091	-4.952	-1.475	-4.897	--	-5.026	-4.225	2.541
-2.639	-7.58	-6.713	-7.235	--	-7.460	--	-6.569	-7.388	-7.559
-3.347	--	-8.072	-7.194	-2.684	-7.720	--	-7.305	-7.861	1.293
-0.344	-0.482	-0.608	-0.801	--	-0.895	--	0.001	-1.181	-0.845
-1.086	--	-2.279	-1.616	-1.217	-2.138	--	-2.058	-1.480	1.206
0.348	-3.996	-4.816	-4.578	--	-3.400	--	-4.313	-4.242	-0.797
0.403	--	-3.846	-4.265	-0.293	-3.078	--	-3.601	-3.059	0.350
0.108	-2.927	-3.136	-2.987	--	-2.834	--	-3.086	-3.143	-0.804
0.241	--	-2.573	-4.145	-0.433	-2.481	--	-2.294	-2.197	0.417
0.815	-0.741	-1.341	-1.536	--	-1.429	--	-1.555	-1.430	-0.411
0.586	--	-0.683	-0.783	0.763	-0.464	--	-0.886	-0.496	0.431

0.800	-1.723	-3.529	-2.906	--	-4.727	--	-2.656	-3.634	-1.380
-0.036	--	-3.516	-3.296	0.203	-3.926	--	-3.489	-4.283	-0.221
1.285	-0.782	-3.643	-3.025	--	-2.151	--	-2.495	-2.621	-0.755
1.095	--	-2.947	-3.237	0.984	-1.975	--	-2.828	-1.088	1.342
1.280	-0.333	-5.261	-5.269	--	-5.505	--	-5.283	-5.363	-0.612
0.848	--	-3.898	-3.530	0.427	-4.005	--	-2.652	-3.305	0.694
2.079	-3.906	-6.157	-5.753	--	-6.136	--	-6.334	-5.885	0.148
2.158	--	-5.755	-5.367	1.814	-4.854	--	-5.773	-5.013	1.745
1.069	-4.96	-5.609	-5.389	--	-5.374	--	-5.427	-5.473	-0.919
0.908	--	-5.859	-5.438	0.721	-5.269	--	-5.815	-5.504	1.257
1.253	-5.08	-6.222	-6.006	--	-5.872	--	-5.637	-5.830	-2.165
0.502	--	-5.450	-6.441	0.502	-5.783	--	-5.538	-6.480	0.206
0.073	-5.157	-6.612	-7.090	--	-7.042	--	-6.693	-7.125	-2.206
-0.308	--	-8.070	-7.409	-0.795	-7.337	--	-8.069	-7.031	-0.320
0.193	-5.979	-5.628	-5.852	--	-5.681	--	-5.834	-6.149	-1.117
1.520	--	-5.418	-5.549	-0.162	-5.651	--	-3.979	-4.997	0.359
-1.284	-8.008	-7.811	-8.029	--	-6.327	--	-7.732	-7.898	-2.636
-1.358	--	-6.826	-5.640	-2.446	-6.612	--	-6.609	-6.711	1.276
0.416	0.899	-2.390	-2.195	--	-0.389	--	-0.350	-0.854	-0.297
0.227	--	-1.421	-1.996	0.601	-0.150	--	-0.222	0.675	1.032
-0.739	0.826	4.904	2.040	--	-2.847	--	-0.988	-1.190	-1.301
1.612	--	-1.806	2.465	-0.762	-2.336	--	-2.147	-1.351	-2.301
1.476	1.309	6.398	5.461	--	-0.851	--	0.810	2.529	1.038
6.300	--	0.737	3.601	3.911	1.298	--	2.004	3.222	2.879
0.760	1.981	3.090	2.714	--	1.703	--	1.185	1.422	2.821
0.777	--	1.148	1.628	-0.1	1.216	--	1.154	0.464	1.850
-1.360	0.801	2.251	1.763	--	-0.406	--	1.328	0.630	1.428
1.061	--	-0.571	1.309	0.766	-2.200	--	0.371	-3.455	0.140
-0.158	0.335	1.488	1.478	--	0.016	--	1.135	0.616	0.996
0.743	--	-0.035	0.955	0.602	-0.611	--	0.294	-0.311	0.306
-0.039	-0.58	0.124	1.225	--	-0.979	--	0.299	-0.993	-0.476
0.570	--	-1.277	1.301	1.003	-3.491	--	0.078	-5.335	-2.890
-0.919	0.965	1.274	0.522	--	0.637	--	0.194	0.012	0.212
0.587	--	0.378	0.038	0.754	0.245	--	-0.033	1.239	-1.022
-2.782	0.947	1.805	2.875	--	-3.948	--	-0.403	0.498	0.025
0.942	--	-3.279	2.218	-0.133	-3.434	--	-0.861	-3.164	-0.738
0.144	0.367	0.961	0.930	--	-0.553	--	0.146	0.256	0.359
0.592	--	-0.294	0.682	0.47	-0.165	--	0.229	0.030	0.164
0.384	0.848	2.819	2.213	--	-0.278	--	1.826	1.268	2.429
1.404	--	-0.835	1.521	0.901	-0.022	--	0.805	-0.270	1.426
-0.270	0.748	3.839	2.555	--	-0.085	--	2.355	1.545	2.838
0.516	--	-0.423	2.223	1.153	-0.878	--	1.450	-0.519	1.602
-0.025	1.09	1.470	1.088	--	-0.067	--	1.964	1.564	1.372
0.550	--	-0.130	0.695	0.251	-0.696	--	0.681	-0.512	0.330
-0.554	1.383	2.898	2.721	--	-0.927	--	1.632	1.729	1.626
0.708	--	0.183	1.667	0.234	-0.539	--	1.026	-0.466	0.704
-0.188	1.443	2.925	2.688	--	-0.230	--	1.880	1.801	2.375

0.904	--	0.183	1.825	0.736	-0.163	--	1.090	-0.001	1.019
-0.477	1.262	3.171	2.576	--	-0.326	--	2.009	1.858	1.840
0.424	--	0.198	1.947	0.406	-0.556	--	1.350	-0.204	1.226
-0.171	1.187	1.705	1.542	--	-2.633	--	0.493	0.951	0.643
0.617	--	-0.173	1.066	0.142	-0.775	--	0.529	-0.464	0.372
2.092	1.251	0.748	0.038	--	1.618	--	2.118	-0.052	1.085
4.424	--	0.299	-1.162	2.459	0.974	--	2.230	2.438	1.372
0.846	0.283	0.163	0.039	--	1.426	--	0.861	0.722	0.697
0.411	--	0.540	0.959	0.791	1.129	--	0.964	1.637	1.326
0.418	0.215	0.389	0.129	--	-0.051	--	0.390	-0.121	0.285
-0.043	--	-0.125	-0.279	0.057	-0.629	--	0.490	-0.105	-0.021
1.582	3.153	1.473	0.043	--	0.299	--	1.226	0.110	1.703
0.647	--	0.262	0.205	3.341	0.064	--	1.482	1.676	2.195
0.121	-1.489	0.085	-0.103	--	-0.365	--	0.267	-0.270	-0.266
0.314	--	-1.491	-0.081	0.527	0.072	--	0.013	0.467	0.295
0.288	-0.009	-0.060	-0.227	--	0.150	--	-0.038	-0.065	0.374
0.096	--	0.009	0.058	0	0.074	--	-0.003	-0.009	-0.089
0.000	0.519	0.000	0.000	--	0.000	--	0.000	0.000	0.283
0.290	--	0.212	-0.150	-0.046	-0.361	--	0.342	-0.174	0.062
0.093	-0.166	0.267	-0.203	--	-0.055	--	0.098	0.178	-0.085
0.095	--	0.178	0.173	2.947	-2.100	--	0.195	-1.233	0.146
0.238	-0.211	-0.065	-0.453	--	0.053	--	-0.146	-0.400	-0.371
0.086	--	0.141	0.025	-1.85	-0.801	--	0.231	-1.439	0.440
0.289	0.659	1.092	1.482	--	-0.391	--	0.798	1.496	1.372
0.249	--	-0.078	-1.401	0.366	0.637	--	1.577	0.125	-0.112
0.574	2.28	1.178	0.000	--	0.400	--	0.088	0.476	0.482
0.200	--	0.353	-0.895	-0.077	0.982	--	-0.514	1.445	1.265
0.951	2.421	0.614	1.949	--	0.622	--	0.659	0.711	0.618
0.746	--	0.893	-0.478	1.023	1.576	--	-0.068	2.050	1.622
1.815	2.464	1.929	0.503	--	0.388	--	0.348	1.643	0.808
0.271	--	0.704	-2.752	0.321	1.037	--	-0.607	1.187	-0.619
1.417	2.296	1.081	0.682	--	0.681	--	0.861	0.172	0.280
0.993	--	0.350	-1.439	0.412	1.019	--	-0.215	1.248	-0.449
1.714	2.555	1.337	1.687	--	0.965	--	1.521	1.219	1.968
2.306	--	2.008	-0.043	2.917	2.261	--	1.175	2.900	-0.920
2.210	1.965	1.357	1.312	--	1.319	--	0.691	0.744	1.002
1.455	--	0.492	-0.434	1.517	1.282	--	0.201	1.545	0.577
0.303	0.085	0.317	-0.285	--	0.102	--	-0.310	-0.186	-0.062
0.208	--	-0.080	-0.375	0.213	0.342	--	-0.019	0.295	0.104
0.248	0.216	-0.001	-0.249	--	-0.062	--	-0.122	-0.274	-0.130
0.151	--	0.092	-0.535	0.16	0.275	--	-0.169	0.253	-0.196
1.088	0.459	0.659	0.555	--	0.129	--	0.105	0.296	0.224
0.919	--	0.115	-0.859	1.129	0.428	--	-0.509	0.766	0.043

38	39	40	41	42	43	44	45	46	48
--	121.80	--	91.80	97.80	154.80	145.80	--	141.40	155.20
119.80	114.40	--	111.00	89.60	183.20	172.40	82.60	146.40	157.60
--	14.30	--	14.90	15.90	17.50	17.80	--	13.60	15.70
15.70	--	--	14.10	13.70	14.80	15.30	13.60	13.80	15.10
13.66	12.82	13.96	17.98	14.28	16.18	13.74	14.90	16.10	15.86
--	19.12	--	18.59	19.69	17.90	17.52	--	18.58	22.87
22.65	21.68	--	21.35	22.71	25.40	24.77	20.51	18.76	24.10
--	24.08	--	24.53	27.73	24.76	23.83	--	22.79	32.14
33.17	26.10	--	26.12	28.10	32.58	28.22	24.23	23.33	32.06
--	83.93	--	89.18	79.97	80.60	81.73	--	89.07	68.38
70.60	87.38	--	83.03	81.38	71.84	80.77	86.75	90.07	72.91
--	14.00	--	16.00	28.00	62.00	62.00	--	21.00	42.00
37.00	9.00	--	13.00	20.00	48.00	48.00	16.00	16.00	37.00
168.00	134.00	124.00	134.00	148.00	158.00	175.00	134.00	134.00	158.00
--	6.08	--	6.43	3.43	33.75	28.95	--	9.25	19.25
13.77	7.55	--	3.88	1.99	38.00	39.67	4.89	6.48	17.75
9.22	6.37	30.20	10.59	3.92	18.93	22.56	16.48	17.75	28.44
1	1	--	1	1	2	2	1	1	2
1	1	--	1	1	2	2	1	--	2
1	0	--	0	0	2	2	0	0	2
--	-0.281	--	-0.243	-0.125	0.090	0.152	--	-0.372	-0.238
0.558	-0.189	--	0.096	0.241	0.228	0.156	-0.251	-0.261	0.255
--	0.089	--	-0.393	0.199	0.013	-0.117	--	-0.007	0.003
0.364	-0.165	--	-0.168	0.203	0.173	0.059	0.059	0.073	0.154
--	-0.175	--	-1.427	-1.664	0.317	0.181	--	-0.346	-0.439
1.126	-0.280	--	0.049	-0.053	1.039	0.648	0.106	-0.116	0.189
--	0.159	--	0.349	0.190	-0.060	0.016	--	-2.361	-0.306
3.732	0.765	--	-1.860	0.633	3.754	0.925	-0.139	-2.795	-1.742
--	0.539	--	0.122	0.167	0.140	-0.102	--	0.253	0.211
0.276	0.376	--	0.463	0.556	0.270	0.411	0.506	0.333	0.499
--	0.665	--	0.076	0.118	0.207	0.067	--	0.150	0.093
0.152	0.237	--	0.509	0.553	0.113	0.289	0.473	0.199	0.306
--	0.410	--	-0.008	-0.074	-0.026	-0.092	--	0.043	-0.135
0.185	0.176	--	0.268	0.395	0.193	0.303	0.35	0.228	0.264
--	0.535	--	0.055	-0.138	-0.056	-0.108	--	0.051	-0.091
0.171	0.205	--	0.258	0.461	0.078	0.209	0.384	0.196	0.233
--	0.278	--	-0.088	-0.141	-0.239	-0.255	--	-0.056	-0.247
0.188	0.122	--	0.231	0.420	0.093	0.220	0.325	0.144	0.240
--	0.523	--	0.176	0.002	-0.216	-0.200	--	0.188	-0.134
0.348	0.230	--	0.393	0.410	0.219	0.377	0.445	0.320	0.353
--	0.324	--	-0.003	-0.122	-0.049	-0.024	--	0.001	-0.162
0.117	0.172	--	0.369	0.414	0.073	0.234	0.41	0.108	0.238
--	0.077	--	-0.528	-1.038	-0.335	-0.353	--	-0.362	-0.530
-0.61	-0.212	--	0.332	0.302	-0.263	-0.674	0.119	-0.381	-0.102
--	1.096	--	0.433	0.082	0.197	-0.025	--	0.264	0.140

0.414	0.244	--	-0.625	0.535	0.235	0.391	0.552	0.409	0.440
--	2.060	--	1.520	0.893	-1.358	-1.214	--	0.269	0.076
1.261	-0.870	--	-1.548	1.257	1.345	2.452	2.658	1.747	0.353
--	-0.503	--	0.611	-1.492	-2.644	-2.770	--	-0.344	-3.638
-1.275	-0.319	--	-0.204	0.994	-1.847	-1.486	0.162	0.310	-1.723
--	-0.314	--	0.800	0.088	-0.787	0.261	--	0.243	0.333
0.605	1.011	--	1.268	1.000	0.859	0.923	0.186	0.316	1.168
--	-0.787	--	2.382	0.251	-1.139	0.708	--	0.441	0.331
-1.716	1.443	--	1.742	0.961	1.296	1.216	0.205	0.438	1.732
--	-3.426	--	3.390	0.262	-6.881	3.571	--	0.265	-0.012
3.488	3.434	--	3.524	3.481	3.313	3.538	-3.804	3.464	7.080
--	0.349	--	0.216	0.029	-0.235	2.933	--	0.398	-0.172
1.09	2.621	--	2.909	1.936	2.154	1.648	0.741	0.969	2.533
--	0.292	--	0.419	0.308	-0.186	0.333	--	1.090	0.017
1.208	2.574	--	3.197	1.281	2.969	2.576	0.98	1.246	2.779
--	-0.047	--	0.175	0.010	0.385	-0.019	--	0.503	0.319
0.244	0.079	--	0.050	0.297	0.373	0.136	0.485	-0.214	0.362
--	0.280	--	0.143	0.260	0.214	0.003	--	0.421	0.352
0.192	0.178	--	0.160	0.288	0.139	0.205	0.326	-0.042	0.303
--	0.067	--	0.074	-0.119	0.140	-0.050	--	0.276	0.054
0.178	0.163	--	0.207	0.237	0.262	0.318	0.278	-0.255	0.526
--	0.995	--	0.298	0.267	0.312	-0.237	--	0.253	0.334
0.516	0.407	--	0.485	0.591	0.498	0.748	0.737	0.013	0.746
--	0.182	--	0.403	0.032	-0.049	0.112	--	0.054	0.031
0.058	-0.064	--	0.025	0.210	0.242	0.152	0.126	-0.124	0.136
--	0.337	--	0.022	-0.007	0.128	0.098	--	0.045	0.007
0.204	0.008	--	0.005	0.207	0.126	0.098	0.041	-0.061	0.121
--	0.563	--	0.121	0.242	0.074	0.098	--	0.319	0.157
0.097	0.276	--	0.249	0.347	0.243	0.119	0.251	-0.013	0.269
--	0.627	--	-0.038	-0.037	0.003	-0.081	--	0.104	0.047
0.235	-0.017	--	0.000	0.369	-0.051	-0.023	0.15	-0.408	0.106
--	1.928	--	-0.284	-1.496	-2.778	-2.662	--	0.430	-2.566
-2.313	0.843	--	1.442	-2.147	-2.449	-2.548	-0.349	1.577	-2.664
--	1.598	--	-0.614	-1.793	-2.220	-1.987	--	0.325	-2.035
-2.53	0.204	--	0.569	-2.925	-3.082	-3.151	-0.556	1.456	-2.956
--	2.176	--	-0.035	-4.435	-4.893	-5.040	--	1.034	-5.149
-3.369	1.088	--	2.084	-5.054	-3.158	-4.200	-0.05	2.165	-4.159
--	1.778	--	-1.366	-6.629	-7.051	-7.265	--	0.301	-6.338
-5.943	0.138	--	0.497	-6.832	-7.790	-7.492	-0.943	1.631	-6.058
--	1.549	--	0.638	-0.761	-0.566	-0.528	--	0.205	-0.838
-1.603	0.928	--	1.160	-1.514	-1.859	-1.807	-0.428	1.478	-1.736
--	1.911	--	1.683	-2.960	-3.526	-3.362	--	1.299	-1.319
-1.532	1.096	--	1.465	-3.113	-1.570	-1.694	-0.387	1.239	-1.903
--	1.988	--	1.636	-2.466	-2.818	-2.580	--	1.257	-1.248
-1.671	1.173	--	1.507	-2.402	-1.541	-1.559	-0.307	1.309	-1.595
--	2.228	--	2.009	-1.119	-0.652	-0.442	--	1.175	0.488
0.271	1.273	--	1.440	-0.763	0.007	0.089	0.457	1.053	0.595

--	2.479	--	2.154	-3.051	-2.329	-2.829	--	1.568	-0.923
-1.135	1.160	--	1.357	-3.508	-1.830	-1.727	0.459	1.753	-1.015
--	2.205	--	2.100	-2.889	-1.005	-1.782	--	1.577	-0.376
-0.251	1.634	--	2.418	-3.050	0.229	0.374	0.585	1.320	0.574
--	2.172	--	1.982	-5.313	-0.871	-2.295	--	1.506	-0.081
-0.207	0.888	--	1.523	-5.001	0.007	-0.113	0.266	2.338	-0.025
--	2.452	--	3.132	-5.653	-2.495	-3.300	--	2.486	0.343
0.689	2.278	--	3.123	-5.488	0.986	0.742	0.977	1.788	1.580
--	1.823	--	1.809	-5.211	-5.497	-5.561	--	1.249	-0.343
-0.215	1.424	--	2.226	-5.576	-0.632	-0.493	0.642	1.406	0.331
--	2.813	--	1.736	-5.397	-4.153	-4.311	--	1.162	-1.382
-1.903	1.594	--	1.321	-6.105	-1.855	-1.545	0.815	1.399	-1.333
--	1.504	--	0.370	-7.007	-5.014	-4.381	--	-0.182	-1.733
-1.807	-0.268	--	-2.044	-7.408	-2.860	-2.516	-0.301	-1.543	-2.266
--	0.387	--	0.571	-5.740	-6.134	-5.813	--	0.234	-0.972
-1.847	-0.398	--	0.226	-5.846	-2.817	-4.187	0.066	0.920	-2.124
--	-0.195	--	-1.371	-6.542	-8.364	-7.677	--	-0.932	-3.278
-3.076	-1.739	--	-0.375	-7.072	-4.045	-4.030	-1.209	0.892	-3.906
--	0.797	--	0.557	-2.322	0.715	-0.131	--	0.542	-0.453
0.058	0.749	--	0.854	-1.523	1.259	1.311	-0.487	0.970	0.306
--	-2.042	--	-1.324	1.335	-1.180	-1.546	--	-0.289	-1.566
4.991	-1.328	--	-0.511	2.373	-1.600	-1.956	-0.787	0.013	-1.811
--	3.554	--	3.648	4.837	2.916	1.238	--	2.360	0.891
7.184	2.109	--	3.383	6.146	2.394	1.158	2.862	1.987	0.349
--	1.751	--	1.102	2.110	1.986	1.631	--	1.541	2.567
1.494	-0.433	--	-0.254	0.845	0.788	0.129	0.939	-1.006	0.413
--	-0.532	--	-0.454	2.810	-0.293	0.488	--	-0.133	0.844
0.193	1.280	--	0.966	2.642	0.550	0.822	0.121	-1.580	-0.027
--	0.554	--	-1.026	1.302	-1.050	-0.691	--	0.063	0.619
-0.076	0.716	--	0.691	1.507	0.771	0.761	-0.079	0.239	0.053
--	1.224	--	-0.789	1.978	-5.284	-5.182	--	-0.071	-0.251
0.032	1.842	--	1.155	2.540	-0.756	-0.534	-0.01	0.370	-0.290
--	0.262	--	-0.847	0.946	-0.830	-0.940	--	-0.555	-0.060
-1.103	0.229	--	-0.332	0.467	0.916	1.159	0.389	0.416	-0.105
--	-3.928	--	-1.509	-0.004	-3.775	-0.506	--	-1.355	0.306
3.509	-1.427	--	0.848	1.663	-0.236	-0.252	-2.893	0.894	0.706
--	0.244	--	0.202	0.628	-0.929	-0.190	--	0.193	0.574
0.951	0.392	--	0.153	0.497	-0.017	-0.100	-0.15	0.077	0.321
--	0.478	--	-0.416	1.481	0.863	0.915	--	0.803	2.628
1.046	0.642	--	0.439	1.669	1.115	1.261	0.39	-0.601	0.860
--	0.561	--	-0.507	1.147	1.049	1.377	--	0.906	3.216
1.201	0.141	--	0.582	1.617	1.524	1.518	-0.055	-0.353	1.208
--	0.659	--	0.054	1.302	1.189	1.333	--	0.681	1.396
0.603	-0.037	--	0.306	0.269	0.400	0.476	-0.056	-0.370	0.269
--	-0.064	--	-0.308	0.948	0.718	1.287	--	0.147	1.976
2.007	0.083	--	0.267	1.131	1.146	1.008	-0.246	-0.396	1.037
--	0.387	--	0.121	1.050	1.042	1.275	--	0.744	2.345

1.868	0.394	--	0.556	1.360	1.521	1.501	-0.061	-0.339	1.233
--	0.066	--	-0.272	0.761	0.497	0.387	--	0.166	2.287
2.653	-0.314	--	0.452	0.986	1.069	0.794	-0.032	-0.316	1.001
--	-0.505	--	-1.002	0.531	0.790	1.270	--	-0.302	0.748
1.435	-0.105	--	-0.165	0.666	1.076	0.583	-0.663	-0.622	0.577
--	3.629	--	2.602	1.813	0.952	0.194	--	1.485	1.211
-0.143	1.395	--	2.300	0.463	0.968	0.055	3.985	3.360	0.452
--	1.493	--	0.615	1.635	1.060	0.136	--	0.419	0.631
-0.052	-0.536	--	0.479	1.106	0.554	0.389	0.515	-0.354	-0.123
--	0.363	--	-0.206	0.900	0.205	0.015	--	-0.088	-0.006
-0.453	-0.761	--	-0.697	-0.120	-0.493	-0.839	-0.1	-0.084	-0.668
--	2.991	--	2.590	3.829	1.470	0.134	--	2.674	2.028
1.122	0.460	--	2.111	2.008	2.623	1.313	2.161	3.004	0.644
--	0.658	--	0.406	0.223	0.183	0.106	--	0.282	-0.068
-0.161	0.254	--	0.522	0.137	-1.016	-1.338	0.249	0.361	0.278
--	0.323	--	0.252	0.618	0.215	-0.043	--	0.194	0.129
0.03	0.000	--	0.000	0.158	-1.391	-1.388	-0.049	0.000	-0.031
--	0.000	--	0.000	0.000	0.000	0.000	--	0.471	0.000
-0.301	0.143	--	-0.101	0.156	-0.300	-0.175	0.062	0.109	-0.187
--	0.276	--	0.528	0.164	0.407	0.202	--	0.008	0.438
2.094	-2.734	--	-3.584	-2.593	-2.970	-1.336	0.152	-0.418	-0.598
--	0.378	--	-1.275	-1.637	0.055	0.127	--	0.101	-0.123
-1.475	0.336	--	0.275	-1.271	-1.351	-1.885	0.352	0.145	-0.213
--	0.063	--	0.195	0.736	0.090	-0.217	--	-0.228	0.519
-1.419	0.149	--	0.396	-0.141	0.543	0.097	0.114	1.434	0.192
--	0.148	--	0.375	0.070	2.875	2.598	--	0.185	-0.060
-0.746	-0.209	--	-1.145	-1.061	-0.927	-0.824	0.351	-0.588	-0.609
--	2.483	--	2.737	3.136	2.710	1.647	--	0.684	0.687
-0.128	0.703	--	-0.080	-0.655	-0.368	-0.177	0.93	-0.248	0.334
--	0.622	--	1.532	1.132	2.193	1.821	--	0.715	0.067
-1.352	-0.480	--	-2.809	-2.418	-1.166	-2.667	0.358	-0.748	-1.296
--	1.820	--	1.983	1.520	1.306	0.906	--	1.082	-0.273
-0.651	0.394	--	-0.567	-0.770	-0.895	-1.047	0.289	-0.178	-0.855
--	2.722	--	2.966	2.183	2.419	1.587	--	2.930	0.629
-0.203	2.146	--	1.019	0.765	1.466	1.688	1.063	0.680	1.473
--	2.462	--	2.357	1.962	1.796	1.545	--	1.473	1.311
-0.437	1.457	--	0.644	0.330	0.172	0.288	0.668	0.019	0.282
--	0.094	--	0.135	0.271	0.467	0.600	--	0.207	0.035
-1.417	-0.081	--	-0.897	-0.510	-1.086	-0.730	0.356	-0.634	-0.524
--	0.176	--	0.097	0.183	0.192	0.158	--	0.065	-0.247
-0.835	0.147	--	-0.319	-0.407	-0.750	-0.452	0.324	-0.158	-0.362
--	1.940	--	1.695	1.020	0.627	0.533	--	0.846	0.816
-0.251	1.750	--	1.418	0.617	0.723	0.970	0.902	0.484	0.443

Hibrid number									
**49**	**50**	**52**	**54**	**55**	**56**	**57**	**58**	**60**	**61**
--	136.60	119.40	85.40	80.20	86.80	145.40	109.20	185.40	131.40
139.80	133.60	113.80	88.60	78.60	59.60	135.20	140.00	143.80	51.80
--	17.90	18.80	14.40	15.30	--	18.50	17.00	13.30	13.80
15.10	15.50	17.00	14.50	13.50	14.20	13.80	14.40	14.50	12.70
17.30	--	--	14.82	--	13.58	16.02	17.28	17.42	--
--	23.20	22.24	19.81	21.10	22.43	21.89	22.48	18.62	21.68
26.58	26.07	24.10	24.47	24.02	25.85	25.74	25.01	23.25	27.89
--	31.49	23.98	22.75	24.89	28.22	23.04	36.34	24.69	27.00
40.90	40.32	27.57	27.38	24.92	28.64	29.48	37.14	28.86	32.95
--	76.30	88.78	93.06	91.36	76.99	89.41	61.71	84.71	80.08
65.71	65.07	82.03	83.83	88.70	79.66	82.34	69.98	83.91	72.65
--	58.00	48.00	6.00	16.00	6.00	42.00	42.00	14.00	14.00
27.00	48.00	41.00	9.00	13.00	6.00	30.00	30.00	9.00	13.00
144.00	172.00	--	134.00	--	124.00	158.00	152.00	134.00	--
--	29.42	45.33	29.17	13.59	3.73	54.00	16.83	25.75	5.58
18.58	25.67	20.70	13.75	18.78	2.97	--	--	21.67	2.42
16.67	17.75	--	8.92	--	15.69	27.75	25.20	22.07	--
2	2	2	--	1	1	2	2	2	2
2	2	2	--	1	1	2	2	2	2
2	2	--	2	--	0	2	2	2	--
--	0.058	-0.363	-0.308	-0.287	-0.272	-0.434	0.142	-0.032	0.870
0.252	0.492	-0.196	0.218	-0.347	0.177	0.306	0.542	-0.145	0.195
--	-0.070	-0.418	-0.081	-0.367	-0.240	-0.184	-0.108	-0.033	0.504
0.294	0.538	-0.118	0.099	-0.157	0.206	0.007	0.289	0.099	-0.006
--	-0.156	0.105	-1.532	-1.190	-2.959	-0.125	-1.581	0.011	-1.933
0.766	1.290	0.935	0.750	-0.450	0.577	0.627	0.949	0.802	0.394
--	0.279	-0.180	-0.295	-0.164	-0.349	-0.721	-0.187	-0.391	0.562
2.785	2.670	4.268	5.703	-2.721	0.466	0.805	3.073	2.938	3.973
--	-0.157	-0.472	0.093	-0.075	0.160	-0.244	-0.583	0.499	0.551
0.494	0.187	0.117	0.567	0.130	0.120	0.332	0.502	0.590	0.432
--	-0.392	-0.324	0.237	0.167	0.002	0.233	-0.697	0.183	0.830
0.51	0.103	0.081	0.318	0.165	0.186	0.272	0.472	0.273	0.413
--	-0.436	-0.672	-0.006	-0.022	-0.157	0.036	-0.831	-0.051	0.146
0.248	0.197	-0.099	0.286	0.090	0.047	0.273	0.274	0.261	0.295
--	-0.564	-0.602	0.007	-0.104	-0.158	0.141	-0.897	-0.118	0.232
0.22	0.256	-0.113	0.256	0.051	0.080	0.124	0.219	0.265	0.330
--	-0.459	-0.612	-0.019	-0.258	-0.245	0.033	-0.880	-0.025	0.147
0.234	0.159	-0.189	0.286	0.036	0.032	0.166	0.180	0.234	0.285
--	-0.340	-0.384	0.139	-0.232	-0.065	0.212	-0.799	0.129	0.384
0.404	0.342	0.005	0.377	0.257	0.167	0.278	0.368	0.461	0.268
--	-0.511	-0.546	-0.036	0.001	-0.372	0.230	-0.829	-0.155	0.166
0.265	0.156	-0.106	0.196	0.039	0.043	0.156	0.279	0.159	0.194
--	-0.370	-0.605	-0.224	-0.542	-0.470	-0.942	-1.388	0.215	-0.020
-0.159	-0.595	-0.338	-0.167	-0.227	-0.386	-0.025	0.024	0.197	-0.210
--	-0.341	-0.466	0.363	0.303	0.083	0.631	-0.834	0.295	0.675

0.637	0.364	0.093	0.468	0.426	0.285	0.214	0.476	0.578	0.459
--	-0.256	-0.400	0.196	-1.272	1.345	1.515	-0.566	2.780	1.461
1.518	1.069	1.260	-1.260	1.257	0.078	2.431	3.828	2.571	2.216
--	-1.852	-1.387	-1.082	0.229	-2.910	0.714	-3.462	-0.242	-2.491
-0.216	-0.912	-1.360	0.922	0.520	0.035	-0.643	0.679	0.156	-2.060
--	0.552	-0.793	-0.990	-0.899	-1.138	-1.665	-0.481	-0.393	0.705
-0.014	-0.101	1.133	-0.290	0.511	0.455	0.717	0.250	0.119	-0.312
--	1.024	-5.210	-1.440	-3.492	-3.761	-8.626	-0.013	-0.517	0.654
-0.035	-0.218	1.860	-0.102	0.689	0.748	1.321	0.353	0.384	-0.355
--	3.510	-10.350	-10.397	-3.488	-7.144	-10.609	-0.258	-10.515	3.901
3.545	-0.032	7.121	-0.065	-10.844	3.485	3.351	-3.762	-0.166	5.299
--	0.312	-0.303	-0.183	-0.015	-0.114	-0.122	-0.034	-0.122	0.402
-0.029	0.012	2.445	0.103	1.376	1.188	1.973	0.605	1.200	-0.081
--	0.398	-0.324	-0.277	0.095	-0.317	-0.159	0.152	-0.269	0.584
0.32	-0.250	2.539	0.879	1.389	1.425	2.871	1.302	1.331	0.614
--	-0.061	-0.966	0.215	0.108	0.459	-1.171	0.086	0.490	0.344
0.239	0.012	0.032	0.348	0.007	-0.058	0.292	0.297	0.058	0.152
--	-0.070	-0.552	0.255	0.208	0.288	-0.663	-0.258	0.354	0.750
0.177	0.062	-0.058	0.109	0.011	0.121	0.197	0.294	0.263	0.090
--	-0.403	-0.177	0.256	0.107	0.039	-0.332	-0.300	0.211	0.243
0.191	-0.147	-0.023	0.309	-0.006	-0.233	0.356	0.323	-0.085	0.161
--	-0.332	-0.576	0.601	0.275	0.505	0.246	-0.157	0.676	0.384
0.545	0.310	0.266	0.576	0.391	0.137	0.530	0.648	0.662	0.624
--	-0.052	-0.482	-0.186	-0.088	-0.208	-0.160	-0.204	-0.141	0.289
0.049	0.123	-0.153	-0.027	-0.237	-0.044	0.050	0.007	-0.064	0.048
--	-0.004	-0.328	-0.036	-0.016	-0.126	0.202	-0.183	-0.158	0.216
0.093	0.202	-0.067	0.127	-0.060	0.154	0.172	0.120	0.078	0.020
--	-0.141	-0.529	-0.003	0.151	0.046	-0.220	-0.258	0.069	0.683
0.135	0.010	0.016	0.266	0.000	0.081	0.219	0.176	0.035	0.028
--	-0.303	-0.571	-0.069	-0.056	-0.252	0.014	-0.297	-0.202	0.343
-0.032	0.004	-0.422	-0.112	-0.213	-0.056	-0.042	-0.112	-0.294	0.025
--	-2.191	-2.969	-2.256	-2.744	-2.137	-2.826	-2.940	-1.233	-1.780
-1.678	-2.406	-2.663	-1.416	-1.122	-2.573	-2.847	-2.208	-2.546	-2.627
--	-2.423	-2.552	-2.124	-2.169	-2.243	-2.340	-2.472	-1.293	-1.866
-1.874	-2.602	-3.109	-1.859	-1.451	-2.932	-3.566	-2.469	-2.907	-3.088
--	-4.505	-4.797	-2.551	-4.987	-2.349	-5.010	-4.955	-1.222	-4.478
-0.832	-4.489	-4.904	-1.119	-0.759	-4.918	-5.288	-2.474	-4.012	-4.110
--	-6.548	-8.082	-4.915	-6.146	-4.396	-7.352	-7.544	-1.860	-6.573
-4.223	-5.936	-6.943	-2.140	-1.771	-5.175	-8.611	-5.411	-4.697	-7.311
--	0.058	-1.001	-0.541	-0.405	-0.878	-0.648	-0.722	-0.280	0.259
-1.292	-1.047	-1.730	-0.999	-0.844	-1.464	-2.252	-1.513	-1.625	-1.899
--	-3.329	-3.646	0.299	-0.409	0.038	-5.352	-2.784	0.187	-3.003
-0.144	-1.640	-1.516	-0.045	-0.270	-1.013	-4.395	-0.598	-0.546	-4.170
--	-2.487	-3.115	0.153	-0.710	-0.169	-5.413	-2.639	0.085	-2.241
-0.243	-1.591	-1.512	-0.186	-0.187	-0.974	-3.207	-0.790	-0.615	-3.256
--	-0.311	-1.422	0.630	0.424	0.932	-1.703	-0.494	0.476	-0.863
0.565	0.019	0.162	1.095	0.695	0.275	-0.744	0.420	0.403	-1.141

--	-1.350	-3.048	0.179	-0.560	0.344	-4.047	-1.493	1.065	-3.121
0.166	-1.122	-1.314	-0.069	0.353	-0.463	-4.431	-0.661	-0.376	-4.047
--	-1.351	-1.207	0.772	0.360	1.074	-2.207	-2.198	0.516	-2.223
0.319	-0.636	0.330	0.918	1.135	0.078	-2.350	0.469	0.568	-2.986
--	-2.414	-2.638	0.280	0.009	0.654	-5.499	-4.097	0.562	-4.955
0.237	-0.369	-0.079	0.362	0.216	-0.152	-3.919	0.313	-1.284	-2.857
--	-2.587	-3.416	1.764	1.071	1.962	-6.334	-2.549	1.433	-5.487
1.098	0.014	1.144	1.643	2.094	1.151	-5.728	1.270	1.578	-6.150
--	-3.579	-5.329	0.419	-0.529	0.335	-5.427	-4.155	0.452	-5.205
0.64	-0.949	-0.305	0.573	0.653	-0.029	-5.889	0.560	0.106	-6.004
--	-4.229	-4.403	0.177	0.064	0.266	-6.099	-3.138	0.461	-5.171
-0.346	-2.272	-1.320	0.197	0.675	-0.414	-5.905	-1.325	-0.044	-4.851
--	-4.454	-4.762	-1.222	-1.208	-3.834	-6.584	-3.010	-0.829	-7.717
-2.271	-2.304	-2.727	-2.611	-1.219	-5.434	-8.563	-0.807	-3.297	-6.956
--	-5.003	-6.134	-1.031	-2.661	-1.261	-5.806	-4.026	-0.009	-5.617
1.757	-2.213	-3.845	-1.726	-0.688	-0.337	-5.980	0.166	-1.842	-2.990
--	-8.041	-7.721	-3.285	-4.434	-2.979	-8.022	-6.007	-1.363	-7.577
-1.198	-5.145	-5.473	-3.595	-2.102	-3.353	-7.340	-2.486	-4.392	-5.945
--	-0.029	0.538	-0.208	-0.519	-0.163	-0.909	-1.313	-0.566	-0.343
-0.055	0.310	1.503	-0.042	0.080	-0.485	-0.330	0.144	-0.083	-1.650
--	2.830	-1.920	-1.924	-2.191	-2.085	-1.899	5.516	-2.058	0.163
-1.383	1.742	-1.646	-0.971	-2.622	-0.648	-1.302	1.268	-2.013	0.538
--	4.579	-0.631	2.058	1.388	2.775	1.617	7.472	2.938	3.999
1.572	3.407	2.576	-2.431	1.436	3.053	0.303	3.174	3.372	6.340
--	1.950	-0.274	0.667	1.135	1.022	-1.043	2.007	1.679	1.783
0.928	0.231	0.240	0.432	0.468	-0.377	0.229	0.309	0.156	0.596
--	0.807	-4.891	-4.385	-4.308	-3.188	-4.539	0.906	1.209	-0.612
0.891	1.253	-2.218	1.336	-3.982	-0.369	-2.512	-0.716	1.576	0.510
--	-0.741	-1.859	-2.537	-2.206	-1.683	-3.383	0.687	0.975	1.034
1.203	1.051	0.399	1.075	-0.928	-0.088	-0.490	-0.399	1.091	0.404
--	-0.971	-5.399	-4.983	-5.107	-2.350	-5.111	0.175	1.080	1.896
0.863	1.177	-2.623	1.598	-0.780	-0.064	-3.176	-0.678	1.785	0.878
--	-0.424	-0.509	-3.264	-2.053	-2.444	-2.177	0.518	0.970	1.271
0.16	0.319	1.461	0.964	-0.347	-0.020	0.061	-0.613	1.283	0.107
--	2.514	-3.936	-3.995	-3.724	-3.915	-4.163	2.247	0.180	1.070
0.809	2.718	-2.889	0.607	-3.537	0.657	-0.629	2.016	0.164	1.154
--	0.563	-1.620	-0.557	-0.622	-0.169	-1.046	0.890	0.338	1.081
0.53	0.443	-0.611	0.500	-0.648	0.339	-0.076	0.661	0.449	0.088
--	1.048	-1.813	-0.531	-1.227	-0.393	-5.392	1.343	1.849	1.630
1.648	1.428	-0.012	1.474	-1.375	0.361	-0.149	0.675	1.455	0.756
--	0.933	-2.705	-1.354	-1.951	-0.257	-5.266	1.852	2.691	1.551
1.283	1.771	-0.476	1.483	-1.629	0.011	-0.161	0.751	2.069	0.739
--	1.286	-2.310	-0.514	-1.354	0.020	-4.634	0.693	1.448	1.484
0.736	0.632	-0.548	0.424	-2.059	-0.044	0.167	0.400	0.345	0.629
--	2.094	-1.221	-0.918	-1.106	-0.414	-2.750	2.410	0.650	0.801
1.416	1.604	0.240	0.546	-1.815	-0.032	0.484	1.344	0.718	0.243
--	1.986	-0.944	-0.395	-0.242	-0.041	-1.907	2.390	1.370	1.549

1.152	1.662	0.387	0.931	-0.785	0.259	0.643	1.443	1.234	0.551
--	1.849	-2.088	-1.259	-1.761	-0.722	-4.708	1.947	1.635	1.706
1.562	1.998	-0.391	0.804	-1.980	-0.352	0.756	2.482	0.873	0.809
--	1.432	-2.417	-0.768	-2.483	-0.681	-2.669	0.886	0.320	0.437
1.182	1.328	0.117	0.078	-1.464	-0.198	0.450	1.283	0.517	0.765
--	0.536	-1.428	1.949	1.681	2.431	1.684	-0.307	2.709	3.130
-0.021	1.320	1.052	2.205	4.519	1.512	-0.190	-0.755	4.394	4.042
--	-0.154	-0.597	1.149	1.823	1.225	0.681	-0.951	1.342	1.498
0	0.059	-0.017	0.385	0.761	0.281	0.948	-0.064	0.940	1.483
--	0.300	0.047	0.298	-0.022	0.293	-0.281	0.036	0.897	1.457
0.062	0.317	-0.880	-0.493	-0.471	-0.174	-0.980	-0.415	0.375	-0.176
--	1.059	-1.448	2.531	1.508	3.925	-1.370	0.419	4.677	4.963
2.732	1.609	2.059	1.579	1.681	1.348	1.349	2.496	3.743	0.988
--	0.097	-0.525	-0.273	-0.478	-0.120	-0.302	-0.322	0.226	0.414
-0.011	-0.146	0.206	0.213	-0.096	0.136	-1.166	-1.158	0.336	0.634
--	0.114	-0.341	0.438	0.224	0.207	-0.234	-1.553	0.130	0.500
0.012	0.025	0.000	0.091	-0.064	0.027	-0.002	-0.027	0.000	0.000
--	0.346	0.000	0.000	0.000	0.000	0.000	0.000	0.000	0.000
0.204	0.412	-0.448	-0.038	0.304	-0.044	-0.204	-0.237	-0.051	0.207
--	0.058	-3.237	0.039	0.276	0.029	0.361	-0.311	-0.050	0.596
-0.187	2.482	-3.461	0.370	-0.266	-2.474	-2.859	-0.174	-2.862	-0.066
--	-0.211	-0.392	0.384	0.182	0.225	-0.032	-0.407	0.338	-1.303
0.119	0.187	-1.543	0.241	-0.270	-0.014	-1.723	-0.094	-1.621	0.331
--	0.482	0.779	0.504	0.578	0.570	1.082	0.664	0.603	1.229
0.393	0.798	1.888	0.285	-0.004	0.208	0.146	-1.566	0.218	0.228
--	0.693	2.066	1.066	1.085	0.742	2.627	0.324	0.377	0.639
-0.962	-1.352	0.210	-0.264	0.406	0.080	-0.028	-1.183	-0.373	-0.369
--	1.013	2.197	1.822	1.594	1.269	2.838	0.314	0.468	2.940
-0.445	-1.296	1.072	0.658	1.166	0.543	0.897	-1.376	0.521	0.676
--	2.302	2.374	2.111	2.319	2.432	2.277	1.019	0.497	1.727
-2.635	-1.679	0.503	-0.689	0.870	0.574	-0.423	-1.366	-0.583	-0.438
--	2.006	1.556	2.415	2.275	2.597	2.083	0.329	0.997	2.434
-1.086	-0.675	0.350	0.224	1.167	1.227	-0.433	-0.818	0.474	0.524
--	2.738	1.567	2.820	2.588	2.893	1.980	0.851	2.062	3.258
-0.343	0.762	2.672	2.529	2.205	2.443	1.668	-1.586	2.699	1.015
--	1.430	0.959	2.495	2.405	2.869	1.630	0.430	1.645	2.713
-0.722	-0.414	0.307	1.667	1.350	0.982	0.160	-1.036	1.724	1.533
--	-0.131	-0.100	0.402	0.538	0.319	-0.114	-0.182	0.398	0.527
-1.752	-1.869	-0.426	-0.343	0.216	-0.151	-0.839	-2.349	-0.061	-0.492
--	0.134	0.152	0.175	0.346	0.097	0.003	-0.248	-0.070	0.270
-0.859	-1.075	0.187	-0.369	0.369	0.220	-0.332	-1.272	-0.092	-0.189
--	1.343	0.370	1.610	1.378	1.450	0.679	-0.311	0.073	1.386
-0.03	0.165	0.453	1.408	1.153	1.339	-0.102	-0.598	0.819	1.074

62	63	64	66	67	68	69	70	71	73
130.60	--	135.60	--	109.80	161.00	--	111.60	113.20	82.20
111.00	108.80	144.60	202.80	147.20	128.80	124.60	72.20	108.00	54.60
20.80	19.00	13.60	--	17.20	17.30	--	14.70	18.10	14.40
16.40	15.90	13.10	13.10	12.80	12.90	--	14.50	13.70	15.40
16.38	15.68	17.16	17.60	16.52	16.88	15.70	14.70	14.02	14.44
19.11	26.75	21.51	--	21.65	22.65	--	20.31	21.18	28.89
29.18	23.33	20.68	21.70	27.50	22.35	24.59	25.56	22.73	28.99
29.26	32.60	25.25	--	32.32	28.84	--	25.69	27.12	48.04
35.05	30.87	26.98	30.04	27.66	26.83	33.01	30.28	26.64	41.13
72.72	78.37	88.14	--	69.06	81.43	--	85.59	78.47	66.50
72.68	66.39	80.91	76.09	84.07	90.78	78.78	85.94	86.68	68.40
--	--	16.00	--	58.00	58.00	--	6.00	28.00	0.00
48.00	41.00	16.00	34.00	34.00	41.00	16.00	2.00	16.00	0.00
172.00	168.00	144.00	152.00	152.00	175.00	144.00	124.00	144.00	114.00
33.42	19.25	42.50	--	26.88	45.67	--	--	5.18	--
19.50	26.17	33.33	32.50	42.00	56.67	3.38	2.83	19.30	--
19.81	18.44	22.85	29.91	35.79	29.22	3.82	10.49	5.30	25.50
2	2	1	2	2	2	1	1	1	1
2	2	1	2	2	2	1	1	1	1
2	2	2	2	2	2	0	0	0	0
0.013	-0.035	-0.108	--	-0.209	0.366	--	-0.214	0.142	-0.412
-0.002	0.475	-0.235	0.176	-0.715	-0.151	--	-0.314	-0.304	-0.047
-0.455	-0.375	0.440	--	-0.634	0.546	--	-0.446	0.181	-0.248
0.017	0.170	0.016	0.218	-0.380	0.113	--	-0.232	-0.011	-0.190
-0.328	-0.287	-0.209	--	-0.242	0.074	--	-0.558	-1.070	-0.517
0.310	1.083	0.047	0.22	-0.525	0.045	--	0.313	-0.165	-0.479
-0.503	-0.255	0.441	--	0.000	0.479	--	-0.190	0.143	-0.048
-0.565	3.446	0.098	-1.121	0.022	5.609	--	2.676	-2.799	3.244
-0.188	-0.365	0.442	--	-0.793	-0.025	--	-0.363	0.243	0.365
-0.196	0.278	0.526	0.35	0.101	0.584	--	-0.031	0.305	-0.101
-0.281	-0.499	0.893	--	-0.745	0.001	--	-0.257	0.034	0.129
-0.227	0.096	0.540	0.266	-0.251	0.654	--	-0.172	0.055	-0.248
-0.831	-0.374	0.551	--	-0.607	0.082	--	-0.379	-0.094	0.048
-0.220	0.139	0.416	0.283	0.015	0.535	--	-0.216	0.073	-0.194
-0.802	-0.337	0.508	--	-0.608	0.001	--	-0.394	-0.136	-0.011
-0.298	0.038	0.343	0.288	-0.016	0.500	--	-0.229	0.023	-0.194
-0.928	-0.514	0.492	--	-0.817	0.039	--	-0.456	-0.121	-0.016
-0.274	0.107	0.334	0.247	-0.101	0.478	--	-0.294	0.012	-0.194
-0.676	-0.332	0.665	--	-0.507	-0.045	--	-0.372	0.017	0.142
-0.207	0.233	0.487	0.408	0.032	0.507	--	-0.108	0.185	-0.186
-0.703	-0.395	0.633	--	-0.840	-0.085	--	-0.357	-0.116	-0.052
-0.316	0.075	0.348	0.199	-0.218	0.427	--	-0.330	-0.008	-0.303
-0.908	-0.788	-0.072	--	-1.732	-0.451	--	-1.146	-0.192	-0.356
-0.211	-0.652	0.041	-0.122	-0.507	0.357	--	-0.396	-0.012	-0.355
-0.580	-0.240	1.125	--	-0.557	-0.174	--	-0.108	-0.022	0.507

-0.178	0.094	0.681	0.467	0.079	0.663	--	0.044	0.187	-0.178
-0.459	-0.251	1.980	--	-1.770	-0.311	--	-0.570	1.214	0.665
-0.351	1.183	1.881	-1.318	0.313	1.133	--	-0.917	1.034	-2.451
-1.043	-3.256	1.423	--	-3.676	-0.027	--	-2.040	-2.765	-1.989
0.022	-2.357	-0.869	-0.166	0.317	0.821	--	0.166	-0.835	-1.731
-0.434	0.993	0.402	--	-0.527	0.607	--	-1.142	0.579	0.002
-0.469	0.392	0.174	0.941	0.091	-0.122	--	-0.140	0.355	0.421
-0.485	2.624	-2.471	--	-0.786	0.954	--	-6.197	1.004	-0.673
-0.515	0.553	0.320	1.492	0.108	-0.115	--	-0.014	0.322	0.875
-3.878	-7.074	3.772	--	-0.870	10.233	--	-10.484	6.913	-0.099
-3.288	3.385	3.558	-0.571	-5.419	7.090	--	3.622	3.499	3.445
-0.293	-0.114	0.292	--	-2.994	0.417	--	-2.948	0.297	-0.213
-0.318	0.717	0.785	2.18	0.468	0.097	--	0.789	0.692	1.592
-0.143	0.190	0.460	--	-0.221	0.268	--	-0.519	1.233	0.090
-0.197	1.363	1.386	2.61	0.546	0.353	--	1.005	0.922	2.771
-0.119	-0.041	0.294	--	-0.499	-0.420	--	-0.285	-0.444	-0.054
-0.340	0.030	0.024	0.372	-0.256	0.391	--	-0.236	-0.170	-0.113
-0.437	-0.113	0.205	--	-0.660	0.190	--	-0.137	0.311	0.099
-0.463	0.033	0.284	0.368	-0.273	0.379	--	-0.185	-0.107	-0.141
-0.428	-0.236	0.302	--	-0.823	0.082	--	-0.273	-0.134	-0.112
-0.417	0.182	0.151	0.2	-0.320	0.456	--	-0.256	-0.266	-0.329
-0.375	-0.171	1.025	--	-0.670	0.011	--	0.145	0.162	0.688
-0.327	0.387	0.607	0.448	-0.083	0.934	--	-0.024	-0.005	-0.190
-0.425	0.027	0.163	--	-0.500	0.064	--	-0.600	0.008	-0.077
0.155	-0.042	-0.003	0.185	-0.252	0.088	--	-0.186	-0.077	-0.053
-0.407	0.033	0.169	--	-0.289	0.353	--	-0.520	0.003	-0.034
-0.189	0.127	0.003	0.25	-0.165	0.155	--	-0.072	-0.032	-0.027
-0.395	-0.129	0.613	--	-0.673	0.294	--	-0.255	0.100	0.175
-0.352	0.049	0.048	0.282	-0.163	0.260	--	-0.139	-0.113	-0.062
-0.624	-0.297	0.687	--	-0.909	0.199	--	-0.482	-0.061	0.221
-0.451	-0.085	-0.208	0.171	-0.658	0.207	--	-0.363	-0.503	-0.160
-3.013	-3.001	1.538	--	-2.920	-2.330	--	-1.592	-1.702	-1.001
-2.794	-2.698	-0.415	2.135	1.279	-2.440	--	-1.978	-2.389	-2.127
-2.505	-2.332	1.237	--	-2.733	-2.306	--	-1.766	-2.264	-1.286
-2.990	-3.066	-0.710	1.807	1.065	-2.778	--	-2.282	-2.901	-2.676
-4.946	-5.016	2.113	--	-5.120	-4.296	--	-0.988	-4.505	-0.365
-5.200	-3.805	0.180	3.182	2.099	-5.030	--	-1.958	-4.598	-1.358
-8.050	-7.601	1.622	--	-8.025	-7.457	--	-2.652	-6.961	-3.542
-6.859	-6.680	-0.856	1.776	1.017	-6.516	--	-3.282	-7.492	-4.762
-0.838	-0.523	1.421	--	-0.618	-0.402	--	-0.327	-1.418	0.152
-2.017	-1.936	-0.149	1.284	0.956	-1.904	--	-1.608	-2.104	-1.864
-3.467	-2.058	1.224	--	-4.758	-5.545	--	0.001	-2.344	1.275
-2.516	-1.544	-0.289	1.466	1.717	-4.329	--	-0.207	-3.291	1.281
-2.823	-1.741	1.154	--	-3.595	-3.014	--	-0.126	-1.977	1.109
-2.437	-1.604	-0.332	1.499	1.706	-2.745	--	-0.331	-2.493	1.120
-1.168	-0.329	1.302	--	-0.893	-0.902	--	0.013	-1.216	1.541
-0.754	0.378	0.895	1.284	0.654	-0.798	--	0.201	-1.304	1.434

-2.554	-1.536	2.161	--	-2.494	-4.221	--	-0.384	-4.445	1.219
-2.192	-1.460	0.741	1.046	0.307	-4.313	--	-0.805	-3.720	0.171
-1.845	-1.085	1.523	--	-1.211	-0.584	--	0.722	-2.656	1.943
-1.767	0.400	1.059	1.953	1.456	-1.318	--	0.569	-2.971	2.153
-2.945	-2.091	1.455	--	-2.604	-1.727	--	0.159	-4.048	1.956
-2.392	0.008	0.632	1.31	0.543	-2.319	--	0.030	-3.846	1.217
-3.814	-0.619	2.159	--	-3.007	-4.253	--	1.399	-4.855	2.714
-2.735	1.285	1.584	3.101	2.417	-6.137	--	1.700	-5.890	3.562
-5.494	-1.696	1.674	--	-5.656	-4.515	--	0.153	-4.819	1.674
-2.207	-0.128	1.266	1.665	1.241	-5.962	--	-0.095	-5.851	2.085
-5.413	-2.539	1.416	--	-4.510	-5.903	--	-0.135	-5.788	1.489
-3.245	-1.195	0.702	0.915	0.373	-5.962	--	-0.084	-6.596	0.907
-5.483	-3.448	0.964	--	-5.638	-9.194	--	-1.451	-7.328	0.571
-2.113	-1.835	-2.023	-1.816	-0.673	-9.121	--	-3.515	-8.049	-0.261
-5.931	-6.425	1.248	--	-6.462	-5.223	--	-1.143	-6.442	0.604
-2.673	-2.305	0.446	-0.509	0.135	-3.847	--	-0.735	-5.739	-0.706
-8.407	-5.807	1.218	--	-8.367	-7.991	--	-2.589	-8.088	0.415
-3.282	-4.854	0.359	-0.302	-0.686	-6.525	--	-2.865	-8.372	0.488
-0.152	-0.145	0.196	--	0.322	1.778	--	-0.456	-1.044	0.697
-0.264	1.315	0.595	1.493	1.908	1.768	--	-0.052	-1.154	1.230
5.550	2.046	-2.123	--	-1.120	-0.370	--	-1.994	2.314	1.459
1.586	-0.758	-2.119	1.313	-0.717	1.561	--	-2.380	-2.330	2.746
6.860	1.641	3.628	--	1.919	2.302	--	2.226	3.650	2.953
3.150	0.895	1.315	3.415	3.096	1.633	--	0.440	1.352	4.294
1.656	1.612	0.758	--	1.067	0.386	--	-0.469	1.715	1.766
0.884	0.505	0.457	1.185	1.350	-0.230	--	-0.334	0.132	0.873
0.948	0.496	-4.158	--	-4.957	-3.793	--	-3.328	1.101	1.509
1.359	0.289	-1.899	-0.222	-2.850	-3.115	--	-0.782	-1.779	0.718
0.239	0.754	-0.388	--	-1.349	-1.233	--	-0.868	1.390	0.335
1.495	0.843	-0.071	0.225	-0.743	-2.926	--	-0.727	-0.273	-0.057
-5.689	0.161	-4.779	--	-5.497	-5.248	--	-0.559	0.799	1.313
1.528	-0.003	-1.073	-0.796	-3.573	-5.004	--	-0.182	-0.569	1.993
0.125	1.010	-0.887	--	-0.090	-0.825	--	-1.482	1.354	-0.155
1.399	0.994	0.722	-1.826	-1.324	-3.250	--	-0.155	1.003	-0.278
2.304	1.888	-4.013	--	-2.299	-3.069	--	-3.998	0.623	0.607
1.649	1.544	-0.425	0.485	-3.473	-2.782	--	-3.467	-2.658	2.203
0.173	0.526	-0.191	--	-0.146	-0.985	--	-0.408	0.511	0.624
0.606	0.450	0.236	-0.062	0.045	-1.474	--	0.377	0.153	1.037
1.118	1.708	-0.230	--	-0.597	-2.045	--	-0.633	1.872	2.231
1.066	1.438	0.543	0.151	-1.609	-2.828	--	-0.313	-0.213	0.651
1.059	1.396	-0.895	--	-2.029	-1.876	--	-1.064	2.082	1.409
1.447	1.375	0.080	0.244	-1.726	-2.787	--	-0.664	0.020	-0.365
1.406	0.751	-0.760	--	-0.345	-0.943	--	-1.032	1.286	1.558
0.400	0.518	0.214	0.33	-1.622	-2.031	--	-0.835	-0.147	-0.079
2.143	1.514	-0.435	--	0.613	-0.493	--	-1.123	1.472	0.704
1.856	1.334	0.314	0.498	-1.168	-1.865	--	-0.892	-0.184	-0.276
1.608	1.843	-0.350	--	0.130	-0.293	--	-0.362	1.919	1.293

1.544	1.550	0.209	0.529	-0.740	-0.857	--	-0.334	-0.049	0.058
1.755	1.520	-0.821	--	-0.097	-0.815	--	-1.501	1.131	1.494
2.023	0.955	0.204	0.585	-1.221	-1.704	--	-1.127	-0.235	2.114
1.716	1.348	-0.655	--	0.864	-0.561	--	-0.988	0.918	0.100
1.614	1.518	0.440	0.324	-1.186	-2.027	--	-0.718	0.005	0.455
0.344	0.232	2.533	--	0.272	0.045	--	1.479	0.816	3.571
0.100	-0.489	3.473	2.636	2.889	3.384	--	2.821	3.865	1.259
-0.487	-0.577	1.117	--	0.643	0.536	--	0.700	1.228	1.218
0.144	0.194	0.869	0.622	1.324	1.662	--	0.429	0.744	1.022
-0.391	-0.073	0.751	--	-0.673	-0.039	--	-0.277	0.404	0.662
0.548	-0.881	0.027	0.141	-0.136	-0.478	--	-0.513	0.385	-0.075
2.064	2.479	3.729	--	-0.692	0.294	--	1.144	2.641	3.169
2.648	2.331	4.126	1.267	2.059	-0.041	--	2.666	3.207	1.573
-0.142	-0.285	0.284	--	-0.607	-0.269	--	-0.814	0.044	0.295
-0.041	-1.362	0.224	0.441	-0.099	0.085	--	0.073	-0.004	0.114
-0.391	-0.489	0.365	--	-0.518	0.073	--	-0.311	0.116	0.107
-0.101	-1.344	0.000	-0.024	0.000	0.020	--	-0.042	0.060	0.034
0.000	0.000	0.000	--	0.000	0.556	--	0.000	0.272	0.000
0.028	-0.103	-0.404	-0.31	0.066	0.038	--	0.126	0.113	-0.185
0.161	0.245	0.393	--	-0.029	0.261	--	-0.175	-0.175	0.309
-0.134	-0.647	-4.475	-2.087	0.633	0.368	--	0.112	-2.028	-1.570
-0.385	0.021	0.238	--	-0.495	-0.029	--	-0.389	-0.261	0.217
-0.238	-1.552	-0.015	-0.656	0.388	-0.182	--	-0.147	0.314	-0.199
0.547	1.025	-0.787	--	0.188	0.167	--	0.159	1.586	0.771
0.928	0.556	3.111	1.203	1.915	0.977	--	0.129	1.558	0.335
2.792	0.538	0.832	--	1.999	3.101	--	0.472	0.358	0.307
-0.353	-0.756	-0.123	-0.224	0.291	0.849	--	0.486	0.181	0.019
2.156	1.161	1.796	--	1.798	2.328	--	2.363	1.191	1.318
-0.400	-0.062	0.557	0.739	1.093	0.666	--	1.121	0.785	1.414
2.211	2.316	0.697	--	2.724	2.197	--	1.474	1.345	1.355
-0.705	-0.696	-0.010	-0.654	0.435	1.072	--	0.987	0.572	0.654
1.467	1.523	1.384	--	1.397	2.028	--	1.763	1.015	1.812
-0.798	-0.874	-0.498	0.279	0.916	1.087	--	1.712	0.418	2.267
1.934	2.160	1.290	--	1.375	1.920	--	2.095	3.406	2.155
-0.175	0.355	-0.367	1.968	3.235	1.636	--	3.500	1.402	2.644
1.035	1.323	1.399	--	1.303	0.776	--	2.141	1.449	2.384
-0.240	0.608	0.305	0.291	0.442	0.944	--	1.688	0.820	1.748
-0.095	-0.292	-0.026	--	0.045	-0.225	--	0.257	-0.286	0.039
-0.609	-1.390	-0.385	-0.58	-0.100	0.329	--	0.077	0.180	-0.145
-0.130	-0.277	0.130	--	-0.090	0.196	--	0.217	-0.148	0.025
-0.447	-0.768	-0.100	-0.056	0.308	0.462	--	0.390	0.179	0.160
0.585	1.258	0.190	--	1.124	1.243	--	1.234	1.053	1.730
-0.498	0.418	0.529	0.428	0.131	-0.516	--	1.616	0.216	1.263

**74**	**75**	**76**	**77**	**78**	**79**	**80**	**81**	**82**	**83**
82.60	113.20	--	87.60	99.00	123.00	174.60	77.80	125.00	173.20
92.40	101.80	77.60	102.80	122.60	122.00	121.20	72.20	109.00	155.60
19.10	14.50	--	15.70	16.70	17.70	16.10	12.30	12.80	12.00
15.60	14.40	14.80	13.90	14.50	15.30	16.50	14.50	13.70	13.40
17.62	14.98	15.24	--	17.58	18.24	--	15.58	16.48	18.68
24.24	18.64	--	21.98	25.39	18.62	24.28	22.13	17.58	20.57
24.05	22.00	24.47	26.50	29.59	21.35	24.38	24.68	24.74	19.12
32.53	22.73	--	25.60	40.00	24.40	30.65	23.55	22.23	23.62
29.69	23.67	30.42	33.16	41.02	25.20	37.88	30.82	26.65	23.43
76.11	88.45	--	80.83	65.35	84.51	79.15	91.33	96.09	88.00
82.83	82.22	80.83	69.77	67.51	87.50	64.51	72.42	84.43	81.31
58.00	14.00	--	21.00	42.00	42.00	48.00	6.00	16.00	28.00
41.00	9.00	2.00	13.00	27.00	27.00	41.00	6.00	13.00	27.00
168.00	130.00	124.00	--	152.00	158.00	--	124.00	134.00	158.00
32.17	51.17	--	13.50	16.58	4.10	20.50	--	41.00	6.78
44.33	30.67	--	6.25	16.33	21.75	17.92	2.27	9.68	5.05
23.44	34.42	13.14	--	20.20	3.24	--	8.73	6.18	3.53
2	--	1	2	2	--	2	1	1	1
2	--	1	2	2	1	2	1	1	1
2	2	0	--	2	0	--	0	0	0
0.324	-0.673	--	0.001	0.078	-0.055	0.226	-0.330	-0.226	-0.330
0.146	-0.199	-0.041	0.235	0.392	-0.024	0.752	-0.161	0.222	0.443
0.298	-0.466	--	-0.032	0.018	-0.176	0.130	-0.263	0.152	-0.166
0.096	0.010	0.05	0.035	0.380	0.337	0.295	0.026	0.224	0.364
0.146	-1.745	--	-2.977	0.196	-0.502	0.093	-1.770	-0.364	-0.093
0.154	-0.166	-0.033	0.173	0.351	0.567	0.226	0.699	1.175	0.307
0.114	-0.020	--	-0.055	-0.183	0.198	-0.237	-0.252	-0.079	-0.229
-2.216	0.618	0.525	3.399	2.702	-0.185	-2.493	2.924	5.541	-0.842
0.193	-0.030	--	0.195	0.147	-0.222	0.137	0.185	0.125	-0.246
0.681	0.242	-0.192	0.035	0.586	0.495	0.319	0.232	0.294	0.355
0.318	0.072	--	-0.174	0.013	-0.411	0.040	0.134	0.138	-0.406
0.535	-0.057	-0.221	-0.138	0.430	0.584	0.427	0.246	0.298	0.455
-0.127	0.230	--	-0.107	-0.292	-0.610	-0.246	0.052	-0.171	-0.359
0.512	0.197	-0.149	0.002	0.336	0.368	0.305	0.131	0.220	0.235
-0.177	0.294	--	-0.205	-0.434	-0.577	-0.373	0.100	-0.071	-0.412
0.478	0.224	-0.189	0.001	0.266	0.282	0.301	0.152	0.196	0.265
-0.169	0.209	--	-0.163	-0.325	-0.632	-0.375	0.068	-0.100	-0.403
0.465	0.207	-0.15	0.034	0.271	0.259	0.283	0.177	0.187	0.262
0.100	0.181	--	0.057	-0.234	-0.569	-0.178	0.146	0.146	-0.312
0.541	0.242	-0.105	0.060	0.393	0.520	0.365	0.187	0.462	0.365
-0.089	0.170	--	-0.181	-0.397	-0.655	-0.277	-0.032	-0.190	-0.514
0.482	0.007	-0.252	-0.193	0.162	0.373	0.382	0.139	0.179	0.298
-0.729	-0.572	--	-0.557	-0.490	-0.895	-0.461	-0.246	-0.615	-1.059
-0.034	-0.112	-0.462	-0.332	0.193	0.030	0.029	-0.081	-0.342	0.265
0.376	0.444	--	0.103	-0.213	-0.438	-0.108	0.373	0.307	-0.316

0.626	0.128	-0.076	0.233	0.604	0.735	0.558	0.244	0.523	0.474
2.387	0.370	--	0.266	1.743	-0.526	-0.284	0.063	1.318	-1.746
0.370	-2.205	-2.478	-0.805	2.452	2.646	0.258	1.108	2.757	3.677
-1.719	-1.445	--	-3.330	-2.590	-2.405	-3.712	-3.107	-2.581	-2.347
-1.302	-1.793	1.334	-1.664	-1.075	0.544	-1.117	2.312	2.041	1.242
0.376	-1.015	--	0.137	-0.908	-0.378	-0.624	-0.122	0.199	-0.898
0.883	0.689	0.369	0.649	0.091	0.118	1.992	-0.126	-0.499	-0.552
0.030	-5.553	--	-0.028	-1.353	-0.793	-0.543	-4.613	-2.044	-1.205
1.161	1.109	0.905	0.585	-0.107	-0.140	2.714	-0.076	-0.263	-1.075
0.378	-3.767	--	-0.045	-3.468	0.159	-3.600	-0.240	3.487	-3.742
3.577	10.822	3.586	2.760	3.566	-3.714	5.243	-0.044	3.414	-7.178
-0.171	0.317	--	0.014	-3.762	-2.973	-3.231	0.146	0.178	-2.956
1.938	1.809	1.283	0.803	0.072	0.134	3.843	0.062	-0.106	-1.161
0.038	0.213	--	0.022	-0.276	-0.330	-0.280	0.462	0.288	-0.356
1.809	2.709	2.262	1.050	0.361	-0.027	3.688	0.229	0.346	-1.839
-0.228	0.297	--	0.173	0.118	-0.757	0.435	0.360	-0.139	-0.029
0.357	0.014	0.246	0.155	0.473	0.153	0.412	-0.132	0.176	-0.104
0.001	0.219	--	0.383	0.134	-0.411	0.274	0.342	-0.075	-0.161
0.268	0.159	0.128	0.100	0.300	0.234	0.329	-0.076	0.137	-0.043
-0.332	0.171	--	-0.133	-0.033	-0.339	0.076	0.294	-0.319	-0.191
0.404	-0.022	-0.004	0.105	0.450	0.114	0.354	-0.237	0.262	-0.039
-0.104	0.528	--	0.297	0.228	-0.227	0.221	0.684	0.141	-0.084
0.785	0.345	0.011	0.441	0.966	0.755	0.673	-0.126	0.330	0.144
0.034	0.043	--	-0.137	-0.102	-0.243	-0.159	-0.061	-0.192	-0.515
0.090	-0.018	-0.015	-0.050	0.119	0.019	0.438	-0.132	-0.055	-0.021
-0.004	-0.005	--	-0.101	-0.076	-0.350	-0.097	-0.167	-0.143	-0.260
0.211	0.088	0.137	-0.004	0.121	0.156	0.366	-0.005	0.155	0.113
0.134	0.054	--	0.004	0.000	-0.426	0.087	0.155	-0.176	-0.284
0.326	0.101	0.128	0.004	0.143	0.097	0.472	-0.030	0.076	-0.060
-0.130	0.081	--	-0.160	-0.153	-0.598	-0.156	0.292	-0.233	-0.473
0.123	-0.048	-0.091	-0.109	-0.020	-0.106	0.484	-0.018	-0.033	-0.079
-1.880	-0.942	--	-1.627	-2.692	-2.624	-2.807	-0.223	-2.422	-2.751
0.556	-1.202	-2.336	0.452	-2.404	-2.468	-2.596	-2.397	-2.630	-2.027
-1.914	-0.904	--	-1.703	-2.470	-2.244	-2.159	-0.598	-1.963	-2.525
-0.113	-1.660	-2.726	0.423	-2.594	-2.503	-3.283	-2.503	-3.259	-2.457
-4.662	-0.827	--	-1.709	-4.872	-4.936	-5.050	0.268	-4.179	-5.096
1.238	-0.738	-2.615	1.734	-4.427	-4.895	-5.229	-3.809	-5.139	-5.078
-5.768	-1.443	--	-3.790	-7.812	-7.291	-6.586	-0.813	-7.308	-7.525
-0.137	-2.218	-3.86	-0.297	-6.961	-7.090	-7.655	-4.916	-7.481	-6.979
0.324	-0.303	--	-0.272	-1.830	-0.496	-1.387	0.234	-1.033	-0.476
0.668	-0.468	-1.613	0.090	-1.907	-1.276	-1.589	-1.611	-2.834	-1.990
-2.994	0.208	--	0.213	-4.900	-3.536	-2.876	0.278	-3.384	-4.367
0.193	-0.102	0.343	0.971	-3.107	-1.575	-2.107	-0.584	-3.116	-4.394
-2.418	-0.042	--	0.229	-3.870	-2.950	-2.558	0.336	-2.714	-3.350
0.217	-0.106	0.22	0.900	-2.153	-1.393	-1.551	-0.635	-2.737	-4.768
0.138	0.911	--	1.503	-1.408	-0.825	-0.897	0.954	-1.435	-1.652
0.785	0.650	0.863	1.152	-0.869	-0.194	0.168	0.151	-1.043	-1.003

-0.369	1.088	--	1.158	-4.605	-1.737	-2.952	0.790	-4.351	-4.420
-0.598	0.271	0.19	1.372	-3.651	-1.251	-2.121	0.095	-3.920	-3.472
-1.289	0.841	--	0.688	-2.532	-2.396	-1.594	1.447	-2.364	-3.030
1.258	1.088	1.146	0.759	-2.809	-1.163	-0.217	0.067	-2.439	-3.852
-1.106	0.656	--	0.672	-3.084	-4.093	-2.292	0.926	-5.276	-5.294
0.345	0.540	0.575	0.696	-2.736	-2.132	-0.857	-0.037	-2.969	-5.236
-1.356	0.809	--	1.753	-5.787	-2.703	-1.936	1.739	-5.307	-5.869
1.848	1.699	2.172	1.746	-6.137	-0.677	0.234	0.704	-6.135	-5.512
-2.022	0.231	--	0.846	-5.373	-4.747	-3.831	1.008	-5.037	-5.573
0.928	0.637	0.809	1.134	-5.962	-1.532	-0.894	0.343	-6.078	-5.686
-3.158	1.263	--	0.555	-6.079	-3.465	-3.690	1.591	-5.490	-5.613
-0.515	0.712	0.425	0.765	-5.419	-1.845	-3.295	-0.244	-5.341	-6.720
-3.040	-2.783	--	-0.705	-8.175	-4.038	-4.295	-0.296	-6.546	-7.712
-0.870	-1.300	-2.809	0.333	-6.665	-2.179	-5.984	-0.430	-10.305	-7.432
-5.811	-2.089	--	0.095	-6.194	-6.444	-6.062	-0.709	-5.870	-6.358
-0.730	-3.330	-1.371	2.261	-3.866	-2.253	-3.817	0.104	-5.649	-6.392
-6.164	-1.763	--	-0.815	-7.225	-7.058	-6.714	-1.883	-7.610	-8.201
-1.972	-2.179	-1.256	1.356	-6.441	-5.184	-4.259	-2.439	-7.052	-7.360
-0.119	0.469	--	0.002	-0.723	-1.157	0.069	0.104	-0.511	-2.336
1.437	1.551	0.759	-0.036	-0.309	-0.517	1.715	-0.392	-0.814	-1.970
5.721	-2.969	--	-1.765	-1.436	-1.523	-1.174	-2.923	-0.986	-1.891
0.216	-2.092	-2.68	4.850	-1.894	-1.357	-0.420	2.611	-2.267	0.102
10.301	1.168	--	3.079	1.668	2.787	2.398	0.433	1.494	-0.580
0.865	1.760	-2.121	7.358	2.374	2.501	2.312	2.289	-1.168	2.012
1.978	0.078	--	2.930	1.713	1.459	2.363	0.259	0.072	0.253
-0.065	0.524	1.263	1.384	1.156	0.543	1.327	0.672	0.107	-0.364
0.019	-4.392	--	0.803	0.879	0.675	0.865	-4.822	-4.270	-2.393
-0.102	1.129	-2.301	1.547	0.592	0.795	0.250	1.840	-0.581	0.836
-0.114	-1.427	--	0.117	0.852	0.695	0.977	-3.126	-1.729	-0.893
0.628	0.970	-0.404	0.568	0.814	0.733	0.709	0.866	0.126	1.099
-5.075	-2.350	--	0.208	-0.650	-0.961	-1.868	-5.322	-2.194	-2.875
-0.018	0.784	-1.148	2.109	0.042	0.547	-0.782	1.644	-0.564	1.293
0.328	-0.749	--	-0.300	0.516	0.838	1.012	-5.306	0.236	0.041
0.201	1.437	1.079	-0.492	0.114	-0.177	0.999	-0.360	2.079	0.425
1.059	-3.864	--	0.147	0.762	-2.489	0.248	-3.846	-3.631	-3.300
0.244	-3.446	-3.52	2.590	0.506	-1.824	1.412	2.244	-0.522	0.291
0.566	-1.282	--	0.689	-0.053	-0.358	-0.151	-1.486	-0.603	-1.225
0.179	-0.128	0.49	0.915	0.231	-0.143	0.602	0.670	0.058	-0.501
1.366	-3.243	--	2.397	1.482	1.024	1.860	-3.458	-1.940	-1.437
1.292	0.430	-0.089	1.930	1.254	0.443	1.131	1.248	-0.681	-0.301
1.233	-3.118	--	2.139	1.531	1.214	2.734	-3.374	-2.526	-1.487
0.957	1.215	-0.717	2.156	1.268	1.014	1.170	1.137	-1.248	0.547
1.635	-3.298	--	1.237	1.488	0.942	1.550	-3.507	-2.273	-0.692
0.294	0.267	-1.113	0.459	0.767	0.417	0.202	0.424	-1.132	0.240
1.547	-2.358	--	1.231	1.253	0.634	1.507	-2.921	-2.008	-0.836
0.714	-0.296	-0.706	1.663	0.875	0.041	1.991	1.173	-0.191	0.035
1.459	-1.365	--	1.637	1.224	0.781	1.756	-1.683	-1.289	-0.154

1.018	0.410	-0.488	1.736	0.759	0.233	1.861	1.173	-0.205	0.179
1.840	-3.156	--	1.253	1.330	0.588	2.291	-3.791	-2.112	-0.955
0.447	-0.120	-0.78	2.942	0.901	-0.461	1.394	1.042	-0.590	-0.381
1.190	-5.346	--	0.407	0.893	0.291	0.671	-3.154	-3.023	-0.619
0.552	-0.377	-0.012	0.997	0.843	0.418	1.664	0.758	0.428	0.501
1.968	2.306	--	2.280	1.519	0.711	0.726	2.240	3.045	1.145
0.049	2.152	1.332	1.621	0.269	0.498	1.955	2.094	0.889	-0.365
0.378	1.433	--	1.674	1.019	0.649	0.395	1.226	1.144	1.086
0.314	0.984	0.524	0.429	1.287	0.938	0.367	0.269	0.343	0.278
0.995	-0.090	--	0.557	-0.171	0.023	0.088	-0.287	0.579	-0.254
-0.633	-0.388	-0.461	0.474	-0.276	0.154	-0.781	0.043	-0.093	0.147
3.782	1.309	--	3.041	-0.053	-1.229	2.184	0.175	-0.080	1.348
1.679	0.693	0.009	2.299	1.113	1.372	-0.074	1.504	2.931	1.467
0.331	-0.098	--	0.326	-0.100	-0.284	-0.082	-0.115	-0.104	-0.690
0.264	0.378	-0.055	0.532	-0.134	-0.059	0.307	-0.040	-1.538	-0.290
0.167	-0.171	--	-1.024	-0.213	-0.043	-0.051	-1.517	0.314	-0.216
0.024	0.042	0.039	0.132	-0.005	-0.033	0.056	0.065	-1.392	0.000
0.000	0.000	--	0.000	0.447	0.000	-0.042	0.000	0.000	0.000
0.015	0.133	-0.167	-0.156	0.335	0.301	-0.524	-0.391	-0.211	0.571
0.484	-0.201	--	-0.014	0.010	0.073	0.395	-0.195	0.261	-0.268
-2.398	-1.360	-1.521	-0.202	0.081	0.249	-5.257	0.226	0.015	1.720
0.039	-0.029	--	0.114	-0.393	-0.346	-0.212	-0.176	0.074	-0.491
0.335	-0.082	0.058	-1.385	0.314	0.237	-0.021	0.069	-0.276	-0.079
0.272	0.184	--	0.255	1.253	0.717	1.333	-0.321	-0.181	-0.302
0.102	0.157	0.186	0.310	2.137	1.805	1.150	0.306	0.052	0.755
2.097	0.842	--	-0.033	0.895	0.983	1.487	0.671	0.845	0.007
-0.526	0.996	0.853	-0.295	-0.842	-0.474	0.013	-0.413	0.082	-1.335
3.130	1.224	--	0.732	0.755	0.854	1.391	0.906	2.318	0.306
-0.154	1.781	1.383	0.386	-0.401	-0.136	0.906	0.627	0.728	-1.518
2.571	1.782	--	0.922	1.652	1.437	1.429	1.943	1.775	1.149
-0.483	1.054	1.832	-1.436	-0.905	-0.799	-0.568	-0.721	0.614	-1.183
2.273	1.417	--	1.156	0.650	1.588	0.781	2.105	1.883	1.085
-0.788	1.412	2.109	0.893	-0.777	-0.394	-0.785	0.204	0.403	-0.904
1.763	2.384	--	2.117	2.665	1.714	1.681	1.443	2.136	1.401
0.094	3.617	3.023	2.123	0.543	1.041	0.627	1.965	2.959	-1.184
1.306	2.373	--	2.546	0.949	0.857	1.152	2.359	1.802	1.887
-0.056	2.245	1.53	1.112	-0.101	-0.348	-0.007	1.095	0.604	-0.341
-0.026	0.397	--	0.312	-0.055	0.134	0.145	0.356	0.327	0.093
-1.091	0.496	0.379	-0.227	-1.649	-0.433	-0.498	-0.098	0.092	-1.612
0.203	0.247	--	0.050	-0.154	-0.006	0.042	0.238	0.241	-0.094
-0.257	0.270	0.406	-0.317	-0.909	-0.082	-0.305	-0.133	0.046	-0.698
1.407	0.676	--	0.979	0.079	0.288	-0.867	1.070	-0.320	-0.641
0.511	1.420	1.113	0.277	-0.105	-0.109	0.124	0.788	0.659	-0.882

85	86	88	90	91	92	93	94	96	97
164.00	149.20	92.00	111.00	125.00	--	110.40	--	107.00	--
155.20	109.60	92.60	118.60	151.40	106.80	145.40	109.40	--	97.80
16.50	13.10	13.60	15.70	18.50	--	12.70	--	15.70	--
16.90	13.90	13.80	14.50	14.50	--	13.90	15.50	--	14.40
19.40	--	16.04	--	16.16	14.92	15.26	15.44	16.76	13.42
22.48	20.01	20.82	17.88	21.07	--	16.52	--	23.42	--
22.36	22.31	23.38	23.74	19.47	22.85	23.85	28.08	--	23.56
28.66	25.49	27.19	21.17	25.94	--	20.95	--	30.61	--
26.15	25.66	26.98	27.94	24.29	30.60	27.68	31.43	--	29.11
81.92	84.92	87.49	90.47	80.84	--	87.28	--	82.32	--
81.55	85.87	85.04	79.73	85.72	78.74	85.14	77.41	--	81.16
55.00	21.00	16.00	28.00	55.00	--	28.00	--	28.00	--
44.00	13.00	13.00	23.00	37.00	20.00	16.00	23.00	--	9.00
175.00	--	130.00	--	172.00	134.00	144.00	164.00	148.00	134.00
29.08	5.78	--	39.83	16.12	--	1.28	--	45.58	--
41.83	15.33	4.43	5.30	25.83	1.83	15.93	19.50	--	2.32
29.71	--	12.55	--	3.73	2.55	6.18	21.18	17.75	2.45
--	1	1	1	1	1	1	2	2	1
--	1	1	1	1	1	1	2	2	1
2	--	0	--	0	0	0	2	2	0
-0.469	0.097	-0.396	-0.393	-0.454	--	-0.115	--	-0.239	--
0.178	0.043	-0.014	0.523	-0.345	--	0.079	0.341	--	0.14
-0.483	0.373	-0.242	-0.463	-0.398	--	-0.171	--	0.257	--
0.113	0.098	0.009	0.342	-0.125	--	0.237	0.334	--	0.039
-0.770	0.040	-1.194	-2.902	-2.708	--	-0.950	--	-0.119	--
0.813	0.245	0.219	1.350	-0.212	--	-0.030	0.675	--	0.096
-0.156	-1.514	0.297	0.382	0.027	--	-0.015	--	-0.062	--
4.019	-1.714	3.203	5.404	1.148	--	-5.511	2.312	--	-2.699
-0.284	0.693	-0.035	-0.647	-0.777	--	0.059	--	0.525	--
0.449	0.159	0.019	0.478	0.543	--	0.582	0.206	--	0.509
-0.578	0.648	-0.480	-0.402	-0.744	--	-0.004	--	0.65	--
0.200	0.234	-0.137	0.322	0.336	--	0.560	0.266	--	0.59
-0.291	0.486	0.002	-0.437	-0.648	--	0.002	--	0.465	--
0.305	0.216	-0.031	0.332	0.297	--	0.480	0.193	--	0.443
-0.220	0.494	0.028	-0.504	-0.532	--	0.146	--	0.501	--
0.265	0.186	-0.074	0.357	0.267	--	0.465	0.136	--	0.436
-0.533	0.412	0.008	-0.623	-0.609	--	-0.158	--	0.397	--
0.257	0.162	-0.042	0.302	0.217	--	0.440	0.171	--	0.429
-0.520	0.582	0.118	-0.483	-0.466	--	-0.052	--	0.503	--
0.399	0.296	0.074	0.417	0.407	--	0.559	0.261	--	0.503
-0.597	0.419	0.032	-0.657	-0.603	--	0.023	--	0.505	--
0.180	0.195	-0.075	0.312	0.261	--	0.444	0.134	--	0.549
-0.908	0.506	-0.599	-1.317	-1.165	--	-0.542	--	-0.411	--
-0.468	-0.272	-0.493	-0.305	-0.248	--	-0.160	-0.165	--	0.211
-0.506	0.767	0.178	-0.303	-0.743	--	0.103	--	0.981	--

0.386	0.401	-0.006	0.298	0.479	--	0.674	0.448	--	0.508
-0.214	0.691	-2.082	-0.476	-3.193	--	-0.038	--	1.48	--
2.514	2.951	-1.360	2.011	4.040	--	1.828	-0.425	--	1.598
-4.037	0.899	-2.426	-0.338	-2.913	--	1.098	--	-2.825	--
-2.404	1.139	1.095	0.532	-0.867	--	0.964	0.407	--	-1.008
-0.008	0.320	1.188	-3.416	-1.099	--	0.464	--	-0.583	--
0.868	1.177	0.151	0.727	1.277	--	0.394	0.433	--	0.45
-0.219	0.144	2.666	-3.240	-5.382	--	3.563	--	-0.697	--
1.283	1.650	0.269	0.992	1.750	--	0.622	0.643	--	0.6
5.153	-2.489	0.143	-3.569	-0.778	--	-2.631	--	-10.402	--
7.099	-0.010	3.459	5.311	6.936	--	-3.599	-0.457	--	-7.351
0.132	-1.517	0.185	-2.760	-0.229	--	0.066	--	0.093	--
1.594	2.298	0.233	0.938	2.767	--	1.059	0.871	--	1.451
0.054	1.426	0.160	-0.027	-0.459	--	0.233	--	0.131	--
1.909	2.713	1.412	1.181	3.397	--	1.127	1.03	--	1.388
0.211	0.714	0.410	-0.502	-0.874	--	0.108	--	0.206	--
0.156	0.114	-0.108	0.262	-0.065	--	0.252	0.215	--	0.276
0.014	0.605	0.205	-0.708	-0.764	--	0.182	--	0.583	--
0.196	0.183	-0.014	0.204	0.126	--	0.334	0.209	--	0.22
-0.025	0.495	0.179	-0.443	-0.753	--	-0.052	--	0.29	--
0.172	0.274	-0.113	0.290	0.196	--	0.435	0.223	--	0.326
0.103	0.820	0.428	-0.410	-0.902	--	0.088	--	1.117	--
0.621	0.327	-0.095	0.575	0.444	--	0.864	0.364	--	0.524
-0.028	0.308	0.167	-0.565	-0.625	--	0.320	--	0.127	--
0.214	0.097	-0.101	0.063	0.106	--	0.087	0.148	--	0.568
0.020	0.364	0.026	-0.184	-0.345	--	0.355	--	0.366	--
0.148	0.136	0.105	0.198	-0.040	--	0.167	0.205	--	0.09
0.148	0.499	-0.058	-0.542	-0.821	--	0.157	--	0.426	--
0.126	0.190	0.022	0.190	0.175	--	0.335	0.182	--	0.275
-0.086	0.363	-0.029	-0.623	-0.805	--	0.040	--	0.396	--
-0.019	0.015	-0.144	0.085	-0.242	--	0.433	0.083	--	0.232
-3.078	-0.400	0.441	-3.487	-2.997	--	-2.310	--	-2.114	--
-2.523	-1.291	-1.232	-2.512	-1.630	--	1.293	-0.609	--	-1.67
-2.954	-0.711	0.438	-2.650	-2.781	--	-1.763	--	-1.654	--
-2.911	-1.743	-1.753	-2.578	-2.312	--	1.000	-0.796	--	-2.155
-4.815	-0.243	1.112	-5.132	-4.891	--	-4.325	--	-4.634	--
-3.510	-0.954	-1.001	-3.889	-1.088	--	1.973	-1.026	--	-1.488
-7.194	-1.254	0.042	-8.051	-8.167	--	-7.098	--	-6.327	--
-5.372	-2.660	-2.060	-5.500	-3.259	--	1.104	-2.406	--	-4.442
-1.317	-0.126	0.273	-1.479	-1.083	--	-0.234	--	-0.525	--
-1.422	-1.124	-0.669	-1.688	-1.332	--	1.025	-0.232	--	-1.293
-2.463	1.052	1.290	-2.275	-3.666	--	0.880	--	-0.884	--
-1.361	-0.068	-0.620	-1.305	-0.297	--	1.505	-0.464	--	-0.243
-2.198	0.933	1.302	-2.318	-3.300	--	0.748	--	-1.13	--
-1.396	-0.075	-0.757	-1.530	-0.280	--	1.556	-0.49	--	-0.207
-0.571	0.733	1.481	-1.271	-1.439	--	0.616	--	0.22	--
0.208	0.637	0.578	0.208	0.401	--	1.194	0.458	--	0.435

-1.974	1.296	1.414	-2.087	-2.471	--	0.658	--	-0.514	--
-1.538	0.410	0.219	-1.122	-0.843	--	1.813	-0.436	--	0.028
-0.085	1.253	1.623	-1.784	-2.147	--	0.657	--	-0.123	--
0.591	1.123	0.466	0.102	0.864	--	1.592	0.599	--	0.668
-0.188	1.183	1.653	-5.544	-5.756	--	0.658	--	-0.142	--
0.051	0.462	-0.081	-0.243	-0.001	--	1.174	0.427	--	0.202
-0.575	2.252	2.206	-1.303	-2.577	--	1.803	--	0.183	--
1.183	2.152	1.277	0.941	2.046	--	2.309	1.203	--	1.195
-1.825	1.339	1.381	-2.556	-5.374	--	0.749	--	-0.272	--
-0.294	0.817	0.041	-0.103	0.490	--	1.611	0.473	--	0.749
-2.363	0.991	1.539	-2.435	-4.531	--	-0.212	--	-1.358	--
-1.385	0.784	0.451	-1.374	-0.592	--	1.465	-0.524	--	0.232
-3.954	-0.165	-0.206	-3.706	-4.765	--	-0.362	--	-1.614	--
-2.652	-0.968	-2.954	-1.445	-2.363	--	0.176	-0.576	--	-0.453
-6.437	0.659	-1.227	-5.156	-6.121	--	0.389	--	-0.782	--
-2.488	-1.363	-2.092	-2.126	-2.571	--	0.935	0.07	--	-0.282
-3.856	-1.200	-0.294	-6.944	-7.852	--	-2.222	--	-2.487	--
-4.813	-2.129	-2.820	-2.669	-3.361	--	0.639	-1.228	--	-1.368
1.103	0.321	0.355	-1.188	-0.491	--	0.466	--	0.206	--
2.235	0.608	-0.403	-0.914	0.628	--	0.731	0.273	--	-0.116
-1.273	-1.522	-2.153	-2.008	-1.071	--	-0.689	--	-2.182	--
-1.245	-2.222	-2.353	0.415	-1.536	--	-2.287	-0.766	--	2.668
1.305	2.062	0.534	1.867	0.115	--	3.307	--	3.125	--
2.824	4.598	-1.491	0.083	0.647	--	3.809	2.195	--	4.757
2.607	1.462	1.460	-1.495	-0.784	--	1.813	--	1.356	--
1.129	-1.175	0.712	-0.818	-0.786	--	-1.021	0.037	--	0.293
0.712	0.003	-1.523	-3.815	-4.945	--	0.896	--	-0.157	--
1.458	-0.681	0.798	-0.227	-0.915	--	-2.165	0.521	--	2.814
0.930	0.228	-0.628	-1.876	-2.000	--	0.126	--	0.074	--
1.633	0.132	0.883	-0.725	-0.869	--	-0.491	0.053	--	1.212
-0.477	-0.341	-0.291	-5.532	-5.488	--	-1.685	--	-0.931	--
0.901	-0.034	1.070	-0.764	-2.983	--	-1.149	0.319	--	3.163
0.864	0.252	-0.962	-0.542	-1.118	--	-0.163	--	1.109	--
1.285	0.754	0.921	-0.967	-0.943	--	-0.467	-0.335	--	0.906
-2.534	-0.181	-3.586	-4.136	-3.948	--	-0.117	--	-3.928	--
0.354	-0.349	-0.532	0.037	-3.215	--	-1.575	1.092	--	1.366
-0.917	0.406	0.396	-2.128	-1.230	--	-0.223	--	-0.605	--
-0.064	0.097	0.447	0.009	-0.782	--	-0.523	0.273	--	0.706
0.455	0.906	-0.366	-3.175	-1.670	--	1.003	--	-0.153	--
1.149	-1.269	0.315	1.014	-0.643	--	-1.035	0.718	--	1.39
0.822	0.333	-0.049	-4.386	-2.918	--	0.111	--	0.127	--
0.774	-1.770	0.821	-0.038	-1.264	--	-1.608	0.147	--	1.184
0.843	0.544	-0.253	-3.625	-1.849	--	1.292	--	0.565	--
0.696	-1.668	0.104	0.284	-0.633	--	-1.405	0.309	--	0.33
0.409	0.162	-0.561	-2.904	-0.786	--	1.150	--	-0.073	--
1.192	-0.954	-0.113	0.490	-0.581	--	-0.810	0.654	--	1.129
0.532	0.484	-0.167	-2.162	-1.007	--	0.909	--	0.189	--

1.063	-0.777	0.202	0.479	-0.242	--	-0.774	0.531	--	1.317
0.920	0.090	-0.441	-4.686	-1.694	--	1.010	--	0.111	--
1.064	-1.262	0.008	-0.220	-0.957	--	-1.147	0.557	--	1.276
0.462	0.283	-0.694	-1.475	-0.061	--	1.030	--	0.041	--
1.002	-0.147	-0.136	0.662	-0.582	--	-0.942	0.299	--	0.534
1.170	0.984	3.042	1.652	-0.334	--	0.744	--	3.244	--
1.563	1.899	1.392	-0.585	3.497	--	0.376	-0.877	--	2.549
0.605	1.418	0.609	0.987	-0.946	--	-0.088	--	2.523	--
0.816	0.082	0.326	-0.185	-0.052	--	-0.086	-0.025	--	-0.04
-0.668	0.005	0.546	-0.421	-0.363	--	-0.109	--	0.818	--
-0.922	-0.055	-0.115	-0.727	-0.425	--	-0.744	-0.311	--	0.01
-0.177	2.798	2.363	-0.268	-1.868	--	2.001	--	4.046	--
1.475	1.867	1.803	-0.021	1.687	--	2.658	1.29	--	2.53
-0.428	1.068	0.161	-0.550	-0.469	--	0.122	--	0.012	--
0.382	-0.079	0.029	-0.026	0.199	--	0.272	0.02	--	0.346
-0.218	0.749	0.543	-0.538	-0.167	--	0.067	--	0.156	--
0.096	0.065	-0.015	-0.003	0.000	--	0.054	0.017	--	0.096
0.000	0.232	0.000	-0.042	0.000	--	0.000	--	0	--
-0.096	0.171	-0.204	-0.290	-0.041	--	-0.020	0.021	--	0.29
-0.608	0.563	0.418	-0.120	-0.506	--	0.358	--	0.619	--
-0.418	-3.049	0.012	-0.183	-3.307	--	1.078	-1.393	--	0.01
-0.396	0.284	0.443	-0.884	-0.595	--	0.262	--	0.245	--
-1.242	-1.145	-1.235	-0.297	-1.543	--	0.174	-0.582	--	0.293
0.679	0.165	-0.121	0.178	0.373	--	0.264	--	0.543	--
-1.315	0.030	0.368	-2.566	1.129	--	-0.210	1.018	--	0.407
1.997	0.430	0.454	0.493	1.920	--	-0.624	--	1.187	--
-0.008	-0.072	-0.199	-1.050	0.221	--	-0.434	-0.306	--	-0.747
2.281	0.494	0.769	1.006	2.710	--	-0.209	--	2.618	--
1.282	0.209	0.772	-0.885	1.414	--	-0.342	-0.233	--	-0.06
1.700	0.690	1.629	2.055	1.735	--	0.871	--	2.289	--
-0.445	0.200	0.540	-1.633	-0.182	--	-0.316	-0.796	--	-2.761
0.695	0.710	1.393	1.724	1.466	--	1.146	--	1.237	--
-0.363	0.500	0.946	-0.853	0.624	--	-0.096	-0.609	--	-0.649
1.540	2.195	2.023	1.965	1.656	--	1.581	--	1.226	--
2.381	2.466	3.198	0.170	2.881	--	1.716	-2.02	--	0.821
1.247	1.118	1.839	1.215	1.507	--	1.249	--	1.774	--
0.827	0.643	1.718	-0.054	1.265	--	-0.008	-0.292	--	0.899
-0.042	0.192	0.510	-0.135	-0.428	--	-0.025	--	0.227	--
-0.274	-0.247	0.154	-1.246	-0.192	--	-0.605	-0.852	--	-0.202
-0.198	0.146	0.224	0.073	0.007	--	-0.045	--	0.096	--
-0.155	0.198	0.054	-0.479	0.339	--	-0.113	-0.314	--	-0.344
0.911	0.546	1.295	0.719	0.879	--	0.591	--	0.819	--
1.198	1.203	1.534	0.418	0.919	--	-0.028	0.3	--	1.114

98	99	100	102	103	104	105	106
130.80	--	123.80	88.00	--	131.40	--	--
138.00	--	140.60	127.40	--	102.40	--	--
13.30	--	19.80	19.00	--	12.60	--	--
14.90	--	14.60	14.50	--	14.00	--	--
--	--	15.52	16.58	14.50	13.24	--	11.96
21.66	--	21.43	22.99	--	24.43	--	--
20.76	--	20.23	30.78	--	28.15	--	--
27.63	--	28.09	32.71	--	30.04	--	--
27.67	--	25.99	43.67	--	29.52	--	--
84.90	--	78.29	74.13	--	79.98	--	--
82.80	--	83.26	64.23	--	78.47	--	--
6.00	--	58.00	48.00	--	6.00	--	--
2.00	--	41.00	34.00	--	6.00	--	--
--	--	164.00	152.00	144.00	124.00	--	124.00
20.23	--	41.33	29.58	--	--	--	--
--	--	27.92	17.50	--	2.23	--	--
--	--	23.73	27.26	12.26	24.32	--	56.78
1	--	2	2	2	1	2	--
1	--	2	2	2	1	2	--
--	--	2	2	1	--	--	--
-0.445	--	-0.448	0.111	--	-0.662	--	--
-0.062	--	0.046	0.301	--	-0.191	--	--
-0.126	--	-0.467	0.151	--	0.044	--	--
-0.157	--	0.106	0.193	--	-0.107	--	--
-0.303	--	-0.217	0.352	--	-0.425	--	--
0.035	--	0.447	1.028	--	0.411	--	--
0.238	--	-0.400	-0.169	--	0.176	--	--
3.338	--	-2.809	3.243	--	-0.214	--	--
0.036	--	-0.038	0.216	--	0.013	--	--
0.129	--	0.708	0.277	--	-0.222	--	--
0.234	--	-0.047	-0.028	--	0.315	--	--
-0.078	--	0.651	0.111	--	-0.046	--	--
-0.048	--	-0.419	-0.292	--	0.082	--	--
-0.046	--	0.450	0.237	--	-0.125	--	--
-0.087	--	-0.280	-0.178	--	0.037	--	--
-0.058	--	0.431	0.259	--	-0.197	--	--
-0.168	--	-0.221	-0.279	--	0.083	--	--
-0.064	--	0.403	0.213	--	-0.186	--	--
-0.100	--	-0.041	-0.229	--	0.241	--	--
-0.006	--	0.549	0.239	--	-0.037	--	--
0.005	--	-0.353	-0.308	--	0.039	--	--
-0.106	--	0.433	0.069	--	-0.229	--	--
-0.444	--	-0.797	-0.450	--	-0.679	--	--
-0.480	--	-0.005	-0.130	--	-0.376	--	--
0.334	--	0.040	-0.107	--	0.440	--	--

-0.089	--	0.726	0.263	--	0.056	--	--
-1.061	--	-1.713	-0.056	--	-0.488	--	--
-2.437	--	2.635	2.654	--	-0.625	--	--
-1.440	--	-1.974	-3.280	--	-2.116	--	--
-2.911	--	-1.263	-0.821	--	-0.588	--	--
-1.294	--	-0.787	-0.556	--	-0.232	--	--
0.503	--	0.152	0.241	--	-0.060	--	--
-3.744	--	-7.607	-0.473	--	-0.342	--	--
0.924	--	0.401	0.255	--	0.046	--	--
-7.021	--	-6.753	-10.548	--	-0.181	--	--
6.993	--	10.733	-0.073	--	7.089	--	--
0.067	--	-0.008	0.012	--	0.131	--	--
1.610	--	1.125	0.130	--	0.568	--	--
-0.016	--	0.108	-0.016	--	-0.045	--	--
2.684	--	1.394	0.883	--	0.745	--	--
-0.028	--	-0.514	0.506	--	0.428	--	--
0.145	--	0.457	0.391	--	-0.275	--	--
0.192	--	-0.153	0.384	--	0.374	--	--
0.020	--	0.440	0.256	--	-0.183	--	--
-0.193	--	-0.287	0.135	--	0.119	--	--
0.054	--	0.450	0.230	--	-0.254	--	--
0.182	--	-0.178	0.290	--	0.633	--	--
0.069	--	0.931	0.520	--	-0.232	--	--
-0.127	--	-0.325	0.107	--	-0.055	--	--
0.144	--	0.177	0.030	--	-0.114	--	--
-0.063	--	-0.262	0.154	--	-0.084	--	--
0.071	--	0.175	0.123	--	-0.084	--	--
-0.103	--	-0.210	0.284	--	0.215	--	--
0.252	--	0.258	0.166	--	-0.093	--	--
-0.168	--	-0.428	0.032	--	0.151	--	--
-0.171	--	0.157	0.000	--	-0.428	--	--
-0.754	--	-2.761	-2.611	--	2.212	--	--
-2.129	--	-1.843	0.375	--	0.657	--	--
-0.808	--	-2.904	-2.199	--	2.265	--	--
-2.776	--	-2.421	0.319	--	0.693	--	--
-0.103	--	-4.642	-4.827	--	2.943	--	--
-1.832	--	-2.060	1.185	--	1.096	--	--
-1.443	--	-7.885	-7.882	--	2.783	--	--
-4.041	--	-3.474	0.164	--	0.820	--	--
0.081	--	-0.736	-0.680	--	1.865	--	--
-1.777	--	-1.397	0.107	--	0.447	--	--
0.512	--	-3.418	-2.607	--	1.809	--	--
0.412	--	-0.971	-0.068	--	1.389	--	--
0.228	--	-3.269	-2.369	--	1.737	--	--
0.340	--	-0.947	-0.142	--	1.279	--	--
0.655	--	-1.038	-0.217	--	1.517	--	--
1.312	--	0.138	0.295	--	1.379	--	--

0.613	--	-2.284	-1.519	--	3.185	--	--
0.042	--	-1.311	-0.213	--	1.954	--	--
1.109	--	-0.142	-0.809	--	1.624	--	--
1.826	--	0.412	0.522	--	1.322	--	--
0.765	--	-2.571	-1.771	--	2.092	--	--
0.935	--	-0.081	0.751	--	0.844	--	--
1.657	--	-2.249	-1.569	--	2.176	--	--
2.933	--	1.026	0.754	--	2.302	--	--
0.365	--	-5.425	-2.554	--	2.013	--	--
1.260	--	-0.344	0.425	--	1.404	--	--
1.074	--	-4.407	-3.245	--	2.013	--	--
1.178	--	-1.028	-0.279	--	1.418	--	--
-0.458	--	-5.617	-3.823	--	1.441	--	--
-0.910	--	-1.981	-0.374	--	0.517	--	--
-0.926	--	-6.337	-6.336	--	2.000	--	--
-1.885	--	-3.858	0.147	--	2.470	--	--
-2.609	--	-8.157	-7.019	--	2.924	--	--
-2.669	--	-3.121	-0.639	--	0.688	--	--
0.747	--	2.098	0.545	--	0.937	--	--
1.182	--	1.723	0.942	--	0.673	--	--
-2.330	--	-1.896	3.261	--	-2.119	--	--
-1.365	--	-1.841	3.792	--	1.158	--	--
1.320	--	1.388	4.510	--	2.417	--	--
1.790	--	-0.498	5.946	--	3.304	--	--
-0.182	--	1.013	3.281	--	0.771	--	--
-0.349	--	0.393	1.312	--	0.572	--	--
-1.661	--	-4.510	0.545	--	-1.839	--	--
0.578	--	0.144	1.150	--	0.502	--	--
-0.589	--	-3.164	1.143	--	-0.866	--	--
0.041	--	0.207	0.490	--	-0.108	--	--
0.206	--	-5.065	-0.265	--	-0.478	--	--
1.058	--	-0.815	0.973	--	0.357	--	--
-1.363	--	-2.375	1.707	--	-2.731	--	--
0.650	--	-0.101	-0.353	--	-0.789	--	--
-3.944	--	-4.039	0.882	--	-3.890	--	--
-0.266	--	-2.548	2.463	--	0.482	--	--
-0.274	--	-1.518	0.430	--	-0.386	--	--
0.354	--	-0.189	0.456	--	0.272	--	--
-1.157	--	-2.368	2.133	--	-0.133	--	--
0.171	--	0.807	1.297	--	0.594	--	--
-1.277	--	-2.602	1.461	--	-0.641	--	--
0.091	--	0.981	0.671	--	0.345	--	--
-0.892	--	-1.672	1.509	--	-0.473	--	--
0.118	--	0.582	0.577	--	0.400	--	--
-1.055	--	-1.091	1.740	--	-0.811	--	--
-0.084	--	0.764	1.162	--	0.415	--	--
-0.571	--	-0.651	2.026	--	-0.638	--	--

0.384	--	1.057	0.933	--	0.427	--	--
-1.292	--	-1.609	2.263	--	-0.874	--	--
-0.121	--	0.650	2.251	--	0.078	--	--
-0.944	--	-2.280	1.221	--	-0.322	--	--
-0.264	--	0.503	0.975	--	0.230	--	--
1.573	--	1.910	1.189	--	3.023	--	--
1.304	--	2.251	-0.179	--	2.260	--	--
0.644	--	1.647	0.991	--	0.562	--	--
0.251	--	0.820	0.835	--	0.036	--	--
0.331	--	-0.080	0.290	--	0.577	--	--
-0.516	--	-0.514	-0.014	--	-0.225	--	--
2.290	--	2.376	2.624	--	2.458	--	--
1.394	--	1.549	1.667	--	2.716	--	--
-0.349	--	-0.474	-0.084	--	0.322	--	--
0.215	--	0.229	0.192	--	0.169	--	--
0.402	--	-0.247	-0.294	--	0.249	--	--
0.038	--	0.120	0.054	--	-0.026	--	--
0.000	--	0.000	0.035	--	0.000	--	--
-0.263	--	-0.140	-0.313	--	-0.187	--	--
0.161	--	-0.206	0.417	--	0.250	--	--
-1.744	--	-2.152	-0.122	--	-0.053	--	--
0.060	--	-0.046	-0.076	--	0.045	--	--
-0.381	--	0.080	0.190	--	0.186	--	--
0.577	--	1.275	0.809	--	-0.016	--	--
-0.031	--	0.109	0.060	--	0.284	--	--
0.585	--	2.137	1.610	--	0.493	--	--
-0.027	--	-0.027	-0.870	--	0.025	--	--
1.131	--	3.203	1.038	--	0.449	--	--
1.197	--	0.628	-0.258	--	0.765	--	--
1.784	--	3.336	3.063	--	1.902	--	--
-0.062	--	-0.272	-1.134	--	0.796	--	--
2.013	--	2.195	1.711	--	1.795	--	--
0.947	--	-0.318	-0.877	--	1.203	--	--
2.497	--	2.783	1.818	--	1.361	--	--
3.536	--	1.043	-1.588	--	3.125	--	--
2.595	--	2.564	1.643	--	1.684	--	--
2.040	--	0.960	-0.253	--	1.605	--	--
0.220	--	0.276	-0.069	--	0.135	--	--
0.110	--	-0.043	-1.065	--	0.204	--	--
0.202	--	0.209	-0.082	--	0.180	--	--
0.120	--	-0.082	-0.558	--	0.159	--	--
1.422	--	1.488	0.822	--	0.282	--	--
1.722	--	-0.061	-0.093	--	0.741	--	--
